# Association between Red Cell Distribution Width (RDW)-related inflammatory biomarkers and prognosis in ICU Cirrhosis patients: Evidence from the MIMIC-IV

**DOI:** 10.1371/journal.pone.0352023

**Published:** 2026-06-26

**Authors:** Xiaodong Zhu, Chanchan Lin, Xiaoqiang Liu, Zicheng Huang, Yingyi Li

**Affiliations:** Department of Gastroenterology, The First Hospital of Quanzhou Affiliated to Fujian Medical University, Quanzhou, China; University of Cape Town Faculty of Science, SOUTH AFRICA

## Abstract

**Objectives:**

Cirrhosis signifies a critical phase in liver disease with high mortality, particularly in patients admitted to intensive care unit (ICU). Red cell distribution width (RDW) demonstrates prognostic value in various conditions, with evidence linking it to inflammation and adverse outcomes in liver disease. However, the predictive utility of RDW and its derived indices in patients with cirrhosis admitted to ICU remains undetermined. This study investigated the associations between RDW-related biomarkers and prognosis in critically ill cirrhosis patients.

**Methods:**

A retrospective analysis used data from the Medical Information Mart for Intensive Care IV (MIMIC-IV) database, including 1,871 adults with cirrhosis at first ICU admission. The analyzed biomarkers were RDW, RDW-to-albumin ratio (RAR), RDW-to-platelet ratio (RPR), and hemoglobin-to-RDW ratio (HRR). The primary outcome was 30-day all-cause mortality; secondary outcomes were 90-day, and 365-day mortality. Adjusted multivariate Cox regression models determined biomarker-mortality associations. Receiver operating characteristic (ROC) curve analyses evaluated predictive performance of the biomarkers.

**Results:**

The cohort had a mean age of 58.8 ± 12.3 years and 64.8% of the patients were male. The mortality rates were 31.8% (30-day), 39.3% (90-day), and 48.0% (365-day). Mortality was consistently higher in high versus low RDW groups. After multivariable adjustment, all biomarkers were independently associated with 30-day, 90-day, and 365-day mortality, respectively. When expressed per SD, RDW (HR 1.41, 95% CI 1.30–1.52), RAR (HR 1.17, 95% CI 1.11–1.24), RPR (HR 1.19, 95% CI 1.11–1.28), and HRR (HR 0.52, 95% CI 0.44–0.61) were each significantly associated with 30-day mortality. ROC analysis revealed RDW had the highest predictive value (AUC 63.3%), followed by HRR (AUC 61.7%), RAR (AUC 59.6%), and RPR (AUC 55.6%). RDW demonstrated the highest discriminative ability for 30-day mortality, with incremental predictive value when combined with established scores (absolute AUC increase 0.68–1.58%).

**Conclusion:**

RDW-related biomarkers demonstrate statistically significant but modest associations with mortality in ICU cirrhosis patients. RDW and HRR showed the strongest predictive capabilities among the four indices, though standalone discrimination was limited. These accessible, cost-effective biomarkers may serve as adjunctive prognostic markers when combined with established scoring systems. However, further prospective validation is required before clinical implementation, and dynamic changes in these biomarkers warrant investigation in future studies.

## 1 Introduction

Cirrhosis is a progressive liver disease characterized by the replacement of the normal liver parenchyma with fibrotic tissue and regenerative nodules, ultimately leading to portal hypertension and liver dysfunction. The disease progresses from an asymptomatic phase, known as compensated cirrhosis, to a symptomatic phase, known as decompensated cirrhosis [1]. Decompensated cirrhosis represents a critical phase of liver disease which the liver can no longer perform its essential functions, leading to severe complications (such as ascites, variceal bleeding, and hepatic encephalopathy) and a high mortality rate. Despite advancements in medical therapies, the prognosis of patients with decompensated cirrhosis remains poor, necessitating a deeper understanding of the disease and the development of more effective treatment strategies [2]. Furthermore, the management of critically ill patients with cirrhosis in the intensive care setting presents additional challenges [3]. Thus, the importance of identifying reliable prognostic markers in this patient population cannot be overstated. Patients with cirrhosis in the intensive care unit (ICU) represent a particularly vulnerable group with high mortality rates [4]. Early risk stratification and an accurate prognostic assessment are crucial for optimal patient management and resource allocation [5].

Red cell distribution width (RDW) is a routinely measured hematological parameter that reflects heterogeneity in the size of circulating red blood cells (RBCs) [6]. In recent years, RDW has emerged as a valuable prognostic biomarker across various clinical conditions, particularly in critical care settings [7]. The clinical significance of RDW extends beyond its traditional role in differentiating types of anaemia, as accumulating evidence suggests that it is closely associatied with inflammatory processes and adverse outcomes [8]. Inflammation plays a pivotal role in the pathogenesis and progression of liver cirrhosis. Recent studies have demonstrated that elevated RDW levels are significantly associated with poor outcomes in patients with liver diseases [9]. The relationship between RDW and inflammation has been well documented, with several studies showing strong correlations between RDW and conventional inflammatory markers such as C-reactive protein (CRP) and erythrocyte sedimentation rate (ESR) [10].

However, the predictive value of RDW and its derived indices in patients with cirrhosis admitted to the ICU remain unclear. Therefore, this study aimed to investigate the associations between RDW-related inflammatory biomarkers and prognosis in critically ill patients with cirrhosis using the Medical Information Mart for Intensive Care IV (MIMIC-IV) database.

## 2 Method

### 2.1 Data sources and study design

This study was a retrospective cohort analysis that utilized data from a large critical care database, the Medical Information Mart for Intensive Care IV (MIMIC-IV). MIMIC-IV v3.0 is a comprehensive, single-center longitudinal database that encompasses data on 364 627 patients admitted to the Beth Israel Deaconess Medical Center, including 94 458 intensive care unit (ICU) stays, spanning the period from 2008 to 2022 (https://mimic.mit.edu) [11]. This study was approved by the institutional review boards of both the Massachusetts Institute of Technology and BIDMC. The authors were given access to the MIMIC database (Record ID: 62389476). All procedures performed in the present study were in accordance with the principles outlined in the 1964 Helsinki Declaration and its later amendments. Ethical approval for secondary data analysis was waived because MIMIC-IV data are publicly available, and all patient data are de-identified.

### 2.2 Study population selection criteria

**Fig 1:**
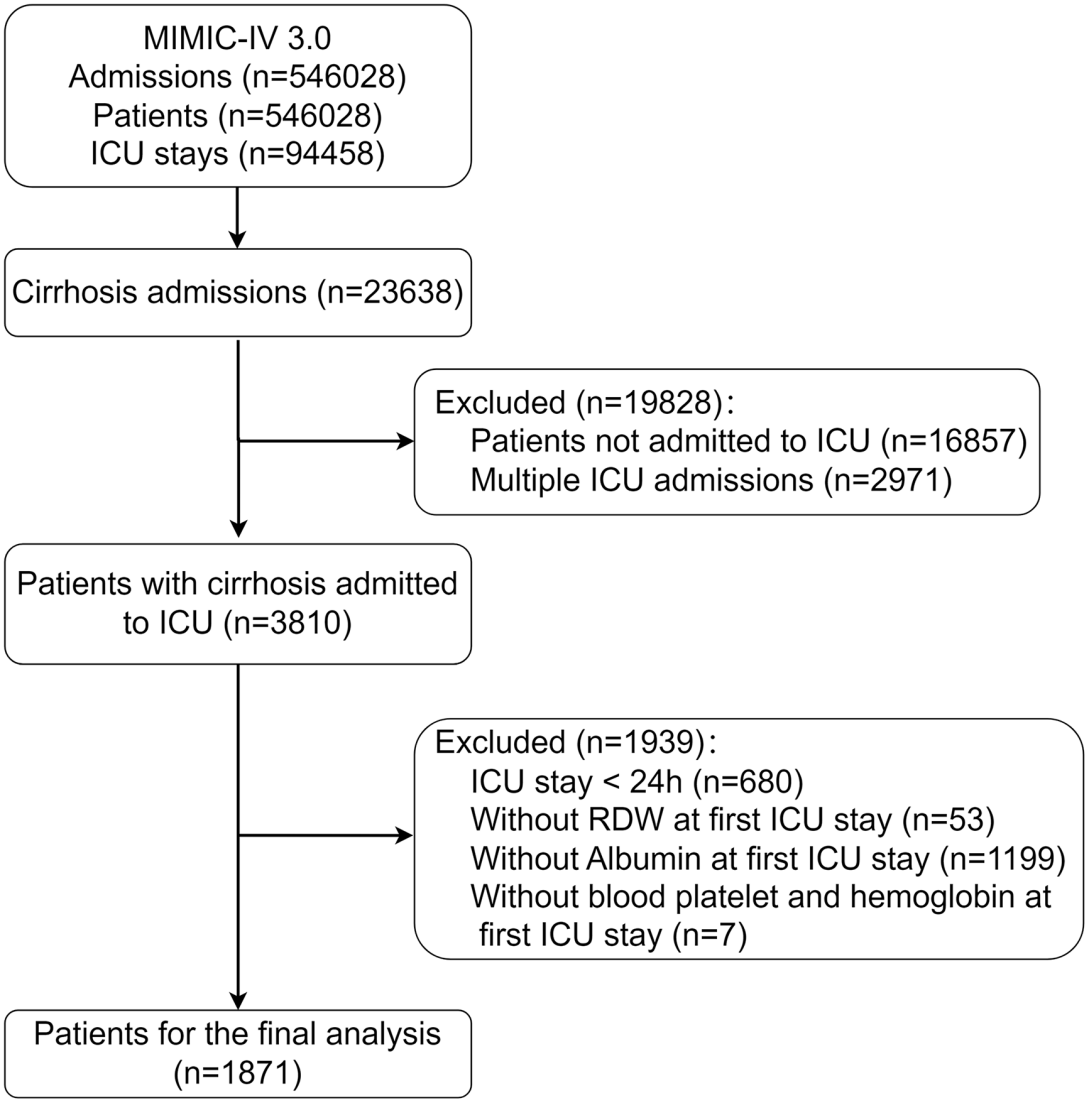
Flow of the study. Abbreviations: RDW, red cell distribution width; MIMIC-IV, Medical Information Mart for Intensive Care IV; ICU, Intensive Care Unit.

The inclusion criteria were: (1) patients diagnosed with cirrhosis based on ICD-9 and ICD-10 codes and (2) first-time ICU admission. All patients enrolled in the study were aged 18 years or older. Exclusion criteria: (1) absence of RDW, albumin, platelet, and hemoglobin results within 24 h after ICU admission, (2) lack of data on mortality. After exclusions for missing laboratory data (RDW n = 53, albumin n = 1,199, platelets n = 3, hemoglobin n = 7), the study finally included data from the MIMIC-IV database of 1,871 patients with cirrhosis who were admitted to ICU (Fig 1). A comparison of baseline characteristics between the 1,939 excluded patients and the 1,871 patients ultimately included in the study is presented in Supplementary Table 1 in S1 File.

### 2.3 RDW-related biomarkers

The following RDW-related biomarkers were analyzed: RDW, RDW-to-albumin ratio (RAR), RDW-to-platelet ratio (RPR), and hemoglobin-to-RDW ratio (HRR). RDW (%), albumin (g/dL), platelets (10^9/L), and hemoglobin (g/dL) were measured within 24 h after ICU admission. The median of each biomarker was used as the cut-off point to divide the patients into two groups: namely low and high groups. The median values were as follows: RDW 16.9%, RAR 5.86, RPR 0.17, HRR 0.55. To account for the different measurement scales of these four biomarkers and to ensure interpretable cross-biomarker comparisons, each biomarker was additionally converted to a z-score (z = [value − mean]/ standard deviation) using the cohort mean and standard deviation. The z-scored variables were used in parallel Cox regression models to generate per-standard-deviation (per-SD) hazard ratios.

### 2.4 Outcomes

The primary outcome was 30-day all-cause mortality, defined as death from any cause within 30 days after ICU admission. The secondary outcomes were 90-day and 365-day all-cause mortality. Follow-up time was calculated from the date of ICU admission. Additionally, we assessed five cirrhosis-related complications: variceal hemorrhage, ascites, spontaneous bacterial peritonitis, hepatic encephalopathy, and hepatorenal syndrome.

### 2.5 Data extraction

Structured Query Language (SQL) and the PostgreSQL tool were used to extract data from the MIMIC-IV database. The extracted data were patient demographics, vital signs, laboratory parameters, clinical evaluations, and treatment. Demographic information included age, sex, race, and body mass index (BMI). The vital parameters included respiratory rate, pulse oximetry, temperature, blood pressure, and heart rate. Co-morbidities included hypertension, diabetes, chronic pulmonary disease, myocardial infarction, congestive heart failure, renal disease, cerebrovascular disease, malignant cancer, malnutrition, and peptic ulcer disease. Clinical assessments included the Charlson Comorbidity Index, Acute Physiology Score III (APS III), and etiology of cirrhosis. Therapeutic management included albumin, mechanical ventilation, vasopressin, and renal replacement therapy (RRT). In addition to RDW-related parameters, other laboratory indicators included white blood cells, total bilirubin, aspartate aminotransferase (AST), alanine aminotransferase (ALT), creatinine, urea nitrogen, glucose, sodium, potassium, chloride, calcium, anion gap, and lactate. Details regarding missing data for key covariates in the included population are presented in Supplementary Table 2 in S1 File.

### 2.6 Statistical analyses

Continuous and normally distributed variables were represented as mean ± standard deviation (SD). For variables that were continuous and non-normally distributed, the median with interquartile range (IQR) was presented. Categorical variables were reported as frequencies and their respective percentages. The cumulative rates of death were compared using Kaplan – Meier curves. To resolve missing data issues, multiple imputations were performed using chained equations with 50 imputations, to secure unbiased estimates of the association between RDW and the outcome.

The impact of RDW-related indices on different outcomes was assessed through binary Cox regression models that were adjusted for key covariates [12–15]. For each biomarker, two parallel Cox models were fitted: one using the original-scale variable (yielding per-unit hazard ratios) and one using the z-scored variable (yielding per-SD hazard ratios). We selected covariates based on clinical judgment and previous literature. To assess potential confounders, we introduced each covariate individually into the baseline Cox regression model and compared changes in the regression coefficients. Covariates that caused a change of more than 10% in the initial regression coefficient were retained. Subsequently, we excluded variables with a variance inflation factor (VIF) ≥ 5 and any potential mediators. The final set of retained covariates was included in the fully adjusted model. The covariates adjusted for in the final fully adjusted model included sex, age, race, BMI, Charlson Comorbidity Index, vasopressin use, albumin use, mechanical ventilation, renal replacement therapy, hemoglobin, hematocrit, albumin, potassium, and lactate levels. For ratio-based biomarkers, their constituent laboratory variables were excluded from the respective adjustment models to avoid overadjustment and collinearity (hemoglobin was excluded from the HRR model; albumin was excluded from the RAR model).

We employed Cox proportional hazards regression models incorporating cubic spline functions and smooth curve fitting to identify potential non-linear relationships between various RDW-derived indices and the outcomes. If a non-linear association was detected, inflection points were first determined using a recursive algorithm, followed by the construction of piecewise Cox proportional hazards regression models on either side of the identified inflection points. The subgroup analyses were performed using stratified Cox proportional hazards models. Interaction among subgroups was inspected by the likelihood ratio test. Sensitivity analysis was performed to assess the robustness of all outcomes to unmeasured confounding variables using the E-value methodology of VanderWeele and Ding [16]. Receiver Operating Characteristic (ROC) analysis was used to evaluate the ability of RDW, RAR, RPR, and HRR to predict mortality at 30, 90 and 365 days after ICU admission. The ROC-derived thresholds were determined by maximizing the Youden index (sensitivity + specificity – 1) on the ROC curve, thereby achieving the optimal balance between sensitivity and specificity for clinical application. Moreover, we further compared the predictive capabilities of the APS III, SAPS II, MELD, and SOFA scores, as well as those of their respective combined models with RDW, for the primary outcome. The sensitivity and specificity were determined for each indicator, and the area under the curve (AUC) was calculated. Statistical analyses were performed using R software (http://www.r-project.org, The R Foundation) and Free Statistics software version 2.1.0.

## 3 Results

### 3.1 Baseline characteristics

The study included 1,871 cirrhosis patients, with a mean age of 58.8 ± 12.3 years and 64.8% were male patients. Baseline demographic and clinical characteristics stratified by RDW group are presented in Table 1. The high RDW group had lower blood pressure, lower temperature, higher APS III scores, more alcohol related-cirrhosis (60.3%), more therapeutic albumin administration and required more RRT. The proportion of patients with hypertension, diabetes, chronic pulmonary disease, and cancer was lower in the high RDW group; however, the proportion of peptic ulcer disease and malnutrition was higher in the high RDW group. The levels of white blood cells, total bilirubin, creatinine, urea nitrogen, anion gap, and lactate were significantly higher in the high RDW group than in the low RDW group. However, the levels of hemoglobin, platelet count, ALT, glucose, and chloride in the high RDW group were significantly lower than those in the low RDW group (Table 1).

**Table 1:** The basic demographics and clinical characteristics in patients with cirrhosis.

Characteristics	MIMIC cohort (n = 1871)	Low RDW group (n = 924)	High RDW group (n = 947)	P-value
**Demographics**				
Male, n (%)	1212 (64.8)	642 (69.5)	570 (60.2)	< 0.001
Age, years	58.8 ± 12.3	60.1 ± 11.9	57.5 ± 12.5	< 0.001
Race (white), n (%)	1185 (75.0)	600 (75.9)	585 (74.1)	0.433
BMI (kg/m^2^)	29.6 ± 7.8	29.4 ± 7.8	29.7 ± 7.7	0.557
**Vital signs**				
Heart rate (beats/min)	88.5 ± 16.3	88.0 ± 16.8	88.9 ± 15.8	0.277
SBP (mmHg)	113.3 ± 16.0	115.4 ± 16.7	111.3 ± 14.9	< 0.001
DBP (mmHg)	61.4 ± 10.2	62.6 ± 10.6	60.3 ± 9.7	< 0.001
MBP (mmHg)	76.1 ± 10.7	77.5 ± 11.1	74.7 ± 10.1	< 0.001
Respiratory rate (beats/min)	19.0 ± 4.2	19.0 ± 4.2	19.1 ± 4.3	0.816
Temperature (°C)	36.8 ± 0.5	36.9 ± 0.5	36.7 ± 0.5	< 0.001
Spo2 (%)	96.9 ± 2.2	96.9 ± 2.1	96.9 ± 2.2	0.887
**Comorbidities, n(%)**				
Hypertension	877 (46.9)	460 (49.8)	417 (44.0)	0.013
Diabetes	532 (28.4)	282 (30.5)	250 (26.4)	0.048
Myocardial infarct	133 (7.1)	71 (7.7)	62 (6.5)	0.339
Congestive heart failure	297 (15.9)	138 (14.9)	159 (16.8)	0.272
Cerebrovascular disease	128 (6.8)	73 (7.9)	55 (5.8)	0.073
Chronic pulmonary disease	383 (20.5)	208 (22.5)	175 (18.5)	0.031
Renal disease	360 (19.2)	169 (18.3)	191 (20.2)	0.303
Malignant cancer	339 (18.1)	203 (22.0)	136 (14.4)	< 0.001
Peptic ulcer disease	151 (8.1)	55 (6.0)	96 (10.1)	< 0.001
Malnutrition	491 (26.2)	194 (21.0)	297 (31.4)	< 0.001
**Clinical assessment and treatments, n (%)**				
APS III	68.9 ± 29.4	62.6 ± 28.7	75.0 ± 28.8	< 0.001
Charlson Comorbidity Index	6.9 ± 2.7	6.8 ± 2.8	6.9 ± 2.7	0.957
Etiology, alcohol cirrhosis	1026 (54.8)	455 (49.2)	571 (60.3)	< 0.001
Albumin use	1253 (67.1)	595 (64.5)	658 (69.6)	0.019
Mechanical ventilation	1085 (58.0)	533 (57.7)	552 (58.3)	0.791
Vasopressin	790 (42.2)	376 (40.7)	414 (43.7)	0.185
RRT	326 (17.4)	112 (12.1)	214 (22.6)	< 0.001
**Laboratory Indicators**				
RDW (%)	17.4 ± 3.0	15.1 ± 1.1	19.7 ± 2.4	< 0.001
RAR	6.2 ± 2.1	5.3 ± 1.4	7.1 ± 2.3	< 0.001
RPR	0.2 (0.1, 0.3)	0.1 (0.1, 0.2)	0.2 (0.1, 0.3)	< 0.001
HRR	0.6 ± 0.2	0.7 ± 0.2	0.5 ± 0.1	< 0.001
White blood cells (10^9^/L)	11.9 ± 7.9	11.4 ± 7.5	12.4 ± 8.3	0.008
Hemoglobin (g/dL)	9.6 ± 2.1	10.3 ± 2.1	8.9 ± 1.9	< 0.001
Platelet count (10^9^/L)	100.0 (65.5, 151.5)	108.0 (72.8, 161.0)	90.0 (60.0, 143.5)	< 0.001
Total bilirubin (mg/dL)	3.3 (1.5, 8.5)	2.2 (1.1, 4.4)	5.5 (2.3, 13.6)	< 0.001
Asparate aminotransferase (U/L)	78.0 (43.0, 212.0)	73.5 (41.0, 245.8)	82.0 (45.0, 191.5)	0.370
Alanine aminotransferase (U/L)	37.0 (21.0, 96.5)	41.0 (21.0, 117.0)	36.0 (21.0, 80.5)	0.019
Albumin (g/dL)	3.0 ± 0.7	3.0 ± 0.6	2.9 ± 0.7	0.410
Creatinine (mg/dL)	1.2 (0.8, 2.2)	1.1 (0.8, 1.9)	1.4 (0.8, 2.6)	< 0.001
Urea nitrogen (mg/dL)	27.0 (16.0, 49.0)	22.5 (14.0, 39.0)	32.0 (18.0, 55.0)	< 0.001
Glucose (mmol/L)	150.9 ± 89.4	157.7 ± 87.8	144.3 ± 90.4	0.001
Sodium (mmol/L)	136.4 ± 6.8	136.6 ± 6.9	136.1 ± 6.7	0.140
Potassium (mmol/L)	4.3 ± 0.9	4.3 ± 0.8	4.3 ± 0.9	0.306
Chloride (mmol/L)	102.0 ± 7.8	102.4 ± 7.7	101.6 ± 8.0	0.046
Calcium (mmol/L)	8.4 ± 1.1	8.3 ± 1.1	8.4 ± 1.1	0.315
Anion gap (mmol/L)	16.5 ± 6.2	16.2 ± 6.3	16.9 ± 6.1	0.040
Lactate (mmol/L)	2.6 (1.7, 4.3)	2.4 (1.6, 4.1)	2.7 (1.8, 4.4)	0.001
**Outcome**, n (%)				
30-day mortality	595 (31.8)	216 (23.4)	379 (40.0)	< 0.001
90-day mortality	736 (39.3)	276 (29.9)	460 (48.6)	< 0.001
365-day mortality	898 (48.0)	357 (38.6)	541 (57.1)	< 0.001
Variceal bleeding	221 (11.8)	82 (8.9)	139 (14.7)	< 0.001
Ascites	972 (52.0)	385 (41.7)	587 (62.0)	< 0.001
Hepatorenal syndrome	322 (17.2)	103 (11.1)	219 (23.1)	< 0.001
Hepatic encephalopathy	190 (10.2)	79 (8.5)	111 (11.7)	0.023
Spontaneous peritonitis	220 (11.8)	73 (7.9)	147 (15.5)	< 0.001

Abbreviations: BMI, body mass index; SBP, systolic blood pressure; DBP, diastolic blood pressure; MBP, mean arterial pressure; SpO_2_, pulse oximetry; APS III, Acute Physiology Score III; RRT, renal replacement therapy; RDW, red cell distribution width; RAR, red cell distribution width-to-albumin ratio; RPR, red cell distribution width-to-platelet ratio; HRR, hemoglobin-to-red cell distribution width.

### 3.2 Short- and long-term mortality

In 1,871 patients, 30-day mortality, 90-day mortality, and 365-day mortality rates were 31.8%, 39.3%, and 48.0%, respectively. Among these, 11.8% had variceal bleeding, 52.0% had ascites, 17.2% had hepatorenal syndrome, 10.2% had hepatic encephalopathy, and 11.8% had spontaneous bacterial peritonitis (Table 1). Supplementary Figs. 1-3 in S1 File show the results of the Kaplan-Meier method concerning 30-day, 90-day and 365-day mortality analyses according to RDW, RAR, RPR, and HRR levels. In the high RDW, high RAR, high RPR, and low HRR groups, the 30-day, 90-day, and 365-day mortality rates were all significantly higher than those in the reference group.

### 3.3 Association between RDW-related biomarkers and mortality

To explore the association between RDW-related inflammatory biomarkers and mortality, we conducted Cox regression analysis. The results of univariate cox analyses of mortality rates at different times are presented in Supplementary Table 3 in S1 File. Table 2 presents the results of the fully adjusted Cox regression models. After multivariable adjustment, all four biomarkers were significantly associated with mortality across all three endpoints (30-day, 90-day, and 365-day). To address the different measurement scales and ensure interpretable cross-biomarker comparisons, both per-unit and per-SD hazard ratios are reported. For the primary outcome of 30-day mortality, when expressed per SD increase, RDW (HR 1.41, 95% CI 1.30–1.52; P < 0.001), RAR (HR 1.17, 95% CI 1.11–1.24; P < 0.001), RPR (HR 1.19, 95% CI 1.11–1.28; P < 0.001), and HRR (HR 0.52, 95% CI 0.44–0.61; P < 0.001) each demonstrated significant associations. Similar patterns were observed for 90-day and 365-day mortality (Table 2). Across all endpoints, RDW and HRR consistently showed the strongest per-SD effect sizes, followed by RPR and RAR.

**Table 2:** The association of red cell distribution width and prognosis of patients with cirrhosis.

Outcome	HR per unit (95% CI)	P value	HR per SD (95% CI)	P value
**30-day mortality**				
RDW^a^	1.12 (1.09 ~ 1.15)	<0.001	1.41 (1.30 ~ 1.52)	<0.001
RAR^b^	1.08 (1.05 ~ 1.11)	<0.001	1.17 (1.11 ~ 1.24)	<0.001
RPR^a^	2.50 (1.70 ~ 3.68)	<0.001	1.19 (1.11 ~ 1.28)	<0.001
HRR^c^	0.03 (0.01 ~ 0.07)	<0.001	0.52 (0.44 ~ 0.61)	<0.001
**90-day mortality**				
RDW^a^	1.14 (1.10 ~ 1.18)	<0.001	1.39 (1.29 ~ 1.49)	<0.001
RAR^b^	1.11 (1.05 ~ 1.17)	<0.001	1.18 (1.12 ~ 1.24)	<0.001
RPR^a^	2.30 (1.29 ~ 4.08)	0.005	1.17 (1.09 ~ 1.25)	<0.001
HRR^c^	0.01 (0.00 ~ 0.03)	<0.001	0.51 (0.44 ~ 0.6)	<0.001
**365-day mortality**				
RDW^a^	1.14 (1.10 ~ 1.18)	<0.001	1.33 (1.25 ~ 1.42)	<0.001
RAR^b^	1.10 (1.05 ~ 1.16)	<0.001	1.18 (1.12 ~ 1.24)	<0.001
RPR^a^	2.23 (1.29 ~ 3.83)	0.004	1.15 (1.08 ~ 1.22)	<0.001
HRR^c^	0.01 (0.00 ~ 0.03)	<0.001	0.56 (0.49 ~ 0.64)	<0.001

Abbreviations: RDW, red cell distribution width; RAR, red cell distribution width-to-albumin ratio; RPR, red cell distribution width-to-platelet ratio; HRR, hemoglobin-to-red cell distribution width; MIMIC-IV, Medical Information Mart for Intensive Care IV;HR: hazard ratio; CI: confidence interval; SD, standard deviation.

a: Models of RDW and RPR were adjusted for sex, age, race, BMI, Charlson Comorbidity Index, vasopressin use, albumin use, mechanical ventilation, renal replacement therapy, hemoglobin, hematocrit, albumin, potassium, lactate; Model for RAR excluded albumin from adjustment; Model for HRR excluded hemoglobin from adjustment.

b: SD values are derived from Table 1. Per-SD HRs were calculated as HR per unit^SD.

c: Unadjusted hazard ratios are provided in Supplementary Table 3 in S1 File.

### 3.4 Assessment of nonlinear relationships between RDW-related biomarkers and mortality

**Fig 2:**
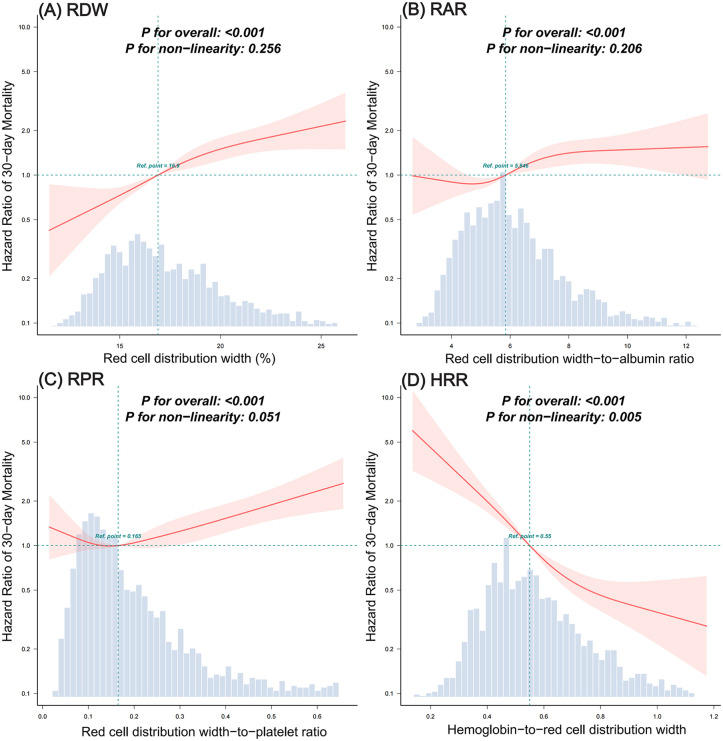
Analysis of the association between RAW-derived indices and 30d-mortality. Data were fitted using Cox proportional hazards regression models based on restricted cubic splines, with each RDW-derived index entered as a continuous variable. Models were adjusted for the same covariates as in the fully adjusted models (Models of RDW and RPR were adjusted for sex, age, race, BMI, Charlson Comorbidity Index, vasopressin use, albumin use, mechanical ventilation, renal replacement therapy, hemoglobin, hematocrit, albumin, potassium, lactate; Model for RAR excluded albumin from adjustment; Model for HRR excluded hemoglobin from adjustment.). The gray shaded areas represent 95% confidence intervals. Abbreviations: RDW, red cell distribution width; RAR, red cell distribution width-to-albumin ratio; RPR, red cell distribution width-to-platelet ratio; HRR, hemoglobin-to-red cell distribution width.

Restricted cubic spline (Fig 2, Supplementary Fig. 4-5 in S1 File) analysis revealed linear associations of RDW and RPR with short-term mortality (30-day mortality), whereas non-linear relationships were observed with long-term mortality (365-day mortality). HRR exhibited significant non-linearity across all endpoints (P = 0.005–0.016), while RAR demonstrated linear relationships across all endpoints (P > 0.05). Threshold analysis identified six significant inflection points (Supplementary Table 4 in S1 File). For HRR, consistent inflection points were observed across all endpoints, ranging from 0.71 to 0.75; below this threshold, each one-unit increase conferred a significant protective association (HR 0.018–0.031, P < 0.001), whereas above the threshold, the protective effect was attenuated and no longer statistically significant (HR 0.079–0.188, P > 0.05). The inflection points for RPR ranged from 0.116 to 0.141; below these values, the association was not statistically significant, but above them, mortality risk increased approximately five-fold (HR 4.98–5.00, P < 0.001). For RDW, the inflection point for 365-day mortality was 17.25; the association was stronger below this threshold (HR 1.236) than above it (HR 1.068), indicating a saturation effect at higher values.

### 3.5 Predictive performances of RDW-related biomarkers

**Fig 3:**
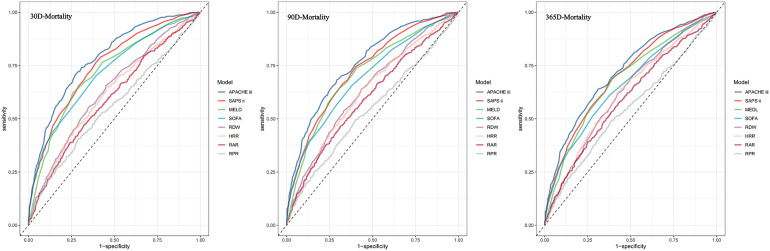
ROC analyses of predictors of RDW, RAR, RPR, and HRR for 30-day, 90-day and 365-day mortality in patients with cirrhosis. **(A)** RDW for 30-day mortality in cirrhosis patients, compared with RAR, RPR, HRR, APS III, SAPS II, MELD, and SOFA scores; (B) RDW for 90-day mortality in cirrhosis patients, compared with RAR, RPR, HRR, APS III, SAPS II, MELD, and SOFA scores; (C) RDW for 365-day mortality in cirrhosis patients, compared with RAR, RPR, HRR, APS III, SAPS II, MELD, and SOFA scores; Abbreviations: RDW, red cell distribution width; RAR, red cell distribution width-to-albumin ratio; RPR, red cell distribution width-to-platelet ratio; HRR, hemoglobin-to-red cell distribution width; MIMIC-IV, Medical Information Mart for Intensive Care IV. APS III, Acute Physiology and Chronic Health Evaluation III; SAPS II: Simplified Acute Physiology Score II; MELD: Model for End-Stage Liver Disease; SOFA: Sequential Organ Failure Assessment.

ROC analysis was used to evaluate the predictive capability of the RDW-related indices. Fig 3 shows the comparison of AUC values between RDW, RAR, RPR, and HRR for 30-day, 90-day, and 365-day mortality rates. Table 3 presents the AUC, sensitivity, and specificity of all ROC curves. The discriminatory ability of the four biomarkers for 30-day mortality showed that RDW had the highest AUC (63.30%, 95% CI 61.46–65.14%), followed by HRR (61.71%, 95% CI 59.85–63.57%), RAR (59.55%, 95% CI 57.66–61.44%), and RPR (55.58%, 95% CI 53.63–57.53%). ROC analyses for 90-day and 365-day mortality revealed a similar pattern. When combined with established severity scores for predicting 30-day mortality, RDW provided incremental predictive value (Table 4 and Supplementary Fig. 6 in S1 File). The combination of APS III with RDW achieved an AUC of 79.03% (vs. 78.35% for APS III alone, increase 0.68%). Similarly, SAPS II + RDW achieved 76.46% (vs. 74.88%, increase 1.58%), MELD + RDW achieved 73.04% (vs. 72.17%, increase 0.87%), and SOFA + RDW achieved 73.27% (vs. 71.66%, increase 1.61%). All these incremental improvements were statistically significant. While these AUC increments were modest, examination of sensitivity and specificity at optimal cut-points found SAPS II combined with RDW improved sensitivity from 76.3% to 81.7%, reducing false-negative rates in a screening context; conversely, SOFA combined with RDW markedly increased specificity from 60.8% to 80.3%, substantially reducing false-positive rates for confirming low-risk status.

**Table 3:** Information of receiver operating characteristic curves.

Variables	AUC (%)	95% CI (%)	Threshold	Specificity	Sensitivity
**30-day mortality**					
RDW	63.30	60.61 ~ 65.99	17.55	0.651	0.553
RAR	59.55	56.76 ~ 62.33	6.44	0.674	0.474
RPR	55.58	52.69 ~ 58.46	0.23	0.710	0.405
HRR	61.71	58.96 ~ 64.45	0.56	0.548	0.644
**90-day mortality**					
RDW	63.66	61.11 ~ 66.21	17.55	0.667	0.538
RAR	59.87	57.23 ~ 62.51	6.44	0.686	0.465
RPR	54.29	51.56 ~ 57.01	0.19	0.602	0.493
HRR	62.43	59.84 ~ 65.01	0.56	0.560	0.644
**365-day mortality**					
RDW	62.48	59.96 ~ 64.99	16.75	0.577	0.617
RAR	59.49	56.92 ~ 62.05	5.86	0.571	0.576
RPR	54.55	51.93 ~ 57.17	0.19	0.615	0.490
HRR	61.82	59.28 ~ 64.35	0.56	0.576	0.624

Abbreviations: ROC, receiver operating characteristic curve; AUC, area under the curve; CI, confidence interval; RDW, red cell distribution width; RAR, red cell distribution width-to-albumin ratio; RPR, red cell distribution width-to-platelet ratio; HRR, hemoglobin-to-red cell distribution width.

**Table 4:** Comparison of Predictive Abilities of APS III, SAPS II, MELD, SOFA Scores and Their Combined Models with RDW.

Variables	AUC (%)	95% CI (%)	Specificity	Sensitivity	P value
**30-day mortality**					
**APS III**	78.35	76.16 ~ 80.53	0.727	0.708	0.041
APS III + RDW	79.03	76.87 ~ 81.20	0.739	0.714	
**SAPS II**	74.88	72.53 ~ 77.23	0.609	0.763	0.001
SAPS II + RDW	76.46	74.18 ~ 78.73	0.569	0.817	
**MELD**	72.17	69.69 ~ 74.65	0.666	0.684	0.015
MELD + RDW	73.04	70.58 ~ 75.50	0.682	0.708	
**SOFA**	71.66	69.14 ~ 74.18	0.608	0.701	<0.001
SOFA + RDW	73.27	70.78 ~ 75.76	0.803	0.551	

Abbreviations: ROC, receiver operating characteristic curve; AUC, area under the curve; CI, confidence interval; RDW, red cell distribution width; RAR, red cell distribution width-to-albumin ratio; RPR, red cell distribution width-to-platelet ratio; HRR, hemoglobin-to-red cell distribution width; APS III, Acute Physiology and Chronic Health Evaluation III; SAPS II: Simplified Acute Physiology Score II; MELD: Model for End-Stage Liver Disease; SOFA: Sequential Organ Failure Assessment.

### 3.6 Sensitivity analysis

The results of the subgroup analyses are presented in Supplementary Figs. 7-9 in S1 File. We found that the associations between different RDW-derived indices and all outcomes remained significant across all subgroups, indicating robustness of the conclusions. However, with the exception of RAR, we also observed a significant interaction between mortality and different BMI strata (P for interaction < 0.05). Uncontrolled confounding factors were assessed by calculating the E value. The E-values of the RDW, RAR, RPR, HRR, as well as the four standardized indices for all mortality outcomes are presented in Supplementary Table 5 in S1 File. These relatively large E-values indicate that all models were robust.

## 4 Discussion

Our large cohort study focused on the prognostic value of RDW and the three additional RDW-derived inflammatory markers (RAR, RPR, and HRR) in ICU patients with cirrhosis, including both short- and long-term mortality. The findings showed a significant association between these biomarkers and mortality in ICU patients with cirrhosis. In the evaluation of 30-day, 90-day, and 365-day mortality, RDW outperformed the other three indicators. For critically ill patients with cirrhosis, it is not recommended to use RDW-related inflammatory markers as standalone prognostic tools. However, RDW may potentially serve as a supplementary instrument to enhance the capacity for risk stratification.

The association between RDW and mortality in ICU patients is a subject of considerable interest. RDW has been identified as a predictor of mortality in various clinical settings, including ICU patients [17]. Numerous studies have aimed to clarify the relationship between RDW and different liver diseases, including chronic viral hepatitis, non-alcoholic fatty liver disease, primary biliary cholangitis, and hepatocellular carcinoma [18–22]. Previous studies have demonstrated a close association between RDW and liver injury and hepatic fibrosis [18,23–25]. Chronic liver injury leads to an increase in pro-inflammatory factors, which in turn affect the production of erythropoietin and the utilization of iron. This ultimately results in alterations to the morphology and apoptosis of RBCs. Additionally, nutritional deficiencies and cachexia may also contribute to anisocytosis and increase RDW [18]. Consequently, RDW may serve as a potential prognostic marker for chronic liver disease. Consistent with the majority of prior research findings, our study also showed that RDW was significantly associated with prognosis in critically ill patients with severe cirrhosis, encompassing both short- and long-term mortality [20,26,27]. Moreover, in our study population, RDW demonstrated superior prognostic discriminative ability compared with other RDW-related indices.

RAR has also been explored as a prognostic indicator. The RAR represents the combined effects of inflammation (denoted by elevated RDW) and impaired hepatic synthetic function (reflected by reduced albumin). In patients with sepsis, higher RAR has been associated with poor clinical outcomes, including increased mortality. This suggests that RAR could serve as a useful marker for assessing the prognosis of critically ill patients [28]. However, there are currently few studies on RAR and liver disease. Mao et al. found that in a cohort of 167 patients diagnosed with hepatitis B virus (HBV)-related compensated cirrhosis, the RAR levels were higher in non-survivors than in survivors. Elevated RAR levels were significantly associated with adverse outcomes, and there was no significant difference in predictive ability between RAR levels and the Model for End-Stage Liver Disease (MELD) score (RAR: AUC = 0.773; MELD: AUC = 0.830) [29]. Tan et al. conducted a retrospective study of 1,413 patients with HBV-related hepatocellular carcinoma (HBV-HCC). They identified that a high RAR was an independent risk factor for long-term overall survival in patients with HBV-HCC. Compared with RDW alone, albumin, total bilirubin, and Child-Pugh score, RAR demonstrated superior prognostic discriminative ability, with an AUC of 0.751. Incorporating RAR into the traditional HCC staging system significantly enhances the ability to predict overall mortality risk [30]. Our study also found that RAR is an independent risk factor for mortality in critically ill patients with cirrhosis. However, its predictive performance did not exceed that of the RDW alone in our study population.

Similarly, higher RPR has been linked to increased mortality, indicating its potential as a prognostic tool in critical care settings [12]. Through the RPR, thrombocytopenia in portal hypertension potentiates the prognostic utility of RDW elevation. In the field of liver diseases, multiple studies have identified RPR as a robust predictor of liver fibrosis and cirrhosis, and it is recommended for use in noninvasive clinical diagnosis [25,31,32]. Dallio et al. found that RPR demonstrated superiority over other non-invasive tools in predicting decompensation events in patients with metabolic-associated steatohepatitis liver disease (MASLD) and can serve as an effective predictive tool [33]. Zhang et al. analyzed the association between RPR and 30-day mortality in 168 patients with HBV-related decompensated cirrhosis. They found that RPR was an independent prognostic predictor, and combining RPR with the MELD score further enhanced its predictive value for mortality [9]. In our study, RPR was significantly associated with both short- and long-term mortality in critically ill patients with cirrhosis. However, its prognostic performance was not superior to that of RDW.

The HRR is another RDW-related index that has been studied in relation to mortality. Although less commonly discussed, HRR may provide additional insights into patient prognosis, particularly in the context of critical illnesses [34–37]. The HRR combines hemoglobin levels and RDW elevation (reflecting inflammatory burden), serving as a critical biomarker in sepsis-associated cirrhosis decompensation. Currently, the only study reporting on the correlation between HRR and the prognosis of liver diseases was by Yu et al. Their research found that a low HRR was significantly associated with the 30-day mortality rate in patients with HBV-related decompensated cirrhosis. Thus, HRR may serve as a promising prognostic predictor for patients with liver diseases [14]. In our cohort of 1,871 ICU cirrhosis patients, a low HRR was significantly associated with 30-day, 90-day, and 365-day mortality. It also demonstrated predictive capabilities comparable to those of RDW and superior to those of RAR and RPR. The prognostic value of HRR in patients with different liver diseases warrants further investigation through additional studies.

RDW-related inflammatory biomarkers are easily obtainable in clinical practice and are straightforward to calculate, potentially offering a predictive value for critically ill patients [19,38–42]. A significant practical advantage of RDW-related biomarkers is their inherent cost-effectiveness. Since RDW, albumin, platelets, and hemoglobin are routinely measured components of standard complete blood count (CBC) and comprehensive metabolic panel (CMP) tests in hospitalized patients, calculating ratios such as RAR, RPR, or HRR incurs no additional laboratory costs. This contrasts sharply with specialized inflammatory markers (e.g., CRP, procalcitonin) or proprietary prognostic scores requiring dedicated assays. The minimal incremental expense supports the feasibility of widespread implementation in resource-constrained settings. These biomarkers could help prioritize high-risk patients for intensive monitoring or early intervention, particularly where advanced scoring systems are unavailable. Different RDW-related inflammatory biomarkers may reflect distinct pathophysiological changes in patients admitted to the ICU.

The sensitivity–specificity trade-offs in Table 3 reveal score-specific rather than uniform clinical utility. When combined with SAPS II, RDW improved sensitivity from 76.3% to 81.7% (+5.4 percentage points), reducing false-negative classifications in early screening. When combined with SOFA, RDW increased specificity from 60.8% to 80.3% (+19.5 percentage points), substantially reducing false-positive rates for resource allocation decisions. These divergent profiles suggest that RDW shifts the operating point of the host score: toward higher sensitivity for triage when added to SAPS II, and toward higher specificity for confirmation when added to SOFA. The modest AUC increments (0.68–1.61%) therefore understate the clinically meaningful optimisation at specific decision thresholds. The clinical utility of this score-specific optimisation requires prospective validation. While the standalone AUCs for RDW-derived biomarkers were modest (≤63.7%), their incremental value when combined with established scores (AUC improvement 0.68–1.6%), combined with their cost-effectiveness and universal availability in routine laboratories, supports their potential role as adjunctive tools — not replacements — for existing prognostic systems. This may reflect RDW’s dual role in capturing both inflammatory status (through erythropoietic suppression and iron dysregulation) and nutritional/hematopoietic dysfunction in cirrhosis, dimensions that are not fully captured by organ-specific severity scores such as SOFA or MELD. The clinical impact of these AUC improvements remains to be established through prospective validation.

Strengths of this study include: (1) a large cohort of 1871 ICU patients with cirrhosis evaluating RDW-derived biomarkers in critical care; (2) the first study to simultaneously examine four commonly used RDW-related inflammatory indices (RDW, RAR, RPR, HRR) for both short-term (30-day) and long-term (365-day) prognoses; (3) application of z-score standardisation to address scaling artifacts inherent to ratio-derived biomarkers, enabling clinically interpretable per-SD hazard ratios and valid cross-biomarker effect-size comparisons — particularly for HRR, whose per-unit HR (0.03) is not clinically meaningful due to its narrow distribution (SD 0.2); (4) use of E-values to quantify robustness to unmeasured confounding; and (5) a head-to-head comparison of the discriminative performance of all four biomarkers across three mortality endpoints, with evaluation of incremental predictive value when combined with established severity scores. We acknowledge that the median values used as exploratory cut-points (e.g., RDW > 16.9%) were chosen for descriptive purposes and are not validated clinical thresholds; their utility for guiding clinical intervention requires prospective evaluation in external cohorts.

This study has several limitations. First, its retrospective design inherently limits causal inference, and prospective validation is required to elevate the evidence level. Second, as a single-center study, our findings may not be generalizable to other healthcare systems or patient populations; multicenter external validation is essential before clinical implementation. Third, our cohort inherently represents the sickest cirrhosis patients requiring critical care, excluding those managed on general wards or in palliative settings. While this limits applicability to non-critical populations, it accurately reflects the intended target population for biomarker prognostication in the ICU. Fourth, we examined only baseline biomarker values measured within the first 24 hours of ICU admission. Dynamic changes during the ICU stay may capture evolving inflammatory status and treatment response beyond what static measurements can offer. Future studies should evaluate longitudinal RDW trajectories and delta-RDW (change from baseline) to determine whether time-varying biomarker values enhance predictive performance. Fifth, the absence of granular treatment-regimen data (e.g., specific vasopressor dosing, antibiotic protocols, transfusion thresholds) and cause-specific mortality information in our dataset precludes deeper mechanistic and outcome analyses. Finally, the distinct pathophysiological mechanisms reflected by each RDW-related biomarker in the context of cirrhosis — whether primarily inflammatory, nutritional, or hematopoietic — remain to be fully elucidated through translational research.

## 5 Conclusion

In conclusion, our data suggest that RDW-related biomarkers are independently associated with 30-day, 90-day, and 365-day mortality in ICU patients with cirrhosis. Among the four indices, RDW and HRR demonstrated the strongest per-SD associations, though standalone discrimination was modest. The integration of these indices into clinical practice could provide modest additional prognostic information when combined with established scoring systems. Further prospective studies, external validation, and investigation of dynamic biomarker changes are required before these findings can inform clinical decision-making.

## Supporting information

S1 File Contains all the supporting tables and figures. (DOCX)

## Abbreviations

ALT: alanine aminotransferase
APS III: acute physiology score III
AST: aspartate aminotransferase
AUC: area under the curve
BMI: body mass index
CI: confidence interval
DBP: diastolic blood pressure
Hb: hemoglobin
HBV: hepatitis B virus
HCC: hepatocellular carcinoma
HR: Hazard ratio
HRR: hemoglobin-to-red cell distribution width ratio
ICU: intensive care unit
INR: international normalized ratio
IQR: interquartile range
MBP: mean arterial pressure
MIMIC-IV: medical information mart for intensive care IV
PT: prothrombin time
RAR: red cell distribution width-to-albumin ratio
RDW: red cell distribution width
ROC: receiver operating characteristic
RPR: red cell distribution width-to-platelet ratio
RRT: renal replacement therapy
SBP: systolic blood pressure
SpO2: pulse oximetry
