# Supplementary material for: Association between Red Cell Distribution Width (RDW)-related inflammatory biomarkers and prognosis in ICU Cirrhosis patients: Evidence from the MIMIC-IV
Source: PLoS One. 2026 Jun 26;21(6):e0352023. doi: 10.1371/journal.pone.0352023 (PMC13308776; doi:10.1371/journal.pone.0352023)
Supplement: S1 File — (DOCX) [file pone.0352023.s001.docx]

**S1 Table. The basic demographics and clinical characteristics in patients with cirrhosis.**

| **Characteristics** | **Total cohort**  **(n = 3810)** | **Excluded**  **(n = 1939)** | **Included**  **(n = 1871)** | **P-value** |
| --- | --- | --- | --- | --- |
| **Demographics** |  |  |  |  |
| Male, n (%) | 2484 (65.2) | 1272 (65.6) | 1212 (64.8) | 0.594 |
| Age, years | 60.0 ± 12.5 | 61.2 ± 12.6 | 58.8 ± 12.3 | < 0.001 |
| Race (white), n (%) | 2498 (76.3) | 1313 (77.5) | 1185 (75.0) | 0.098 |
| BMI (kg/m^2^) | 29.5 ± 7.6 | 29.4 ± 7.4 | 29.6 ± 7.8 | 0.530 |
| **Vital signs** |  |  |  |  |
| Heart rate (beats/min) | 87.4 ± 16.4 | 86.3 ± 16.5 | 88.5 ± 16.3 | < 0.001 |
| SBP (mmHg) | 113.8 ± 16.6 | 114.2 ± 17.3 | 113.3 ± 16.0 | 0.106 |
| DBP (mmHg) | 62.2 ± 11.0 | 62.9 ± 11.6 | 61.4 ± 10.2 | < 0.001 |
| MBP (mmHg) | 76.4 ± 11.3 | 76.6 ± 11.9 | 76.1 ± 10.7 | 0.155 |
| Respiratory rate (beats/min) | 18.9 ± 4.2 | 18.8 ± 4.1 | 19.0 ± 4.2 | 0.036 |
| Temperature (℃) | 36.8 ± 0.5 | 36.7 ± 0.6 | 36.8 ± 0.5 | < 0.001 |
| Spo2 (%) | 96.6 ± 2.8 | 96.4 ± 3.3 | 96.9 ± 2.2 | < 0.001 |
| **Comorbidities, n(%)** |  |  |  |  |
| Hypertension | 1880 (49.3) | 1003 (51.7) | 877 (46.9) | 0.003 |
| Diabetes | 1151 (30.2) | 619 (31.9) | 532 (28.4) | 0.019 |
| Myocardial infarct | 308 ( 8.1) | 175 (9.0) | 133 (7.1) | 0.030 |
| Congestive heart failure | 671 (17.6) | 374 (19.3) | 297 (15.9) | 0.006 |
| Cerebrovascular disease | 267 ( 7.0) | 139 (7.2) | 128 (6.8) | 0.692 |
| Chronic pulmonary disease | 826 (21.7) | 443 (22.8) | 383 (20.5) | 0.075 |
| Renal disease | 769 (20.2) | 409 (21.1) | 360 (19.2) | 0.154 |
| Malignant cancer | 637 (16.7) | 298 (15.4) | 339 (18.1) | 0.023 |
| Peptic ulcer disease | 281 ( 7.4) | 130 (6.7) | 151 (8.1) | 0.107 |
| Malnutrition | 807 (21.2) | 316 (16.3) | 491 (26.2) | < 0.001 |
| **Clinical assessment and treatments, n (%)** |  |  |  |  |
| APS III | 62.7 ± 29.3 | 56.8 ± 28.1 | 68.9 ± 29.4 | < 0.001 |
| Charlson Comorbidity Index | 6.9 ± 2.8 | 6.9 ± 2.9 | 6.9 ± 2.7 | 0.369 |
| Etiology, alcohol cirrhosis | 2011 (52.8) | 985 (50.8) | 1026 (54.8) | 0.013 |
| Albumin use | 2156 (56.9) | 903 (47.0) | 1253 (67.1) | < 0.001 |
| Mechanical ventilation | 1756 (46.1) | 671 (34.6) | 1085 (58.0) | < 0.001 |
| Vasopressin | 1373 (36.0) | 583 (30.1) | 790 (42.2) | < 0.001 |
| RRT | 448 (11.8) | 122 (6.3) | 326 (17.4) | < 0.001 |
| **Laboratory Indicators** |  |  |  |  |
| RDW (%) | 17.1 ± 2.9 | 16.8 ± 2.8 | 17.4 ± 3.0 | < 0.001 |
| RAR | 6.2 ± 2.2 | 6.1 ± 2.3 | 6.2 ± 2.1 | 0.513 |
| RPR | 0.2 (0.1, 0.3) | 0.2 (0.1, 0.2) | 0.2 (0.1, 0.3) | < 0.001 |
| HRR | 0.6 ± 0.2 | 0.6 ± 0.2 | 0.6 ± 0.2 | < 0.001 |
| White blood cells (10^9^/L) | 9.2 (5.9, 14.3) | 8.5 (5.5, 12.9) | 10.1 (6.4, 15.6) | < 0.001 |
| Hemoglobin (g/dL) | 9.7 ± 2.2 | 9.7 ± 2.2 | 9.6 ± 2.1 | 0.055 |
| Platelet count (10^9^/L) | 104.0 (67.0, 157.2) | 107.0 (71.0, 162.0) | 100.0 (65.5, 151.5) | < 0.001 |
| Total bilirubin (mg/dL) | 3.0 (1.3, 7.7) | 2.7 (1.2, 6.5) | 3.3 (1.5, 8.5) | < 0.001 |
| Asparate aminotransferase (U/L) | 74.0 (41.0, 165.0) | 68.0 (40.0, 134.0) | 78.0 (43.0, 212.0) | < 0.001 |
| Alanine aminotransferase (U/L) | 36.0 (21.0, 77.0) | 34.0 (20.0, 63.0) | 37.0 (21.0, 96.5) | < 0.001 |
| Albumin (g/dL) | 3.0 ± 0.7 | 3.0 ± 0.7 | 3.0 ± 0.7 | 0.861 |
| Creatinine (mg/dL) | 1.1 (0.8, 2.0) | 1.1 (0.7, 1.8) | 1.2 (0.8, 2.2) | < 0.001 |
| Urea nitrogen (mg/dL) | 24.0 (15.0, 43.0) | 22.0 (14.0, 39.0) | 27.0 (16.0, 49.0) | < 0.001 |
| Glucose (mmol/L) | 143.5 ± 80.2 | 136.0 ± 69.1 | 150.9 ± 89.4 | < 0.001 |
| Sodium (mmol/L) | 136.3 ± 6.6 | 136.2 ± 6.4 | 136.4 ± 6.8 | 0.480 |
| Potassium (mmol/L) | 4.3 ± 0.9 | 4.3 ± 0.9 | 4.3 ± 0.9 | 0.834 |
| Chloride (mmol/L) | 102.2 ± 7.7 | 102.4 ± 7.5 | 102.0 ± 7.8 | 0.088 |
| Calcium (mmol/L) | 8.3 ± 1.0 | 8.2 ± 0.9 | 8.4 ± 1.1 | < 0.001 |
| Anion gap (mmol/L) | 16.5 ± 6.2 | 16.4 ± 6.3 | 16.5 ± 6.2 | 0.545 |
| Lactate (mmol/L) | 2.5 (1.7, 4.2) | 2.3 (1.6, 4.1) | 2.6 (1.7, 4.3) | 0.016 |
| **Outcome**, n (%) |  |  |  |  |
| 30-day mortality | 1101 (28.9) | 506 (26.1) | 595 (31.8) | < 0.001 |
| 90-day mortality | 1400 (36.7) | 664 (34.2) | 736 (39.3) | 0.001 |
| 365-day mortality | 1757 (46.1) | 859 (44.3) | 898 (48.0) | 0.022 |
| Variceal bleeding | 436 (11.4) | 215 (11.1) | 221 (11.8) | 0.483 |
| Ascites | 1762 (46.2) | 790 (40.7) | 972 (52.0) | < 0.001 |
| Hepatorenal syndrome | 501 (13.1) | 179 (9.2) | 322 (17.2) | < 0.001 |
| Hepatic encephalopathy | 353 ( 9.3) | 163 (8.4) | 190 (10.2) | 0.063 |
| Spontaneous peritonitis | 348 ( 9.1) | 128 (6.6) | 220 (11.8) | < 0.001 |

Abbreviations: BMI, body mass index; SBP, systolic blood pressure; DBP, diastolic blood pressure; MBP, mean arterial pressure; SpO_2_, pulse oximetry; APS III, Acute Physiology Score III; RRT, renal replacement therapy; RDW, red cell distribution width; RAR, red cell distribution width-to-albumin ratio; RPR, red cell distribution width-to-platelet ratio; HRR, hemoglobin-to-red cell distribution width.

**S2 Table. Missingness Status of Covariates**

| Variable | Miss frequence | Miss percentage(%) |
| --- | --- | --- |
| Gender | 0 | 0 |
| Age | 0 | 0 |
| Race | 291 | 15.55 |
| BMI | 707 | 37.79 |
| Charlson Comorbidity Index | 0 | 0 |
| RRT | 0 | 0 |
| Albumin use | 4 | 0.21 |
| Mechanical ventilation | 0 | 0 |
| Vasopressin | 0 | 0 |
| Albumin | 0 | 0 |
| Hematocrit | 4 | 0.21 |
| Hemoglobin | 0 | 0 |
| Lactate | 485 | 25.92 |
| Potassium | 2 | 0.11 |

Abbreviations: BMI, body mass index; SBP, systolic blood pressure; DBP, diastolic blood pressure; MAP, mean arterial pressure; SpO_2_, pulse oximetry; APSIII, Acute Physiology Score III; RRT, renal replacement therapy; RDW, red cell distribution width; RAR, red cell distribution width-to-albumin ratio; RPR, red cell distribution width-to-platelet ratio; HRR, hemoglobin-to-red cell distribution width.

**S3 Table. Univariable Analysis for Distinct Mortality Outcomes**

| **Characteristics** | **30-day mortality** | | **90-day mortality** | | **365-day mortality** | |
| --- | --- | --- | --- | --- | --- | --- |
|  | **HR (95% CI)** | **P value** | **HR (95% CI)** | **P value** | **HR (95% CI)** | **P value** |
| **Demographics** |  |  |  |  |  |  |
| Male, n (%) | 0.91 (0.77,1.08) | 0.273 | 0.97 (0.83,1.13) | 0.7 | 0.98 (0.86,1.13) | 0.801 |
| Age, years | 1.02 (1.01,1.03) | < 0.001 | 1.02 (1.01,1.02) | < 0.001 | 1.02 (1.01,1.02) | < 0.001 |
| Race (white), n (%) | 0.75 (0.60,0.94) | 0.012 | 0.73 (0.6,0.9) | 0.003 | 0.75 (0.62,0.89) | 0.002 |
| BMI (kg/m^2^) | 1.03 (1.02,1.04) | < 0.001 | 1.02 (1.01,1.04) | < 0.001 | 1.02 (1.01,1.03) | < 0.001 |
| **Vital signs** |  |  |  |  |  |  |
| Heart rate (beats/min) | 1.01 (1.01,1.02) | < 0.001 | 1.01 (1.01,1.02) | < 0.001 | 1.01 (1.00,1.01) | < 0.001 |
| SBP (mmHg) | 0.97 (0.97,0.98) | < 0.001 | 0.97 (0.97,0.98) | < 0.001 | 0.98 (0.97,0.98) | < 0.001 |
| DBP (mmHg) | 0.96 (0.95,0.97) | < 0.001 | 0.97 (0.96,0.97) | < 0.001 | 0.97 (0.96,0.98) | < 0.001 |
| MBP (mmHg) | 0.95 (0.94,0.96) | < 0.001 | 0.96 (0.95,0.96) | < 0.001 | 0.96 (0.95,0.97) | < 0.001 |
| Respiratory rate (beats/min) | 1.08 (1.06,1.10) | < 0.001 | 1.07 (1.05,1.09) | < 0.001 | 1.06 (1.04,1.08) | < 0.001 |
| Temperature (℃) | 0.56 (0.51,0.62) | < 0.001 | 0.57 (0.52,0.62) | < 0.001 | 0.58 (0.54,0.63) | < 0.001 |
| Spo2 (%) | 0.88 (0.85,0.91) | < 0.001 | 0.89 (0.86,0.92) | < 0.001 | 0.90 (0.87,0.92) | < 0.001 |
| **Comorbidities, n(%)** |  |  |  |  |  |  |
| Hypertension | 0.99 (0.84,1.16) | 0.869 | 0.97 (0.84,1.12) | 0.674 | 1.00 (0.88,1.14) | 0.958 |
| Diabetes | 0.91 (0.76,1.09) | 0.315 | 0.93 (0.79,1.09) | 0.353 | 0.98 (0.85,1.13) | 0.767 |
| Myocardial infarct | 1.30 (0.97,1.74) | 0.076 | 1.34 (1.03,1.73) | 0.027 | 1.34 (1.06,1.70) | 0.014 |
| Congestive heart failure | 1.24 (1.01,1.53) | 0.042 | 1.39 (1.16,1.67) | < 0.001 | 1.41 (1.19,1.66) | < 0.001 |
| Cerebrovascular disease | 1.36 (1.02,1.81) | 0.036 | 1.51 (1.17,1.93) | 0.001 | 1.45 (1.15,1.83) | 0.002 |
| Chronic pulmonary disease | 1.08 (0.89,1.31) | 0.444 | 1.05 (0.88,1.26) | 0.559 | 1.16 (0.99,1.35) | 0.066 |
| Renal disease | 1.26 (1.04,1.52) | 0.019 | 1.28 (1.08,1.52) | 0.005 | 1.40 (1.20,1.63) | < 0.001 |
| Malignant cancer | 1.13 (0.92,1.38) | 0.232 | 1.11 (0.92,1.33) | 0.266 | 1.08 (0.91,1.27) | 0.376 |
| Peptic ulcer disease | 0.81 (0.59,1.11) | 0.185 | 0.90 (0.68,1.18) | 0.443 | 0.96 (0.75,1.22) | 0.72 |
| Malnutrition | 0.93 (0.77,1.11) | 0.414 | 1.03 (0.88,1.21) | 0.702 | 1.07 (0.92,1.23) | 0.389 |
| **Clinical assessment and treatments, n (%)** |  |  |  |  |  |  |
| APS III | 1.03 (1.03,1.03) | < 0.001 | 1.03 (1.02,1.03) | < 0.001 | 1.02 (1.02,1.03) | < 0.001 |
| Charlson Comorbidity Index | 1.08 (1.05,1.11) | < 0.001 | 1.09 (1.06,1.12) | < 0.001 | 1.10 (1.08,1.12) | < 0.001 |
| Etiology, alcohol cirrhosis | 1.15 (0.98,1.35) | 0.097 | 1.11 (0.96,1.28) | 0.163 | 1.06 (0.93,1.21) | 0.403 |
| Albumin use | 2.06 (1.69,2.51) | < 0.001 | 1.98 (1.66,2.36) | < 0.001 | 1.77 (1.52,2.06) | < 0.001 |
| Mechanical ventilation | 1.66 (1.40,1.97) | < 0.001 | 1.4 (1.21,1.63) | < 0.001 | 1.18 (1.03,1.34) | 0.017 |
| Vasopressin | 2.56 (2.17,3.02) | < 0.001 | 2.33 (2.01,2.70) | < 0.001 | 2.10 (1.84,2.39) | < 0.001 |
| RRT | 2.45 (2.06,2.93) | < 0.001 | 2.26 (1.92,2.66) | < 0.001 | 2.10 (1.80,2.44) | < 0.001 |
| **Laboratory Indicators** |  |  |  |  |  |  |
| RDW (%) | 1.13 (1.10,1.16) | < 0.001 | 1.13 (1.10,1.15) | < 0.001 | 1.11 (1.09,1.13) | < 0.001 |
| RAR | 1.10 (1.07,1.12) | < 0.001 | 1.1 (1.08,1.12) | < 0.001 | 1.1 (1.08,1.12) | < 0.001 |
| RPR | 2.68 (1.92,3.76) | < 0.001 | 2.43 (1.77,3.34) | < 0.001 | 2.24 (1.66,3.03) | < 0.001 |
| HRR | 0.15 (0.10,0.25) | < 0.001 | 0.15 (0.10,0.23) | < 0.001 | 0.18 (0.12,0.26) | < 0.001 |
| White blood cells (10^9^/L) | 1.04 (1.03,1.05) | < 0.001 | 1.04 (1.03,1.04) | < 0.001 | 1.03 (1.02,1.04) | < 0.001 |
| Hemoglobin (g/dL) | 0.92 (0.88,0.95) | < 0.001 | 0.91 (0.88,0.94) | < 0.001 | 0.91 (0.88,0.94) | < 0.001 |
| Platelet count (10^9^/L) | 1.00 (0.9989,1.001) | 0.941 | 1.0003 (0.9994,1.0012) | 0.492 | 1.0001 (0.9993,1.001) | 0.737 |
| Total bilirubin (mg/dL) | 1.04 (1.03,1.05) | < 0.001 | 1.04 (1.03,1.04) | < 0.001 | 1.03 (1.03,1.04) | < 0.001 |
| Asparate aminotransferase (U/L) | 1.00 (0.9999,1.00) | 0.455 | 0.9999 (0.9998,1) | 0.076 | 0.9998 (0.9997,0.9999) | 0.001 |
| Alanine aminotransferase (U/L) | 0.9995 (0.9992,0.9998) | < 0.001 | 0.9994 (0.9991,0.9996) | < 0.001 | 0.9992 (0.9989,0.9995) | < 0.001 |
| Albumin (g/dL) | 0.96 (0.84,1.08) | 0.473 | 0.94 (0.84,1.06) | 0.324 | 0.93 (0.84,1.04) | 0.206 |
| Creatinine (mg/dL) | 1.13 (1.10,1.17) | < 0.001 | 1.12 (1.09,1.15) | < 0.001 | 1.12 (1.09,1.15) | < 0.001 |
| Urea nitrogen (mg/dL) | 1.01 (1.01,1.01) | < 0.001 | 1.01 (1.01,1.01) | < 0.001 | 1.01 (1.01,1.01) | < 0.001 |
| Glucose (mmol/L) | 0.9961 (0.9947,0.9975) | < 0.001 | 0.9961 (0.9949,0.9973) | < 0.001 | 0.9956 (0.9945,0.9968) | < 0.001 |
| Sodium (mmol/L) | 0.98 (0.96,0.99) | < 0.001 | 0.97 (0.96,0.98) | < 0.001 | 0.97 (0.96,0.98) | < 0.001 |
| Potassium (mmol/L) | 1.20 (1.10,1.31) | < 0.001 | 1.17 (1.08,1.27) | < 0.001 | 1.16 (1.08,1.25) | < 0.001 |
| Chloride (mmol/L) | 0.96 (0.95,0.97) | < 0.001 | 0.96 (0.96,0.97) | < 0.001 | 0.97 (0.96,0.98) | < 0.001 |
| Calcium (mmol/L) | 1.002 (0.9334,1.0755) | 0.957 | 0.97 (0.91,1.04) | 0.356 | 0.97 (0.91,1.03) | 0.264 |
| Anion gap (mmol/L) | 1.07 (1.06,1.09) | < 0.001 | 1.07 (1.05,1.08) | < 0.001 | 1.06 (1.05,1.07) | < 0.001 |
| Lactate (mmol/L) | 1.13 (1.11,1.16) | < 0.001 | 1.13 (1.11,1.15) | < 0.001 | 1.12 (1.10,1.14) | < 0.001 |

Abbreviations: BMI, body mass index; SBP, systolic blood pressure; DBP, diastolic blood pressure; MBP, mean arterial pressure; SpO_2_, pulse oximetry; APS III, Acute Physiology Score III; RRT, renal replacement therapy; RDW, red cell distribution width; RAR, red cell distribution width-to-albumin ratio; RPR, red cell distribution width-to-platelet ratio; HRR, hemoglobin-to-red cell distribution width.

**S4 Table. Saturation Effects in the Relationship Between RDW, RPR, HRR, and Mortality**

| **Variables** | **Adjusted Model** | |
| --- | --- | --- |
|  | **HR (95% CI)** | ***p*-value** |
| **30-day mortality** |  |  |
| **HRR** |  |  |
| < 0.71 | 0.018 (0.005,0.056) | < 0.001 |
| ≥ 0.71 | 0.188 (0.013,2.664) | 0.217 |
| Log-likelihood ratio test | 0.011 | |
| **90-day mortality** |  |  |
| **RPR** |  |  |
| < 0.14 | 0.016 (0,1.343) | 0.067 |
| ≥ 0.14 | 4.983 (2.362,10.513) | < 0.001 |
| Log-likelihood ratio test | 0.012 | |
| **HRR** |  |  |
| < 0.73 | 0.018 (0.006,0.050) | < 0.001 |
| ≥ 0.73 | 0.079 (0.006,1.084) | 0.058 |
| Log-likelihood ratio test | 0.031 | |
| **365-day mortality** |  |  |
| **RDW** |  |  |
| < 17.25 | 1.236 (1.131,1.351) | < 0.001 |
| ≥ 17.25 | 1.068 (1.021,1.118) | 0.005 |
| Log-likelihood ratio test | 0.006 | |
| **RPR** |  |  |
| < 0.12 | 0.003 (0.000,1.262) | 0.060 |
| ≥ 0.12 | 5.001 (2.692,9.290) | < 0.001 |
| Log-likelihood ratio test | 0.021 | |
| **HRR** |  |  |
| < 0.75 | 0.031 (0.013,0.074) | < 0.001 |
| ≥ 0.75 | 0.132 (0.010,1.735) | 0.123 |
| Log-likelihood ratio test | 0.058 | |

Abbreviations: RAR, red cell distribution width-to-albumin ratio; HRR, hemoglobin-to-red cell distribution width; HR, Hazard Ratio; CI, Confidence Interval;

**S5 Table. The E-value for different outcomes**

| **Outcome** | **RR (per unit)** | **E-value** | **Lower CI limit** | **RR (per SD)** | **E-value** | **Lower CI limit** |
| --- | --- | --- | --- | --- | --- | --- |
| **30d-mortality** |  |  |  |  |  |  |
| RDW | 1.082 | 1.379 | 1.317 | 1.269 | 1.852 | 1.688 |
| RAR | 1.055 | 1.300 | 1.223 | 1.115 | 1.473 | 1.359 |
| RPR | 1.876 | 3.158 | 2.242 | 1.128 | 1.508 | 1.359 |
| HRR | 8.678 | 16.840 | 10.542 | 1.570 | 2.516 | 2.164 |
| **90d-mortality** |  |  |  |  |  |  |
| RDW | 1.095 | 1.418 | 1.338 | 1.256 | 1.823 | 1.673 |
| RAR | 1.075 | 1.359 | 1.223 | 1.122 | 1.491 | 1.379 |
| RPR | 1.773 | 2.944 | 1.673 | 1.115 | 1.473 | 1.317 |
| HRR | 17.744 | 34.980 | 17.929 | 1.591 | 2.560 | 2.200 |
| **365d-mortality** |  |  |  |  |  |  |
| RDW | 1.095 | 1.418 | 1.338 | 1.218 | 1.734 | 1.609 |
| RAR | 1.068 | 1.338 | 1.223 | 1.122 | 1.491 | 1.379 |
| RPR | 1.736 | 2.867 | 1.673 | 1.102 | 1.436 | 1.295 |
| HRR | 15.875 | 31.241 | 17.019 | 1.492 | 2.350 | 2.063 |

Abbreviations: RR, relative risk; CI, confidence interval; RDW, red cell distribution width; RAR, red cell distribution width-to-albumin ratio; RPR, red cell distribution width-to-platelet ratio; HRR, hemoglobin-to-red cell distribution width.

**S1 Fig. Kaplan-Meier survival curves for 30d-mortality by RDW, RAR, RPR, and HRR levels in patients with cirrhosis from MIMIC-IV database.**

Abbreviations: RDW, red cell distribution width; RAR, red cell distribution width-to-albumin ratio; RPR, red cell distribution width-to-platelet ratio; HRR, hemoglobin-to-red cell distribution width; MIMIC-IV, Medical Information Mart for Intensive Care IV.

**
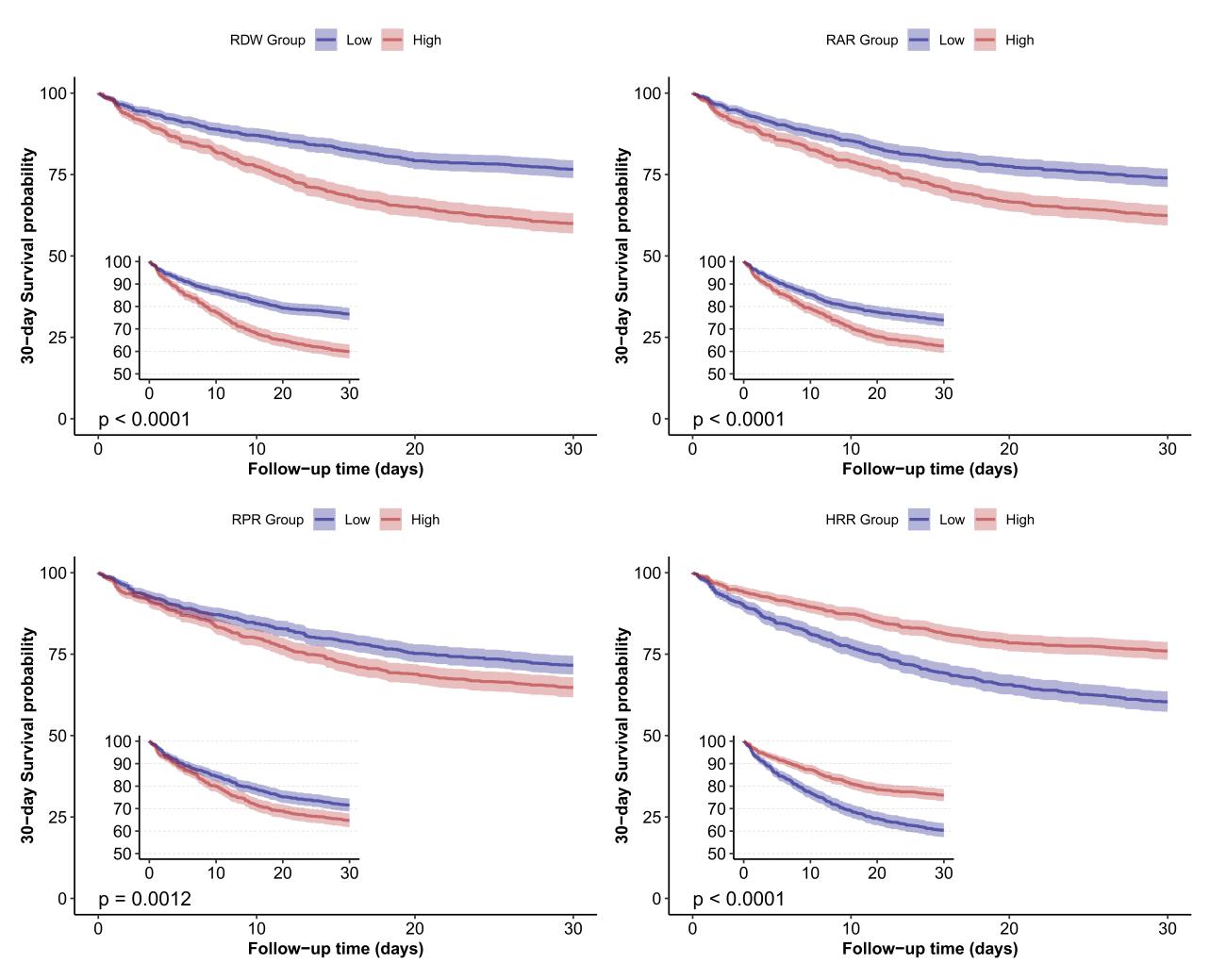
**

**S2 Fig. Kaplan-Meier survival curves for 90d-mortality by RDW, RAR, RPR, and HRR levels in patients with cirrhosis from MIMIC-IV database.**

Abbreviations: RDW, red cell distribution width; RAR, red cell distribution width-to-albumin ratio; RPR, red cell distribution width-to-platelet ratio; HRR, hemoglobin-to-red cell distribution width; MIMIC-IV, Medical Information Mart for Intensive Care IV.


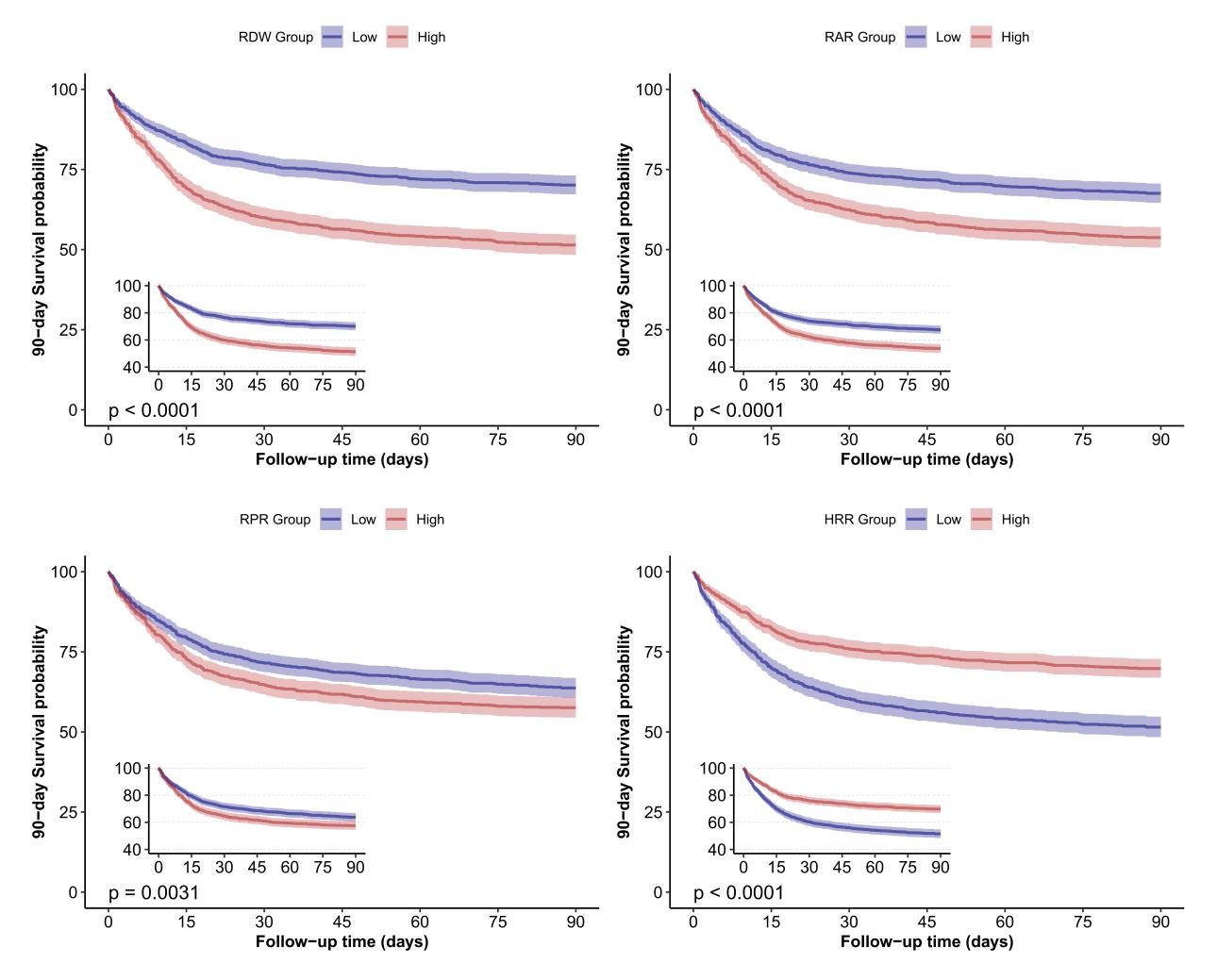


**S3 Fig. Kaplan-Meier survival curves for 365d-mortality by RDW, RAR, RPR, and HRR levels in patients with cirrhosis from MIMIC-IV database.**

Abbreviations: RDW, red cell distribution width; RAR, red cell distribution width-to-albumin ratio; RPR, red cell distribution width-to-platelet ratio; HRR, hemoglobin-to-red cell distribution width; MIMIC-IV, Medical Information Mart for Intensive Care IV.


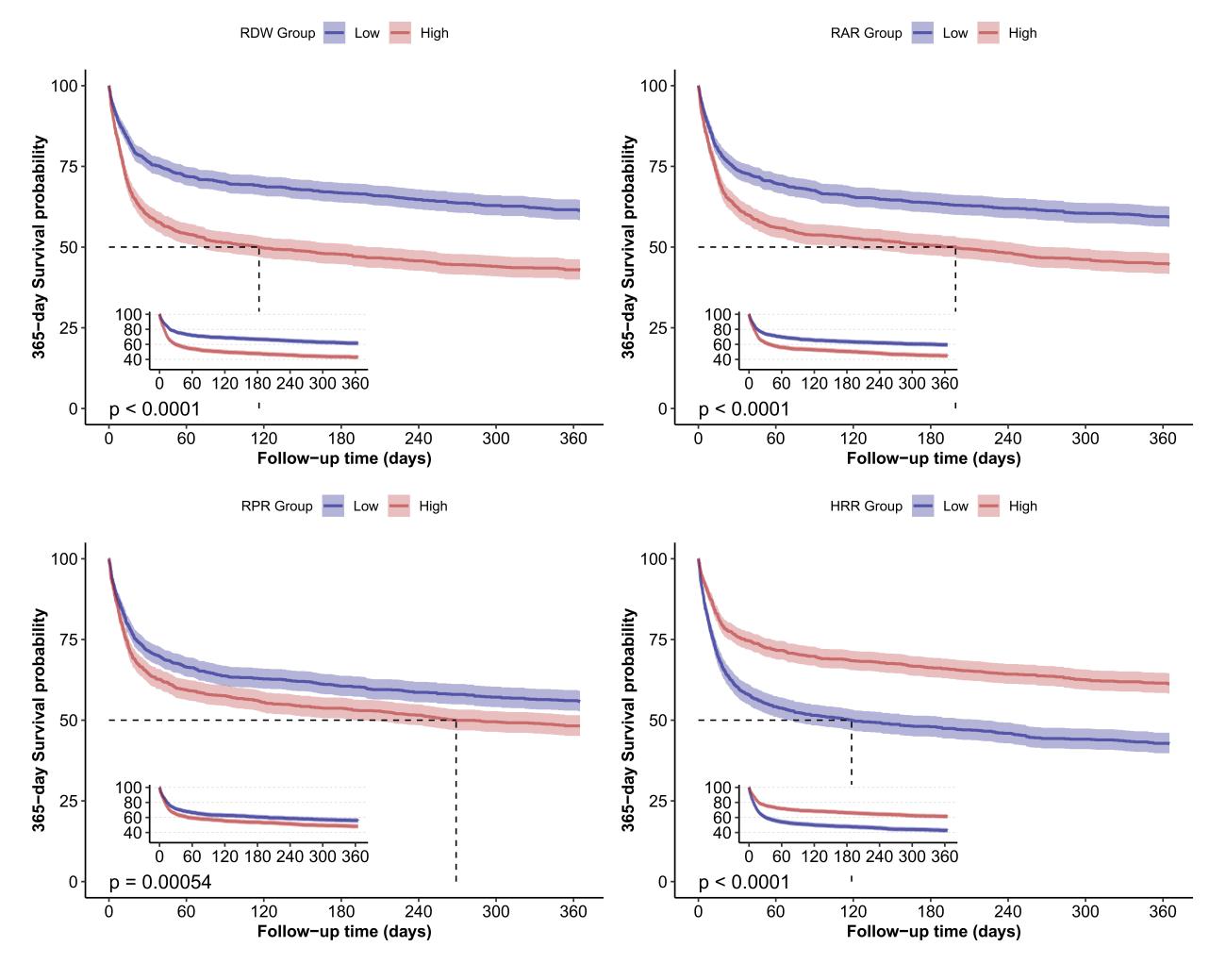


**S4 Fig. Analysis of the association between RAW-derived indices and 90d-mortality.**


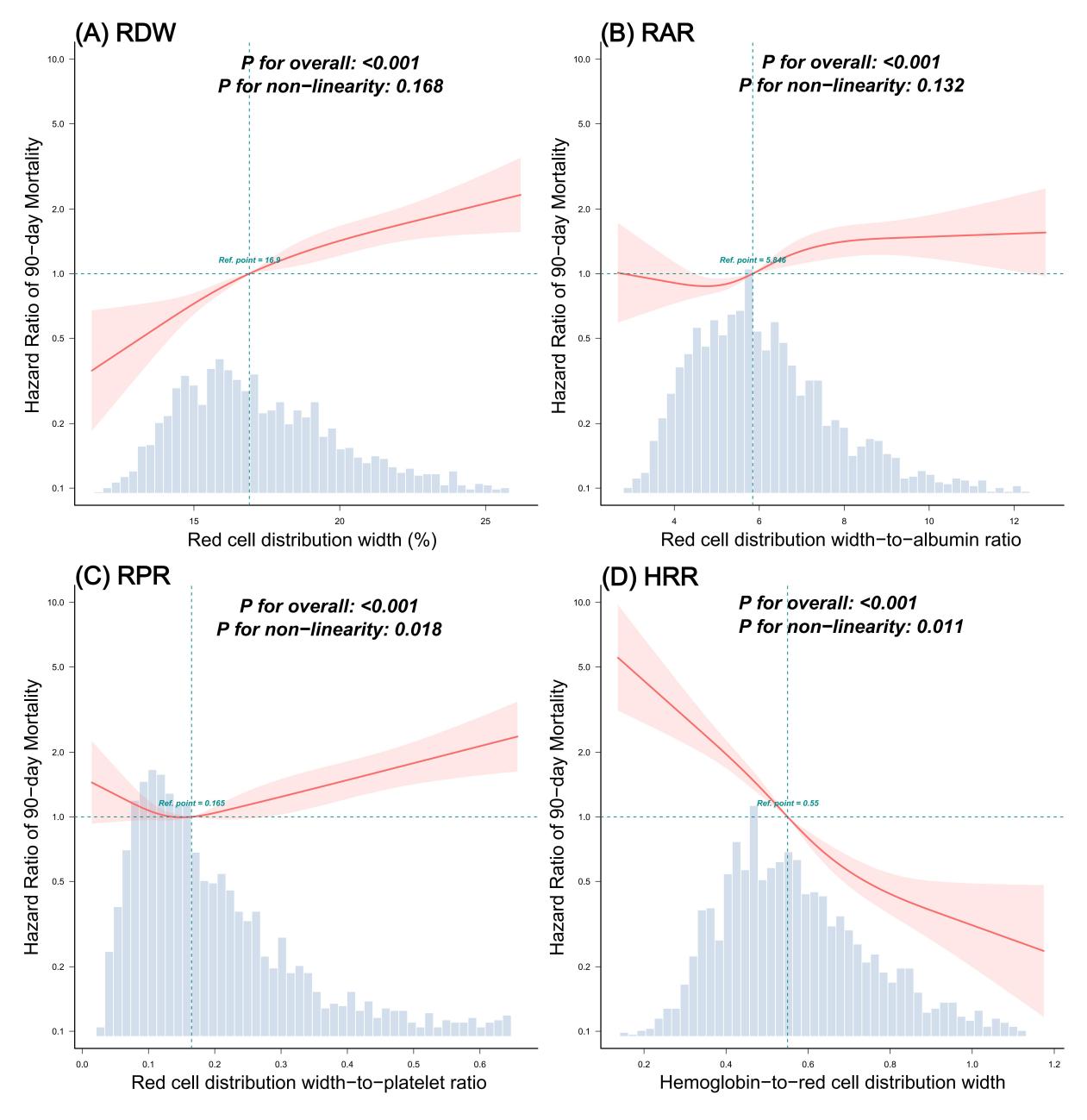


Data were fitted using Cox proportional hazards regression models based on restricted cubic splines, with each RDW-derived index entered as a continuous variable. Models were adjusted for the same covariates as in the fully adjusted models (Models of RDW and RPR were adjusted for sex, age, race, BMI, Charlson Comorbidity Index, vasopressin use, albumin use, mechanical ventilation, renal replacement therapy, hemoglobin, hematocrit, albumin, potassium, lactate; Model for RAR excluded albumin from adjustment; Model for HRR excluded hemoglobin from adjustment). The gray shaded areas represent 95% confidence intervals.

Abbreviations: RDW, red cell distribution width; RAR, red cell distribution width-to-albumin ratio; RPR, red cell distribution width-to-platelet ratio; HRR, hemoglobin-to-red cell distribution width.

**S5 Fig. Analysis of the association between RAW-derived indices and 365d-mortality.**


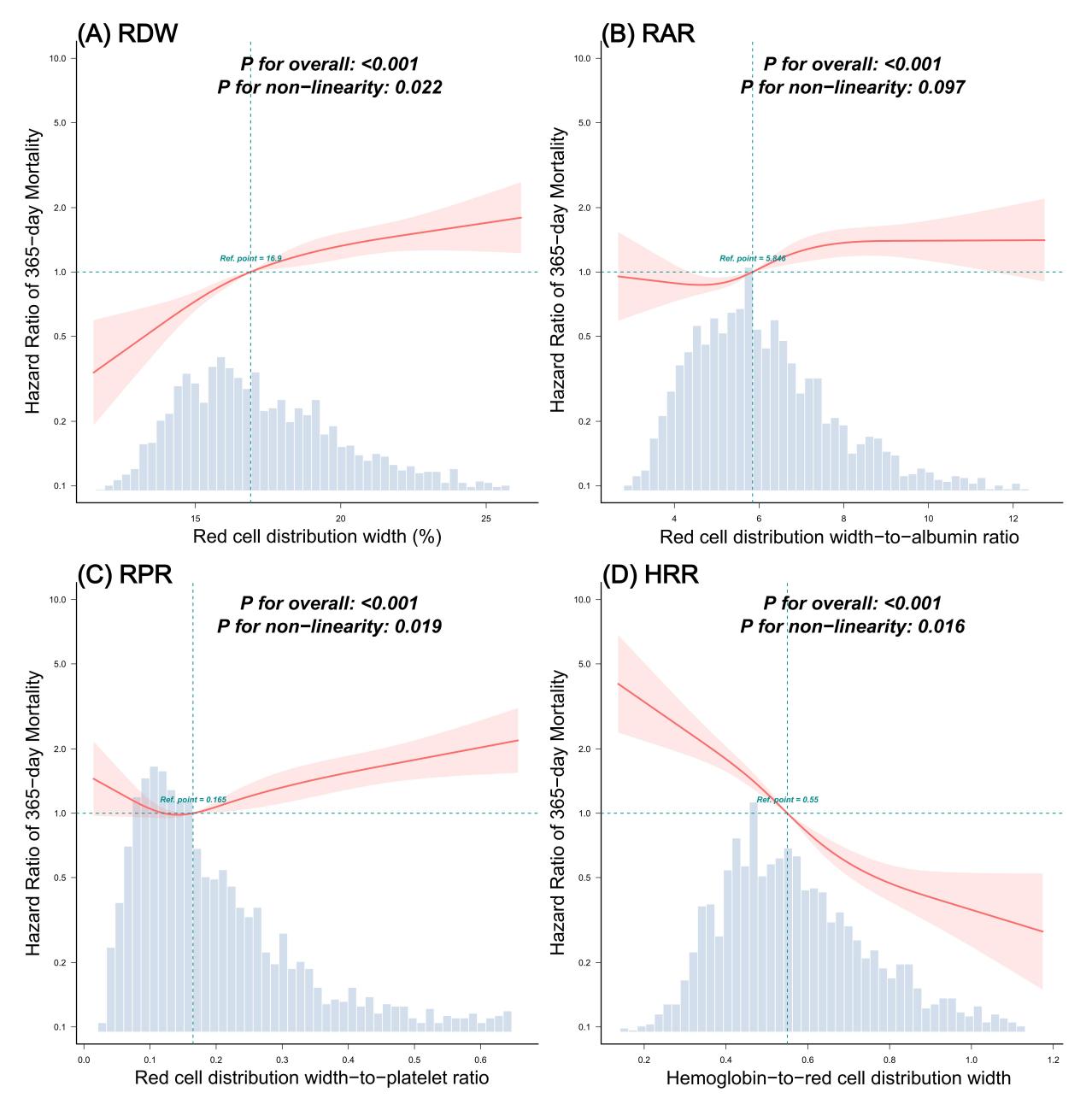


Data were fitted using Cox proportional hazards regression models based on restricted cubic splines, with each RDW-derived index entered as a continuous variable. Models were adjusted for the same covariates as in the fully adjusted models (Models of RDW and RPR were adjusted for sex, age, race, BMI, Charlson Comorbidity Index, vasopressin use, albumin use, mechanical ventilation, renal replacement therapy, hemoglobin, hematocrit, albumin, potassium, lactate; Model for RAR excluded albumin from adjustment; Model for HRR excluded hemoglobin from adjustment.). The gray shaded areas represent 95% confidence intervals.

Abbreviations: RDW, red cell distribution width; RAR, red cell distribution width-to-albumin ratio; RPR, red cell distribution width-to-platelet ratio; HRR, hemoglobin-to-red cell distribution width.

**S6 Fig. P**redictive value of RDW when combined with existing prognostic scoring systems for 30-day mortality.****

****
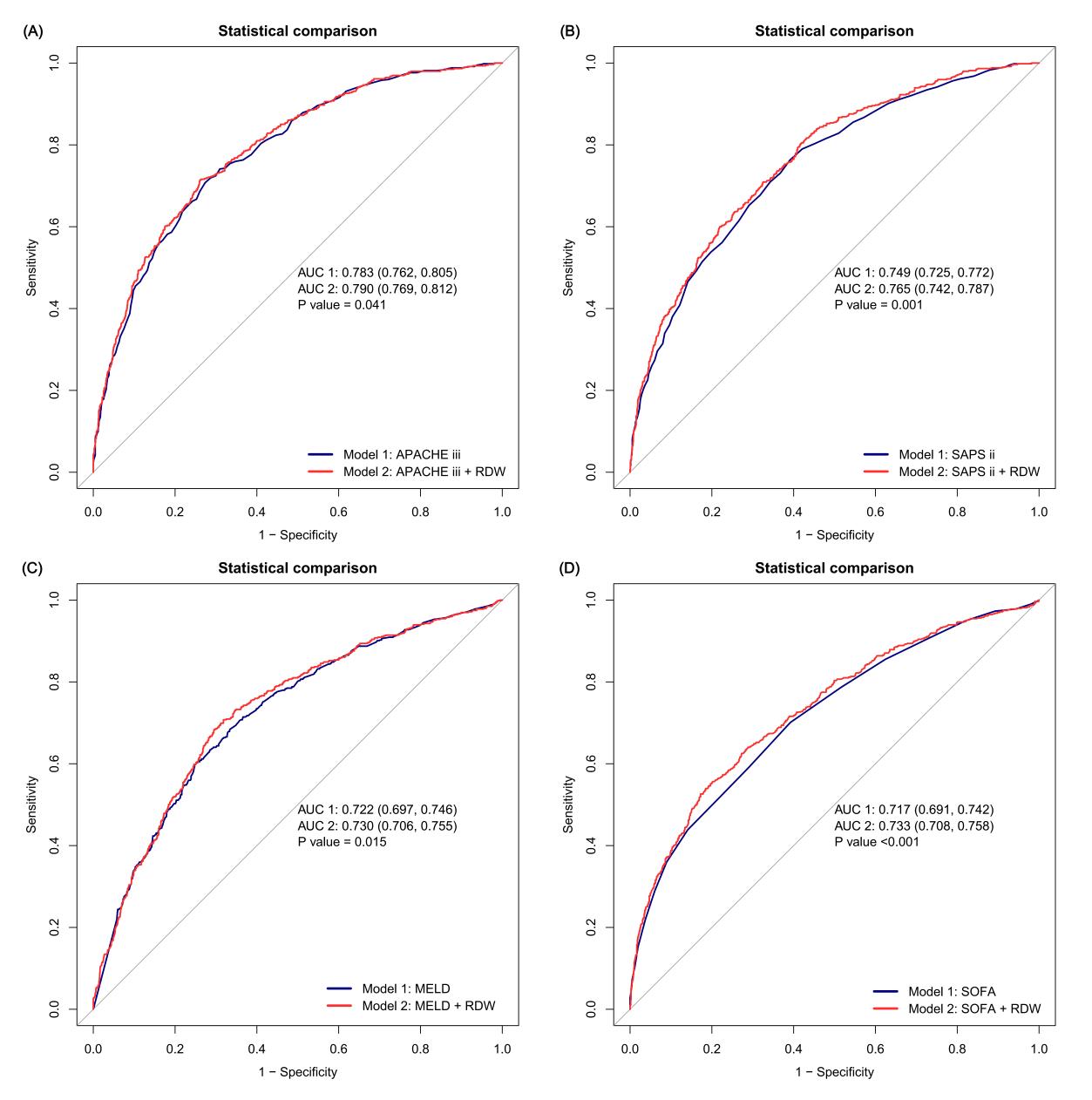
****

ROC curves comparing the predictive performance of (A) APACHE III, (B) SAPS II, (C) MELD, and (D) SOFA scores alone (Model 1) versus combined with RDW (Model 2).

Abbreviations: RDW, red cell distribution width; APACHE III, Acute Physiology and Chronic Health Evaluation III; SAPS II, Simplified Acute Physiology Score II; MELD, Model for End-Stage Liver Disease; SOFA, Sequential Organ Failure Assessment.

**S7 Fig. Subgroup analyses for **30-day mortality.****


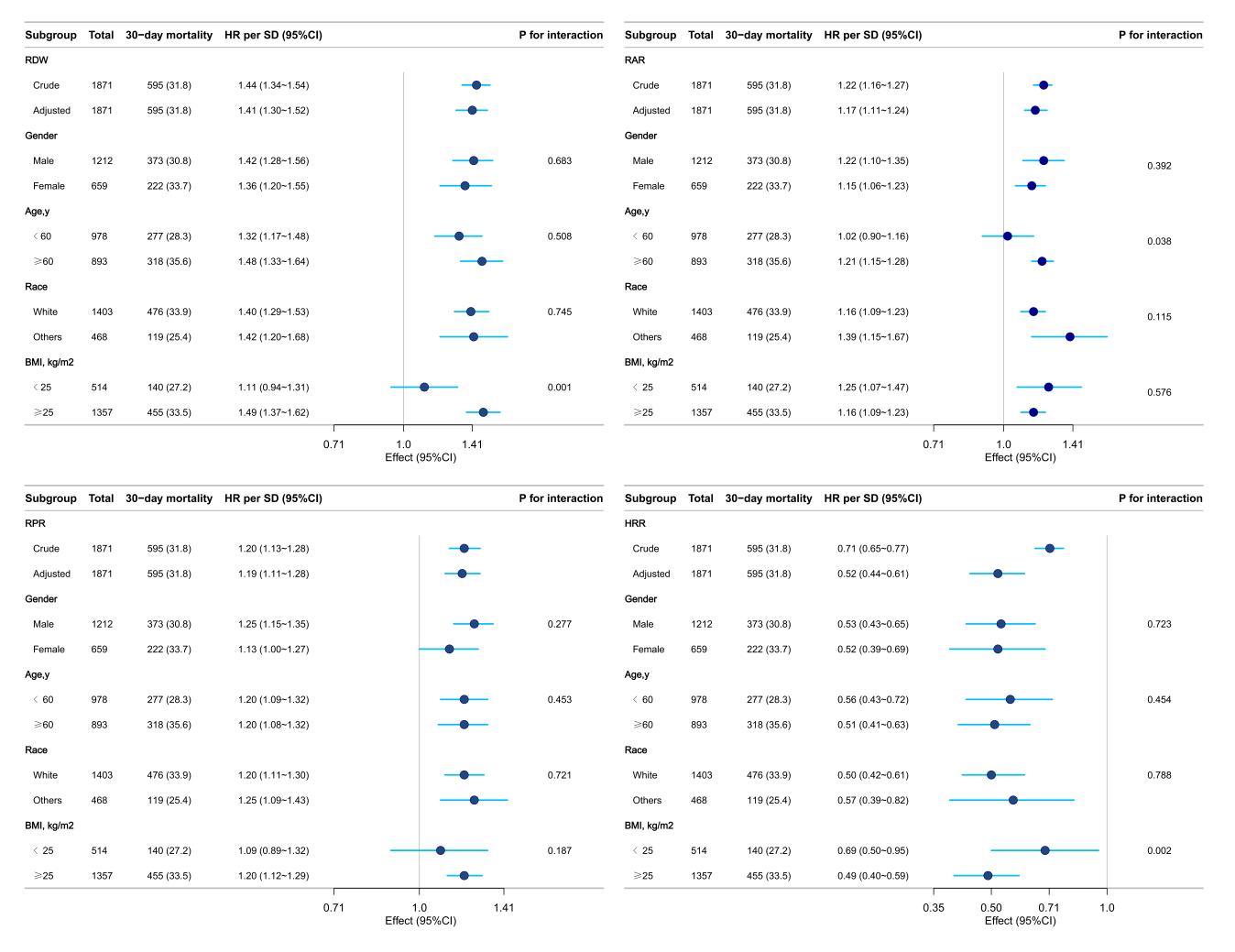


Models of RDW and RPR were adjusted for sex, age, race, BMI, Charlson Comorbidity Index, vasopressin use, albumin use, mechanical ventilation, renal replacement therapy, hemoglobin, hematocrit, albumin, potassium, lactate; Model for RAR excluded albumin from adjustment; Model for HRR excluded hemoglobin from adjustment.

Abbreviations: RDW, red cell distribution width; RAR, red cell distribution width-to-albumin ratio; RPR, red cell distribution width-to-platelet ratio; HRR, hemoglobin-to-red cell distribution width; BMI, body mass index; HR, Hazard Ratio; CI, Confidence Interval;

**S8 Fig. Subgroup analyses for **90-day mortality.****


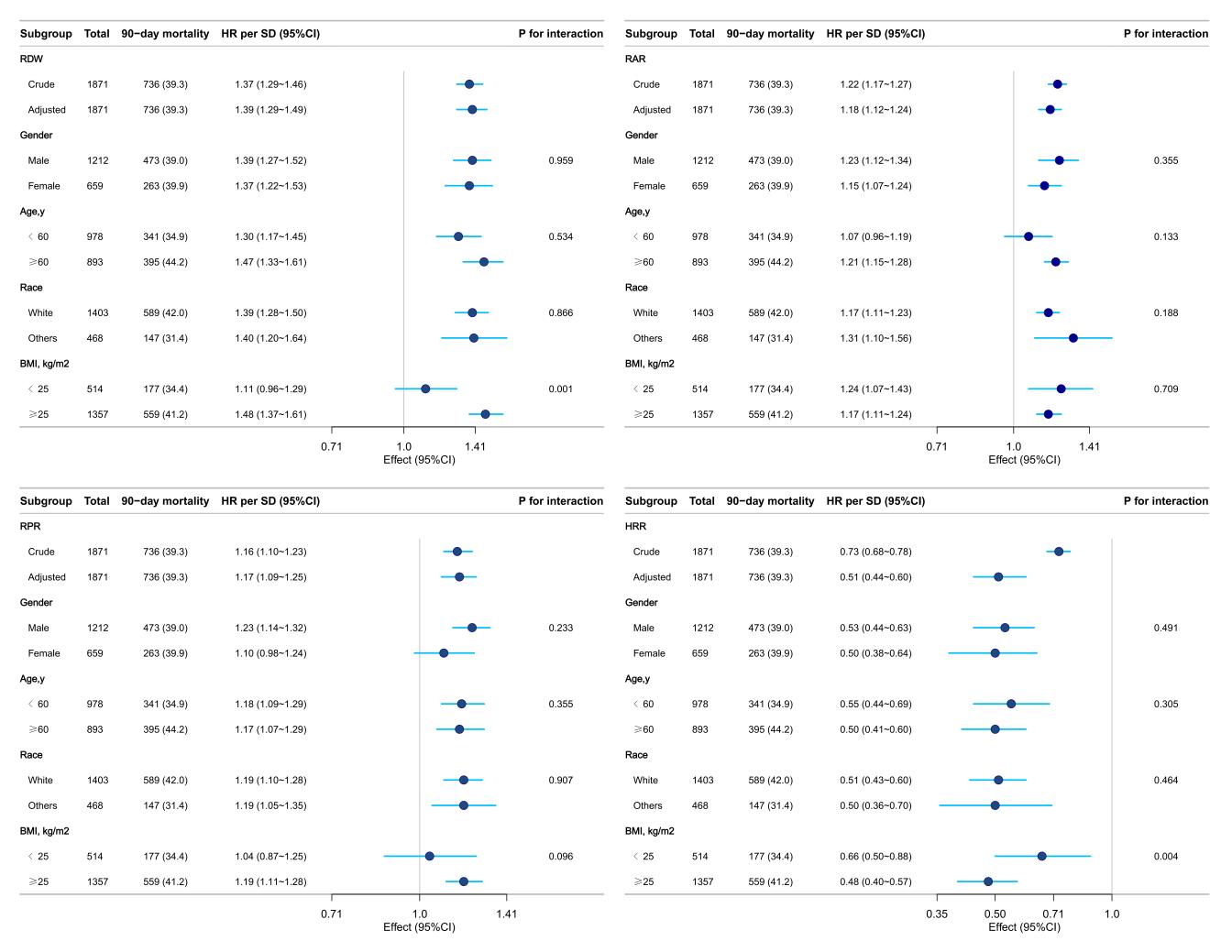


Models of RDW and RPR were adjusted for sex, age, race, BMI, Charlson Comorbidity Index, vasopressin use, albumin use, mechanical ventilation, renal replacement therapy, hemoglobin, hematocrit, albumin, potassium, lactate; Model for RAR excluded albumin from adjustment; Model for HRR excluded hemoglobin from adjustment.

Abbreviations: RDW, red cell distribution width; RAR, red cell distribution width-to-albumin ratio; RPR, red cell distribution width-to-platelet ratio; HRR, hemoglobin-to-red cell distribution width; BMI, body mass index; HR, Hazard Ratio; CI, Confidence Interval;

**S9 Fig. Subgroup analyses for **365-day mortality.****


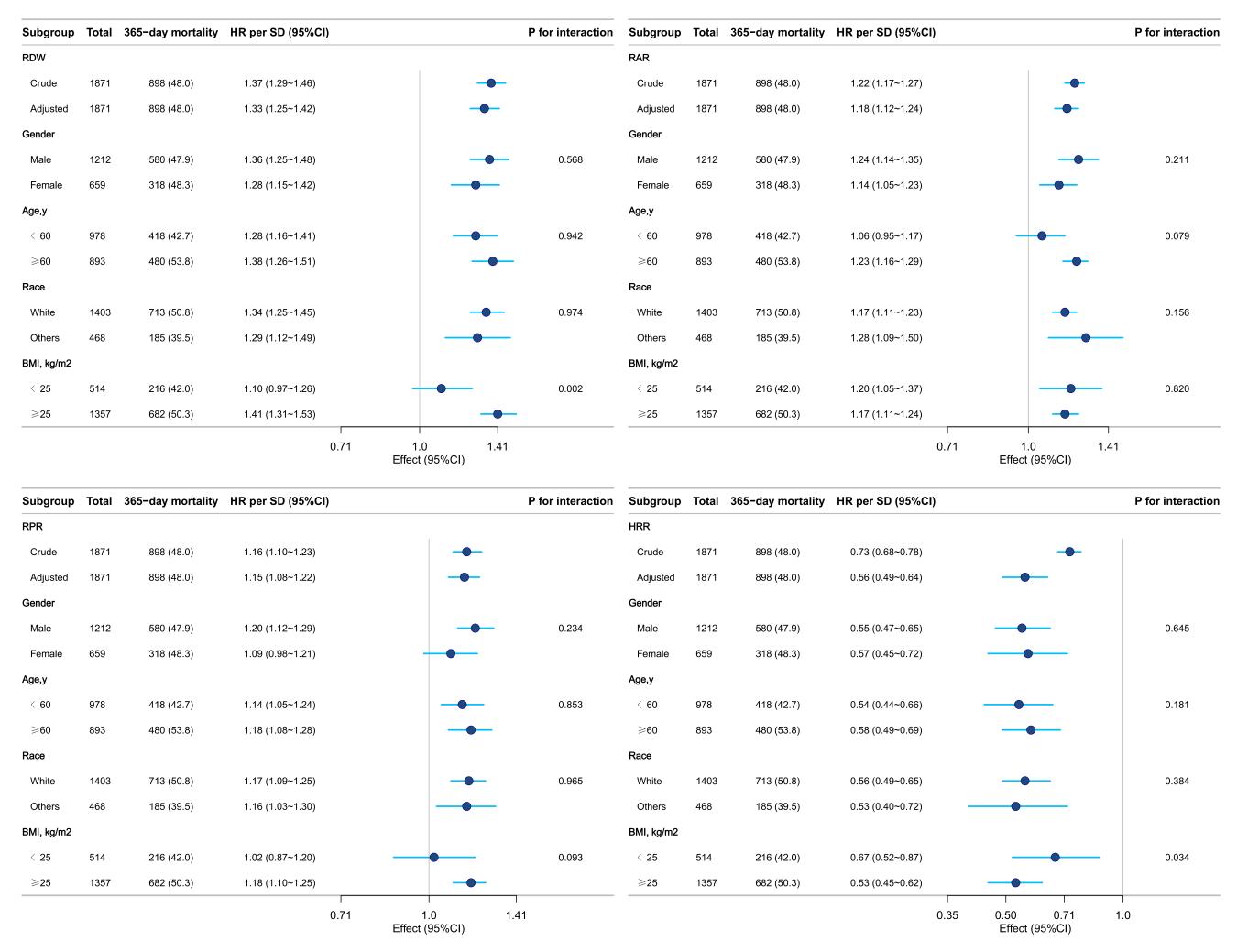


Models of RDW and RPR were adjusted for sex, age, race, BMI, Charlson Comorbidity Index, vasopressin use, albumin use, mechanical ventilation, renal replacement therapy, hemoglobin, hematocrit, albumin, potassium, lactate; Model for RAR excluded albumin from adjustment; Model for HRR excluded hemoglobin from adjustment.

Abbreviations: RDW, red cell distribution width; RAR, red cell distribution width-to-albumin ratio; RPR, red cell distribution width-to-platelet ratio; HRR, hemoglobin-to-red cell distribution width; BMI, body mass index; HR, Hazard Ratio; CI, Confidence Interval;
