# Supplementary material for: Association between Red Cell Distribution Width (RDW)-related inflammatory biomarkers and prognosis in ICU Cirrhosis patients: Evidence from the MIMIC-IV
Source: PLoS One. 2026 Jun 26;21(6):e0352023. doi: 10.1371/journal.pone.0352023 (PMC13308776; doi:10.1371/journal.pone.0352023)
Supplement: Attachment — Submitted filename: PONE-D-25-67953_reviewer RDW Reviewer comments.pdf [file pone.0352023.s003.pdf]

# PLOS One

# Association between Red Cell Distribution Width (RDW)-Related Inflammatory Biomarkers and Prognosis in ICU Cirrhosis Patients: Evidence from MIMIC-IV

--Manuscript Draft--

| | |
|---|---|
| **Manuscript Number:** | PONE-D-25-67953 |
| **Article Type:** | Research Article |
| **Full Title:** | Association between Red Cell Distribution Width (RDW)-Related Inflammatory Biomarkers and Prognosis in ICU Cirrhosis Patients: Evidence from MIMIC-IV |
| **Short Title:** | RDW-Related Inflammatory Biomarkers and Prognosis in Cirrhosis Patients |
| **Corresponding Author:** | Ying yi Li<br>The First Hospital of Quanzhou Affiliated to Fujian Medical University, Quanzhou, China<br>Quanzhou, CHINA |
| **Keywords:** | RDW; cirrhosis; intensive care unit; mortality; inflammatory biomarkers; prognosis |
| **Abstract:** | Background<br><br>Cirrhosis signifies a critical liver disease phase with high mortality, particularly in intensive care units (ICUs). Red cell distribution width (RDW) demonstrates prognostic value in various conditions, with evidence linking it to inflammation and adverse outcomes in liver disease. However, the predictive utility of RDW and its derived indices in ICU cirrhosis patients remains undetermined. This study investigated associations between RDW-related biomarkers and prognosis in critically ill cirrhosis patients.<br><br>Methods<br><br>A retrospective analysis ~~used~~ data from the Medical Information Mart for Intensive Care IV (MIMIC-IV) database, including 1,871 adults with cirrhosis at first ICU admission. Analysed biomarkers: RDW, RDW-to-albumin ratio (RAR), RDW-to-platelet ratio (RPR), and haemoglobin-to-RDW ratio (HRR). Primary outcomes: in-hospital, 90-day, and 365-day mortality. Adjusted multivariate Cox regression models determined biomarker-mortality associations. Receiver operating characteristic (ROC) curve analyses evaluated predictive performance.<br><br>Results<br><br>The cohort (mean age 58.8 ± 12.3 years; 64.8% male) had mortality rates of 26.7% (in-hospital), 39.3% (90-day), and 48.0% (365-day). Mortality was consistently higher in high versus low RDW groups. After adjustment, all biomarkers independently predicted mortality. Each unit increase in RDW conferred a 14% higher mortality risk (in-hospital HR 1.14, 95% CI 1.10–1.19; P < 0.001) RAR increased risk by 10% across endpoints. RPR showed substantially elevated risks: 160% (HR 2.60), 130% (HR 2.30), and 123% (HR 2.23) for in-hospital, 90-day, and 365-day mortality, respectively. HRR exhibited protective effects, reducing risk by 99% (all endpoints HR 0.01, 95% CI 0.00–0.04; P < 0.001) ROC analysis revealed RDW had the highest predictive value (AUC 63.7%), followed by HRR (AUC 62.1%), RAR (AUC 59.7%), and RPR (AUC 55.1%). Combined with SAPS II, MELD, or SOFA, RDW provided incremental predictive value (absolute AUC increase 0.6–1.6%).<br><br>Conclusion<br><br>RDW-related biomarkers, particularly RDW and HRR, significantly associate with mortality in ICU cirrhosis patients, serving as potentially useful adjunctive prognostic markers. These accessible, cost-effective biomarkers enhance risk stratification and clinical decision-making. Integrating RDW-based assessments into routine prognostic evaluation may optimise patient management and resource allocation in ICU settings |
| **Order of Authors:** | Xiaodong Zhu |

| | |
|---|---|
| | Chanchan Lin |
| | Xiaoqiang Liu |
| | Zicheng Huang |
| | Ying yi Li |
| **Additional Information:** | |
| **Question** | **Response** |
| **Financial Disclosure**<br><br>Enter a financial disclosure statement that describes the sources of funding for the work included in this submission. Review the submission guidelines for detailed requirements. View published research articles from *PLOS ONE* for specific examples.<br><br>This statement is required for submission and **will appear in the published article** if the submission is accepted. Please make sure it is accurate.<br><br>**Funded studies**<br>Enter a statement with the following details:<br>• Initials of the authors who received each award<br>• Grant numbers awarded to each author<br>• The full name of each funder<br>• URL of each funder website<br>• Did the sponsors or funders play any role in the study design, data collection and analysis, decision to publish, or preparation of the manuscript?<br><br>Did you receive funding for this work? | Yes |
| Please add funding details.<br>as follow-up to "**Financial Disclosure**<br><br>Enter a financial disclosure statement that describes the sources of funding for the work included in this submission. Review the submission guidelines for detailed requirements. View published research articles from *PLOS ONE* for specific examples.<br><br>This statement is required for submission | This research was funded by the Startup Fund for scientific studies at Fujian Medical University (Grant No. 2023QH1310) and the Quanzhou City Science & Technology Program in China (Grant No. 2024NY001). The funding sources had no role in the design of the study, collection and analysis of data, interpretation of results, and decision to publish. |

| | |
|---|---|
| and **will appear in the published article** if the submission is accepted. Please make sure it is accurate.<br><br>**Funded studies**<br>Enter a statement with the following details:<br>• Initials of the authors who received each award<br>• Grant numbers awarded to each author<br>• The full name of each funder<br>• URL of each funder website<br>• Did the sponsors or funders play any role in the study design, data collection and analysis, decision to publish, or preparation of the manuscript?<br><br>Did you receive funding for this work?" | |
| Please select the country of your main research funder (please select carefully as in some cases this is used in fee calculation).<br>as follow-up to "**Financial Disclosure**<br><br>Enter a financial disclosure statement that describes the sources of funding for the work included in this submission. Review the submission guidelines for detailed requirements. View published research articles from *PLOS ONE* for specific examples.<br><br>This statement is required for submission and **will appear in the published article** if the submission is accepted. Please make sure it is accurate.<br><br>**Funded studies**<br>Enter a statement with the following details:<br>• Initials of the authors who received each award<br>• Grant numbers awarded to each author<br>• The full name of each funder<br>• URL of each funder website<br>• Did the sponsors or funders play any role in the study design, data collection and analysis, decision to publish, or preparation of the manuscript? | CHINA - CN |

| | |
|---|---|
| Did you receive funding for this work?" | |
| **Competing Interests**<br><br>Use the instructions below to enter a competing interest statement for this submission. On behalf of all authors, disclose any competing interests that could be perceived to bias this work—acknowledging all financial support and any other relevant financial or non-financial competing interests.<br><br>This statement is required for submission and **will appear in the published article** if the submission is accepted. Please make sure it is accurate and that any funding sources listed in your Funding Information later in the submission form are also declared in your Financial Disclosure statement.<br><br>View published research articles from *PLOS ONE* for specific examples.<br><br>**NO authors have competing interests**<br><br>Enter: *The authors have declared that no competing interests exist.*<br><br>**Authors with competing interests**<br><br>Enter competing interest details beginning with this statement:<br><br>*I have read the journal's policy and the authors of this manuscript have the following competing interests: [insert competing interests here]*<br><br>* typeset | The authors have declared that no competing interests exist. |
| **Ethics Statement**<br><br>Enter an ethics statement for this submission. This statement is required if the study involved: | N/A |

Powered by Editorial Manager® and ProduXion Manager® from Aries Systems Corporation

- Human participants
- Human specimens or tissue
- Vertebrate animals or cephalopods
- Vertebrate embryos or tissues
- Field research

Write "N/A" if the submission does not require an ethics statement.

General guidance is provided below. Consult the submission guidelines for detailed instructions. **Make sure that all information entered here is included in the Methods section of the manuscript.**

Powered by Editorial Manager® and ProduXion Manager® from Aries Systems Corporation

| | |
|---|---|
| **Format for specific study types**<br><br>**Human Subject Research (involving human participants and/or tissue)**<br>• Give the name of the institutional review board or ethics committee that approved the study<br>• Include the approval number and/or a statement indicating approval of this research<br>• Indicate the form of consent obtained (written/oral) or the reason that consent was not obtained (e.g. the data were analyzed anonymously)<br><br>**Animal Research (involving vertebrate animals, embryos or tissues)**<br>• Provide the name of the Institutional Animal Care and Use Committee (IACUC) or other relevant ethics board that reviewed the study protocol, and indicate whether they approved this research or granted a formal waiver of ethical approval<br>• Include an approval number if one was obtained<br>• If the study involved *non-human primates*, add *additional details* about animal welfare and steps taken to ameliorate suffering<br>• If anesthesia, euthanasia, or any kind of animal sacrifice is part of the study, include briefly which substances and/or methods were applied<br><br>**Field Research**<br><br>Include the following details if this study involves the collection of plant, animal, or other materials from a natural setting:<br>• Field permit number<br>• Name of the institution or relevant body that granted permission | |
| **Data Availability**<br><br>Authors are required to make all data underlying the findings described fully available, without restriction, and from the time of publication. PLOS allows rare exceptions to address legal and ethical concerns. See the PLOS Data Policy and FAQ for detailed information. | Yes - all data are fully available without restriction |

Powered by Editorial Manager® and ProduXion Manager® from Aries Systems Corporation

| | |
|---|---|
| A Data Availability Statement describing where the data can be found is required at submission. Your answers to this question constitute the Data Availability Statement and **will be published in the article**, if accepted.<br><br>**Important:** Stating 'data available on request from the author' is not sufficient. If your data are only available upon request, select 'No' for the first question and explain your exceptional situation in the text box.<br><br>Do the authors confirm that all data underlying the findings described in their manuscript are fully available without restriction? | |
| **Describe where the data may be found in full sentences. If you are copying our sample text, replace any instances of XXX with the appropriate details.**<br><br>• If the data are **held or will be held in a public repository**, include URLs, accession numbers or DOIs. If this information will only be available after acceptance, indicate this by ticking the box below. For example: *All XXX files are available from the XXX database (accession number(s) XXX, XXX.).*<br>• If the data are all contained **within the manuscript and/or Supporting Information files**, enter the following: *All relevant data are within the manuscript and its Supporting Information files.*<br>• If neither of these applies but you are able to provide **details of access elsewhere**, with or without limitations, please do so. For example:<br><br>*Data cannot be shared publicly because of [XXX]. Data are available from the XXX Institutional Data Access / Ethics Committee (contact via XXX) for researchers who meet the criteria for access to confidential data.*<br><br>*The data underlying the results presented in the study are available from (include the name of the third party* | The data is available in the MIMIC repository, https://mimic.physionet.org/ |

| | |
|---|---|
| *and contact information or URL).*<br>• This text is appropriate if the data are owned by a third party and authors do not have permission to share the data.<br><br>* typeset | |
| Additional data availability information: | |

Manuscript Click here to access/download;Manuscript;Manuscript.docx

# Association between Red Cell Distribution Width (RDW)-Related Inflammatory Biomarkers and Prognosis in ICU Cirrhosis Patients: Evidence from MIMIC-IV

Xiaodong Zhu[a], Chanchan Lin[a], Xiaoqiang Liu[a], Zicheng Huang[a**],Yingyi Li[a**]

a Department of Gastroenterology, The First Hospital of Quanzhou Affiliated to Fujian Medical University, Quanzhou, China

*Correspondence to Yingyi Li, Master, Department of Gastroenterology, The First Hospital of Quanzhou Affiliated to Fujian Medical University, Quanzhou, Fujian, 362000, China. E-mail: liyingyi0924@126.com; Zicheng Huang, Bachelor, Department of Gastroenterology, The First Hospital of Quanzhou Affiliated to Fujian Medical University, Quanzhou, Fujian, 362000, China. E-mail: hzcpwm@126.com

## Abstract

**Background** Cirrhosis signifies a critical liver disease phase with high mortality, particularly in intensive care units (ICUs). Red cell distribution width (RDW) demonstrates prognostic value in various conditions, with evidence linking it to inflammation and adverse outcomes in liver disease. However, the predictive utility of RDW and its derived indices in ICU cirrhosis patients remains undetermined. This study investigated

associations between RDW-related biomarkers and prognosis in critically ill cirrhosis patients.

**Methods** A retrospective analysis used data from the Medical Information Mart for Intensive Care IV (MIMIC-IV) database, including 1,871 adults with cirrhosis at first ICU admission. Analysed biomarkers: RDW, RDW-to-albumin ratio (RAR), RDW-to-platelet ratio (RPR), and haemoglobin-to-RDW ratio (HRR). Primary outcomes: in-hospital, 90-day, and 365-day mortality. Adjusted multivariate Cox regression models determined biomarker-mortality associations. Receiver operating characteristic (ROC) curve analyses evaluated predictive performance.

**Results** The cohort (mean age 58.8 ± 12.3 years; 64.8% male) had mortality rates of 26.7% (in-hospital), 39.3% (90-day), and 48.0% (365-day). Mortality was consistently higher in high versus low RDW groups. After adjustment, all biomarkers independently predicted mortality. Each unit increase in RDW conferred a 14% higher mortality risk (in-hospital HR 1.14, 95% CI 1.10–1.19; P < 0.001) RAR increased risk by 10% across endpoints. RPR showed substantially elevated risks: 160% (HR 2.60), 130% (HR 2.30), and 123% (HR 2.23) for in-hospital, 90-day, and 365-day mortality, respectively. HRR exhibited protective effects, reducing risk by 99% (all endpoints HR 0.01, 95% CI 0.00–0.04; P < 0.001) ROC analysis revealed RDW had the highest predictive value (AUC 63.7%), followed by HRR (AUC 62.1%), RAR (AUC 59.7%), and

RPR (AUC 55.1%). Combined with SAPS II, MELD, or SOFA, RDW provided incremental predictive value (absolute AUC increase 0.6–1.6%).
**Conclusion** RDW-related biomarkers, particularly RDW and HRR, significantly associate with mortality in ICU cirrhosis patients, serving as potentially useful adjunctive prognostic markers. These accessible, cost-effective biomarkers enhance risk stratification and clinical decision-making. Integrating RDW-based assessments into routine prognostic evaluation may optimise patient management and resource allocation in ICU settings

**Keywords:** RDW, cirrhosis, intensive care unit, mortality, inflammatory biomarkers, prognosis

## Introduction

Cirrhosis is a progressive liver disease characterized by the replacement of the healthy liver parenchyma with fibrotic tissue and regenerative nodules, which ultimately leads to portal hypertension and liver dysfunction. This condition can arise from various causes, including chronic alcohol consumption, hepatitis B or C infection, nonalcoholic fatty liver disease, and autoimmune diseases. The disease progresses from an asymptomatic phase, known as compensated cirrhosis, to a symptomatic phase, known as decompensated cirrhosis, where complications such as ascites, variceal bleeding, and hepatic encephalopathy become prevalent. The management of cirrhosis focuses on treating the underlying causes and complications, with liver transplantation being a potential ~~requirement~~ in severe cases [1].

Decompensated cirrhosis represents a critical stage in liver disease where the liver can no longer perform its essential functions, leading to severe complications and a high mortality rate. Despite advancements in medical therapies, the prognosis of patients with decompensated cirrhosis remains poor, necessitating a deeper understanding of the disease and the development of more effective treatment strategies [2]. Furthermore, the management of critically ill patients with cirrhosis in the intensive care setting presents additional challenges. The high mortality rate of these patients is often due to multiple organ failure, necessitating a

multidisciplinary approach that includes liver-centric intensive care protocols. These protocols focus on mitigating the high mortality associated with advanced and decompensated cirrhosis by implementing organ support therapies, infection control, and palliative care [3]. Thus, the importance of identifying reliable prognostic markers in this patient population cannot be overstated. Patients with cirrhotic in the intensive care unit (ICU) represent a particularly vulnerable group with high mortality rates [4]. Early risk stratification and an accurate prognostic assessment are crucial for optimal patient management and resource allocation [5].

Red cell distribution width (RDW) is a routinely measured parameter that reflects heterogeneity in the size of circulating red blood cells (RBCs)[6]. In recent years, RDW has emerged as a valuable prognostic biomarker in various clinical conditions, particularly in critical care settings [7]. The clinical significance of RDW extends beyond its traditional role in differentiating types of anaemia, as mounting evidence suggests its strong association with inflammatory processes and adverse outcomes [8]. Inflammation plays a crucial role in the pathogenesis and progression of liver cirrhosis. Recent studies have demonstrated that elevated RDW levels are significantly associated with poor outcomes in patients with liver diseases [9]. The relationship between RDW and inflammation has been well documented, with several studies showing

strong correlations between RDW and traditional inflammatory markers such as C-reactive protein (CRP) and erythrocyte sedimentation rate (ESR) [10].

However, the predictive values of RDW and its derived indices in patients with cirrhosis admitted to ICU remain unclear. Therefore, this study aimed to investigate the association between RDW-related inflammatory biomarkers and prognosis in ICU patients with cirrhosis using the Medical Information Mart for Intensive Care IV (MIMIC-IV) database. The findings from this study could potentially identify novel prognostic tools for this high-risk patient population and inform clinical decision making in critical care settings.

## Materials and Methods

### Data

This study was a retrospective cohort analysis that utilized data from a large critical database, the Medical Information Mart for Intensive Care IV (MIMIC-IV). MIMIC-IV 3.0 is a comprehensive, single-center longitudinal database that encompasses data on 364 627 patients admitted to the Beth Israel Deaconess Medical Center, including 94 458 intensive care unit (ICU) stays, spanning the period from 2008 to 2022 (https://mimic.mit.edu) [11]. This study was approved by the institutional

review boards of both the Massachusetts Institute of Technology and BIDMC. Yingyi Li is accountable for extracting data and has been given access to the MIMIC database (Record ID: 62389476).

## Study Population Selection Criteria

The inclusion criteria were as follows: (1) patients diagnosed with cirrhosis based on the International Classification of Diseases, 9th Edition (ICD-9) and 10th Edition (ICD-10) codes and (2) first-time ICU admission. All patients were initially enrolled in the study and were aged 18 years or older. Patients were excluded based on the following exclusion criteria: (1) absence of RDW, albumin, platelet, and hemoglobin information on the initial ICU day, (2) lack of data on in-hospital mortality. Ultimately, 1 871 ICU cirrhosis patients were obtained from MIMIC-IV database (Fig. 1).

## RDW-Related Biomarkers

The following parameters were analyzed: RDW, RDW-to-albumin ratio (RAR), RDW-to-platelet ratio (RPR), and hemoglobin-to-RDW ratio (HRR). RDW, albumin, platelets, and hemoglobin were extracted within 24h after ICU admission. The median of each inflammatory biomarker was used as a point to divide them into two groups: the low and high groups (RDW 16.9%, RAR 5.86, RPR 0.17, HRR 0.55).

## Outcomes

The primary outcomes included in-hospital, 90-day, and 365-day mortality. In-hospital mortality was strictly defined as death from any cause during the entire hospitalization period (including post-ICU ward stays), not limited to the index ICU admission. This captures the full clinical trajectory of decompensated cirrhosis patients who often experience prolonged hospitalizations beyond ICU discharge. Additionally, we assessed five cirrhosis-related complications: variceal hemorrhage, ascites, spontaneous bacterial peritonitis, hepatic encephalopathy, and hepatorenal syndrome.

## Data extraction

Structured Query Language (SQL) and the PostgreSQL tool were used to extract data from the MIMIC-IV database. The extracted information included demographic data, vital signs, complications, laboratory indicators, clinical evaluations, and treatment management. Demographic information included age, sex, race, and body mass index (BMI). The vital parameters included respiratory rate, pulse oximetry, temperature, blood pressure, and heart rate. Complications included hypertension, diabetes, chronic pulmonary disease, myocardial infarction, congestive heart failure, renal disease, cerebrovascular disease, malignant cancer,

malnutrition, and peptic ulcer disease. Clinical evaluations included the Charlson Comorbidity Index, Acute Physiology Score III, and etiology of cirrhosis. Therapeutic management included albumin, mechanical ventilation, vasopressin, and renal replacement therapy (RRT). In addition to RDW-related parameters, other laboratory indicators included white blood cells, total bilirubin, aspartate aminotransferase (AST), alanine aminotransferase (ALT), creatinine, urea nitrogen, glucose, sodium, potassium, chloride, calcium, anion gap, and lactate. The number and percentage of missing values for each covariate are presented in Supplementary Table 3.

## Statistical analyses

Continuous and normally distributed variables are represented as mean ± standard deviation (SD). For variables that were continuous and non-normally distributed, the median with interquartile range (IQR) is presented. Categorical variables were reported as frequencies and their respective percentages. The cumulative rates of death were compared using Kaplan - Meier curves. To resolve missing data issues, multiple imputations were performed using chained equations with 20 imputations, to secure unbiased estimates of the association between RDW and the outcome. The impact of RDW-related indices on different outcomes was assessed through binary Cox regression models that were adjusted for key

covariates [12-15]. We selected covariates based on clinical judgment and previous literature. To assess potential confounders, we introduced each covariate individually into the baseline Cox regression model and compared changes in the regression coefficients. Covariates that caused a change of more than 10% in the initial regression coefficient were retained. Subsequently, we excluded variables with a variance inflation factor (VIF) $\geq 5$ and any potential mediators. The final set of retained covariates was included in the fully adjusted model. We employed Cox proportional hazards regression models incorporating cubic spline functions and smooth curve fitting to identify potential non-linear relationships between various RDW-derived indices and the primary outcome (in-hospital mortality). If a non-linear association was detected, inflection points were first determined using a recursive algorithm, followed by the construction of piecewise Cox proportional hazards regression models on either side of the identified inflection points. The subgroup analyses were performed using stratified Cox proportional hazards models. Interaction among subgroups was inspected by the likelihood ratio test. Sensitivity analysis was performed to assess the robustness of all outcomes to unmeasured confounding variables using the E-value methodology of VanderWeele and Ding [16]. Receiver Operating Characteristic (ROC) analysis was used to evaluate the ability of RDW, RAR, RPR, and HRR to predict mortality during the hospital

stay, as well as at 90 and 365 days after ICU admission. The ROC-derived thresholds (Table 3) was determined by maximizing the Youden index (sensitivity + specificity – 1) on the ROC curve, thereby achieving the optimal balance between sensitivity and specificity for clinical application. Moreover, we further compared the predictive capabilities of the APACHE III (APS III), SAPS II, MELD, and SOFA scores, as well as those of their respective combined models with RDW. The sensitivity and specificity were determined for each indicator, and the area under the curve (AUC) was calculated. Statistical analyses were performed using R software (http://www.r-project.org, The R Foundation) and Free Statistics software version 2.1.0.

# Results

## Baseline Characteristics

The study included 1 871 cirrhosis patients, with a mean age of 58.8 ± 12.3 years and 64.8% male patients. Baseline demographic and clinical characteristics of the RDW group are presented in Table 1. The high RDW group had lower blood pressure, lower temperature, higher APS III scores, more alcohol cirrhosis, more albumin use and more RRT. The proportion of patients with hypertension, diabetes, chronic pulmonary disease, and malignant cancer was lower in the high RDW group; however, the proportion of peptic ulcer disease and malnutrition was

higher in the high RDW group. The levels of white blood cells, total bilirubin, creatinine, urea nitrogen, anion gap, and lactate were significantly higher in the high RDW group than in the low RDW group. However, the levels of hemoglobin, platelet count, ALT, glucose, and chloride in the high RDW group were significantly lower than those in the low RDW group (Table 1).

## Short- And Long-term Mortality

In 1 871 patients, in-hospital mortality, 90-day mortality, and 365-day mortality rates were 26.7%, 39.3%, and 48.0%, respectively. Among these, 11.8% had variceal bleeding, 52.0% had ascites, 17.2% had hepatorenal syndrome, 10.2% had hepatic encephalopathy, and 11.8% had spontaneous bacterial peritonitis (Table 1). Supplementary Fig. 1-3 show the results of the Kaplan-Meier method concerning in-hospital mortality, 90-day and 365-day mortality analyses according to RDW, RAR, RPR, and HRR levels. In the high RDW, high RAR, high RPR, and low HRR groups, the in-hospital, 90-day, and 365-day mortality rates were all significantly higher than those in the reference group.

## Association Between RDW-related Biomarkers And Mortality

To explore the association between RDW-related inflammatory biomarkers and mortality, we conducted Cox regression analysis. The

results of univariate COX analysis of mortality rates at different times are presented in Supplementary Table 4. Table 2 shows the associations between all mortality outcomes and RDW-related indices. Based on the findings of our investigation, RDW, RAR, and RPR were positively associated with all mortality outcomes, whereas HRR was negatively associated with all mortality outcomes in the crude model of univariate analysis (Table 2, crude model). After full adjustment (Table 2, adjusted model), RDW, RAR, RPR, and HRR remained significantly associated with all the mortality outcomes. After multivariable adjustment, each one-unit increase in RDW was associated with a 14 % higher risk of in-hospital (HR 1.14; 95 % CI 1.10–1.19), 90-day (HR 1.14; 95 % CI 1.10–1.18), and 365-day mortality (HR 1.14; 95 % CI 1.10–1.18; all $P < 0.001$). For RAR, the corresponding hazard rose by 10 % across all three time-points (HR 1.10; 95 % CI 1.03–1.17 for in-hospital, 1.05–1.17 for 90-day, and 1.05–1.16 for 365-day mortality; all $P < 0.001$). In contrast, each one-unit increase in RPR conferred a markedly elevated risk: 160 % for in-hospital (HR 2.60; 95 % CI 1.37–4.94), 130 % for 90-day (HR 2.30; 95 % CI 1.29–4.08), and 123 % for 365-day mortality (HR 2.23; 95 % CI 1.29–3.83; all $P < 0.05$). Finally, each one-unit increase in HRR was associated with a 99 % risk reduction across all three endpoints (HR 0.01; 95 % CI 0.00–0.04 for in-hospital, 0.00–0.03 for 90-day, and 0.00–0.03 for 365-day mortality; all $P < 0.001$).

**Assessment of Nonlinear Relationships Between RDW-related Biomarkers And In-hospital Mortality**

~~Fig. 2 presents the~~ multivariable-adjusted restricted cubic spline curves illustrating the relationships between various RDW-derived indices and in-hospital mortality. Accordingly, in the restricted cubic spline analysis, RDW and RPR exhibited linear associations with in-hospital mortality, whereas RAR showed an inverted L-shaped non-linear relationship, and HRR demonstrated an L-shaped non-linear relationship. Inflection point analysis indicated saturation effects at an HRR of 0.641. When the RAR reached 6.62, the hazard ratio (HR) began to decline, and the curve to the right of the inflection point became relatively flat. (Supplementary Table 1).

## Predictive Performances of RDW-related Biomarkers

ROC analysis was used to evaluate the predictive capability of the RDW-related indices. Fig. 3 shows the comparison of AUC values between RDW, RAR, RPR, and HRR for in-hospital, 90-day, and 365-day mortality rates. Table 3 presents the AUC, sensitivity, and specificity of all ROC curves. ROC analysis revealed that RDW had the highest AUC for in-hospital mortality (63.22%), 90-day mortality rate (63.66%), and 365-day mortality (62.48%), while RPR had the lowest

AUC for in-hospital mortality (55.95%), 90-day mortality rate (54.29%), and 365-day mortality (54.55%). The AUC of HRR was close to that of RDW. RDW showed good sensitivity for in-hospital mortality (0.661). The HRR showed good specificity for in-hospital mortality (0.730) and good sensitivity for 90-day mortality (0.644). The AUC for RDW and its three derived inflammatory markers was found to be lower than that of existing prognostic scoring systems (APS III, SAPS II, MELD, and SOFA scores). However, we observed that RDW provided incremental value when combined with SAPS II, MELD, and SOFA scores, with AUC improvements ranging from 0.6% to 1.6% (all $p$ values < 0.05) (Table 4).

## Sensitivity Analysis

The results of the subgroup analyses are presented in Supplementary Fig. 4. We found that the associations between different RDW-derived indices and in-hospital mortality remained significant across all subgroups, indicating robustness of the conclusions. However, with the exception of RAR, we also observed a significant interaction between in-hospital mortality and different BMI strata (P for interaction < 0.05). Uncontrolled confounding factors were assessed by calculating the E value. The E-values of the RDW, RAR, RPR, and HRR for all mortality outcomes

are presented in Supplementary Table 2. These relatively large E-values indicate that all models were robust.

## Discussion

Our large-scale cohort study focused on the predictive value of RDW and the three additional RDW-derived inflammatory markers (RAR, RPR, and HRR) in ICU patients with cirrhosis, including both short- and long-term mortality. Our findings reveal a significant association between these biomarkers and mortality in ICU patients with cirrhosis. In the evaluation of in-hospital, 90-day, and 365-day mortality, RDW exhibited moderate predictive efficacy, outperforming the other three indicators. For critically ill patients with cirrhosis, it is not recommended to use RDW-related inflammatory markers as standalone diagnostic tools. However, RDW may potentially serve as a supplementary instrument to enhance the capacity for risk stratification.

The association between RDW and mortality in ICU patients is a subject of considerable interest. RDW is a measure of the variation in the size of RBCs and has been identified as a predictor of mortality in various clinical settings, including ICU patients [7, 17]. Numerous studies have aimed to clarify the relationship between RDW and different liver diseases, including chronic viral hepatitis, nonalcoholic fatty liver disease, primary biliary cholangitis, and hepatocellular carcinoma [18-22]. Previous

studies have demonstrated a close association between RDW and liver injury and hepatic fibrosis [18, 23-25]. Chronic liver injury leads to an increase in pro-inflammatory factors, which in turn affect the production of erythropoietin and the utilization of iron. This ultimately results in alterations to the morphology and apoptosis of RBCs. Additionally, nutritional deficiencies and cachexia may also contribute to anisocytosis and increase RDW [18]. Consequently, RDW may serve as a potential prognostic marker for chronic liver disease. Consistent with the majority of prior research findings, our study also confirmed a significant correlation between RDW and prognosis in patients with severe cirrhosis, encompassing both short- and long-term mortality [20, 26, 27]. Moreover, in our study population, RDW demonstrated superior prognostic discriminative ability compared with other RDW-related indices.

RAR has also been explored as a prognostic indicator. The RAR represents the combined effect of inflammation (denoted by elevated RDW) and impaired hepatic synthetic function (reflected by reduced albumin). In patients with sepsis, higher RAR has been associated with poor clinical outcomes, including increased mortality. This suggests that RAR could serve as a useful marker for assessing the prognosis of critically ill patients [28]. However, there are currently few studies on RAR and liver disease. Mao et al. found that in a cohort of 167 patients diagnosed with hepatitis B virus (HBV)-related compensated cirrhosis,

the RAR levels were higher in non-survivors than in survivors. Elevated RAR levels were significantly associated with adverse outcomes, and there was no significant difference in predictive ability between RAR levels and the Model for End-Stage Liver Disease (MELD) score (RAR: AUC = 0.773; MELD: AUC = 0.830) [29]. Tan et al. conducted a retrospective study of 1,413 patients with HBV-related hepatocellular carcinoma (HBV-HCC). They identified that a high RAR was an independent risk factor for long-term overall survival in patients with HBV-HCC. Compared with RDW alone, albumin, total bilirubin, and Child-Pugh score, RAR demonstrated superior prognostic discriminative ability, with an AUC of 0.751. Incorporating RAR into the traditional HCC staging system significantly enhances the ability to predict overall mortality risk [30]. Our study also found that RAR is an independent risk factor for mortality in critically ill patients with cirrhosis. However, its predictive performance did not exceed that of the RDW alone in our study population.

Similarly, higher RPR has been linked to increased mortality, indicating its potential as a prognostic tool in critical care settings [12]. Through the RPR, thrombocytopenia in portal hypertension potentiates the prognostic utility of RDW elevation. In the field of liver diseases, multiple studies have confirmed RPR as a robust predictor of liver fibrosis and cirrhosis, and it is recommended for use in noninvasive

clinical diagnosis [25, 31, 32]. Dallio et al. found that RPR demonstrated superiority over other non-invasive tools in predicting decompensation events in patients with metabolic-associated steatohepatitis liver disease (MASLD) and can serve as an effective predictive tool [33]. Zhang et al. analyzed the association between RPR and 30-day mortality in 168 patients with HBV-related decompensated cirrhosis. They found that RPR was an independent prognostic predictor, and combining RPR with the MELD score further enhanced its predictive value for mortality [9]. In our study, RPR was significantly associated with both short- and long-term mortality in critically ill patients with cirrhosis. However, its prognostic predictive value was not superior to that of RDW.

The HRR is another parameter that has been studied in relation to mortality. Although less commonly discussed, HRR may provide additional insights into patient prognosis, particularly in the context of critical illnesses [34-37]. The HRR combines hemoglobin levels and RDW elevation (reflecting inflammatory burden), serving as a critical biomarker in sepsis-associated cirrhosis decompensation. Currently, the only study reporting on the correlation between HRR and the prognosis of liver diseases was by Yu et al. Their research found that a low HRR was significantly associated with the 30-day mortality rate in patients with HBV-related decompensated cirrhosis. Thus, HRR may serve as a promising prognostic predictor for patients with liver diseases [14]. In our

cohort of 1871 ICU cirrhosis patients, a low HRR was significantly associated with in-hospital, 90-day, and 365-day mortality. It also demonstrated predictive capabilities comparable to those of RDW and superior to those of RAR and RPR. The prognostic value of HRR in patients with different liver diseases warrants further investigation through additional studies.

RDW-related inflammatory biomarkers are easily obtainable in clinical practice and are straightforward to calculate, potentially offering a predictive value for critically ill patients [38-43]. A significant practical advantage of RDW-related biomarkers is their inherent cost-effectiveness. Since RDW, albumin, platelets, and hemoglobin are routinely measured components of standard complete blood count (CBC) and comprehensive metabolic panel (CMP) tests in hospitalized patients, calculating ratios such as RAR, RPR, or HRR incurs no additional laboratory costs. This contrasts sharply with specialized inflammatory markers (e.g., CRP, procalcitonin) or proprietary prognostic scores requiring dedicated assays. The minimal incremental expense supports the feasibility of widespread implementation in resource-constrained settings. These biomarkers could help prioritize high-risk patients for intensive monitoring or early intervention, particularly where advanced scoring systems are unavailable. Different RDW-related inflammatory biomarkers may reflect distinct pathophysiological changes in patients admitted to the ICU.

## Strengths and limitations

Our large-sample cohort study is the first to confirm the independent associations of four commonly used RDW-related inflammatory biomarkers with both short-term and long-term prognoses in patients with cirrhosis in the ICU. We also conducted sensitivity analyses using unmeasured confounding E-values to confirm the robustness of Cox models. Additionally, we conducted a comparative analysis of the predictive capabilities of these four markers for short- and long-term prognoses. While our study identified median values as exploratory cut-points (e.g., RDW > 16.9%), definitive clinical thresholds for intervention require prospective validation. The sensitivity-specificity trade-offs in Table 3 inform context-dependent use. For example: RDW's moderate sensitivity (66.1%) supports its role in ruling out low-risk patients (i.e., RDW ≤16.75% reduces mortality likelihood). HRR's higher specificity (73.0%) makes it valuable for ruling in high-risk cases (i.e., HRR <0.47 warrants escalated monitoring). Clinicians should prioritize sensitivity when early intervention is critical (e.g., ICU triage) and specificity when avoiding unnecessary resource allocation is paramount. We propose a stepped approach: (1) Biomarker values above the 50th percentile (e.g., RDW > 16.9%, RAR > 5.86) should prompt heightened vigilance, including more frequent physiological monitoring and early reassessment of organ support needs; (2) Values persistently elevated

after 48-72 hours of ICU care, especially when combined with rising MELD-Na scores, may indicate a high risk of irreversible deterioration, warranting discussions about goals of care and potential palliative consultation. Future studies should refine these thresholds and integrate them with established scores into decision-support tools. Notably, in our study cohort, the combination of RDW with SAPS II, MELD, and SOFA scores significantly improved the discriminative ability for mortality. While standalone AUCs for RDW-derived biomarkers were modest (≤63.7%), their incremental value when combined with established scores (AUC improvement up to 1.6%), cost-effectiveness, and ease of calculation support their role as adjunctive tools—not replacements—for existing prognostic systems. This may reflect RDW's dual role in reflecting both the inflammatory status (through iron metabolism disorders and impaired erythropoiesis) and nutritional/hematopoietic dysfunction in patients with cirrhosis.

This study had some limitations that should be acknowledged. First, owing to the retrospective nature of the study, improving the evidence level requires validation in prospective studies. Moreover, the present study is a single-center investigation. Despite its large sample size, the generalizability of the results similarly warrants further validation through multicenter studies. Our ICU-based cohort inherently represents the sickest cirrhosis patients, excluding those managed on wards or in

palliative care. While this limits generalizability to non-critical cirrhosis populations, it accurately reflects the target population for biomarker prognostication in critical settings. Second, our study focused on the initial RDW values and other related indicators measured on the first day of ICU admission. However, we have not yet investigated the prognostic impact of dynamic changes in RDW-related inflammatory biomarkers. Future research should focus more on the dynamic changes of indicators such as RDW to obtain more valuable prognostic information. Moreover, the absence of ~~granular~~ treatment-regimen data and specific causes of death in our dataset precludes any deeper ~~downstream~~ analyses. Lastly, the specific mechanisms and pathological states reflected by different RDW-related inflammatory biomarkers in patients with cirrhosis require further research for elucidation.

## *Conclusion*

In conclusion, our data suggest an association between RDW-related biomarkers and prognosis in ICU patients with cirrhosis. The RDW and HRR demonstrated superior predictive capabilities in this patient population. The integration of these indices into clinical practice, especially RDW and HRR, could enhance the prognostic assessment of ICU patients with cirrhosis, aiding in the identification of those at a higher risk of mortality and potentially guiding therapeutic interventions.

## Declarations

### Ethics approval and consent to participate

The MIMIC-IV database is a third-party anonymous public database approved by the Institutional Review Boards of Beth Israel Deaconess Medical Center (Boston, MA) and the Massachusetts Institute of Technology (Cambridge, MA). All procedures performed in the present study were in accordance with the principles outlined in the 1964 Helsinki Declaration and its later amendments. This study was exempted from obtaining informed consent by the institutional research committee of the First Hospital of Quanzhou Affiliated to Fujian Medical University (Quanzhou, China) because MIMIC-IV data is publicly available, and all patient data are de-identified.

### Consent for publication

Not Applicable.

### Data availability

The data is available in the MIMIC repository, https://mimic.physionet.org/

### Conflict of interest

The authors declare no competing interests.


### Funding

This research was funded by the Startup Fund for scientific studies at Fujian Medical University (Grant No. 2023QH1310) and the Quanzhou City Science & Technology Program in China (Grant No. 2024NY001). The funding sources had no role in the design of the study, collection and analysis of data, interpretation of results, and decision to publish.


### Author contributions

Xiaodong Zhu contributed to conceptualization, data extraction, and original writing. Xiaoqiang Liu and Chanchan Lin conducted formal analysis. The methodology, data curation, funding acquisition, writing review and editing were performed by Yingyi Li and Zicheng Huang. All authors have reviewed and approved the final manuscript.

**Acknowledgements**

We would like to express our gratitude to all the participants in the MIMIC databases. We would also like to express our special appreciation to Dr. Jie Liu from the Department of Vascular and Endovascular Surgery at the Chinese PLA General Hospital and Haibo Li from the Division of Birth Cohort Study at Fujian Maternity and Child Health Hospital for their thoughtful comments on the manuscript.

**Abbreviations**

| | |
|---|---|
| ALT | Alanine Aminotransferase |
| APS III | Acute Physiology Score III |
| AST | Aspartate Aminotransferase |
| AUC | Area Under the Curve |
| BMI | Body Mass Index |
| CI | Confidence Interval |
| DBP | Diastolic Blood Pressure |
| Hb | Hemoglobin |
| HBV | Hepatitis B Virus |
| HCC | Hepatocellular Carcinoma |
| HR | Hazard Ratio |
| HRR | Hemoglobin-to-Red Cell Distribution Width Ratio |
| ICU | Intensive Care Unit |
| INR | International Normalized Ratio |
| IQR | Interquartile Range |

| | |
|---|---|
| MBP | Mean Arterial Pressure |
| MIMIC-IV | Medical Information Mart for Intensive Care IV |
| PT | Prothrombin Time |
| RAR | Red Cell Distribution Width-to-Albumin Ratio |
| RDW | Red Cell Distribution Width |
| ROC | Receiver Operating Characteristic |
| RPR | Red Cell Distribution Width-to-Platelet Ratio |
| RRT | Renal Replacement Therapy |
| SBP | Systolic Blood Pressure |
| SpO2 | Pulse Oximetry |

**References**

1. Walter KL. What Is Cirrhosis? *JAMA*. 2023;330(4):386-386.
2. Piano S, Bunchorntavakul C, Marciano S, Rajender Reddy K. Infections in cirrhosis. *Lancet Gastroenterol Hepatol*. 2024;9(8):745-757.
3. Kosuta I, Premkumar M, Reddy KR. Review article: Evaluation and care of the critically ill patient with cirrhosis. *Aliment Pharmacol Ther*. 2024;59(12):1489-1509.
4. Li X, Wu J, Mao W. Evaluation of the neutrophil-to-lymphocyte ratio, monocyte-to-lymphocyte ratio, and red cell distribution width for the prediction of prognosis of patients with hepatitis B virus-related decompensated cirrhosis. *J Clin Lab Anal*. 2020;34(11):e23478.
5. Ephrem G. Red Blood Cell Distribution Width Should Indeed Be Assessed with Other Inflammatory Markers in Daily Clinical Practice. *Cardiology*. 2013;124(1):61-61.
6. Hong RH, Zhu J, Li ZZ, et al. Red blood cell distribution width is associated with neuronal damage in acute ischemic stroke. *Plant Pathol*. 2020;12(10):9855-9867.
7. Wu YC, Chen HH, Chao WC. Association between red blood cell distribution width and 30-day mortality in critically ill septic patients: a propensity score-matched study. *J Intensive Care*. 2024;12(1):34-43.
8. Wang J, Xu R. Red blood cell distribution width is an inflammatory but not a nutritional biomarker. *Clin Nutr*. 2024;43(4):1090-1091.
9. Zhang Q, Mao W, He X, Yuan M. High red cell distribution width-to-platelet ratio indicates adverse outcomes for hepatitis B virus-associated decompensated cirrhosis. *Biomark Med*. 2023;17(4):189-196.
10. Gialluisi A, Tirozzi A, Costanzo S, et al. Blood-based biological ageing and red

cell distribution width are associated with prevalent Parkinson’s disease: findings from a large Italian population cohort. *Front Endocrinol*. 2024;15:1376545-1376552.

11. Johnson AEW, Bulgarelli L, Shen L, et al. MIMIC-IV, a freely accessible electronic health record dataset. *Sci Data*. 2023;10(1):1-9.
12. Liu J, Huang X, Yue S, et al. Association of Red Cell Distribution Width-to-Platelet Ratio and Mortality in Patients with Sepsis. *Mediators Inflamm*. 2022;27(9):4915887-4915895.
13. Zhang W, Wang Y, Wang J, Wang S. Association between red blood cell distribution width and long-term mortality in acute respiratory failure patients. *Sci Rep*. 2020;10(1):21185-21196.
14. Yu Z, Zhang T, Shen J. Low Hemoglobin-to-Red Cell Distribution Width Ratio Is Associated with Mortality in Patients with HBV-Related Decompensated Cirrhosis. *Biomed Res Int*. 2022;14(2):5754790-5754796.
15. Turcato G, Campagnaro T, Bonora A, et al. Red blood cell distribution width independently predicts 1-month mortality in acute decompensation of cirrhotic patients admitted to emergency department. *Eur J Gastroenterol Hepatol*. 2017;30(1):33-38.
16. VanderWeele TJ, Ding P. Sensitivity Analysis in Observational Research: Introducing the E-Value. *Ann Intern Med*. 2017;167(4):268-274.
17. Chen W, Yu P, Chen C, et al. Association Between the Red Blood Cell Distribution Width and 30-Day Mortality in Intensive Care Patients Undergoing Cardiac Surgery: A Retrospective Observational Study Based on the Medical Information Mart for Intensive Care-IV Database. *Ann Lab Med*. 2024;44(5):401-409.
18. Aslam H, Oza F, Ahmed K, et al. The Role of Red Cell Distribution Width as a Prognostic Marker in Chronic Liver Disease: A Literature Review. *Int J Mol Sci*. 2023;24(4):3487-3501.
19. Michalak A, Guz M, Kozicka J, et al. Red blood cell distribution width derivatives in alcohol-related liver cirrhosis and metabolic-associated fatty liver disease. *World J Gastroenterol*. 2022;28(38):5636-5647.
20. Wang J, Huang R, Yan X, et al. Red blood cell distribution width: A promising index for evaluating the severity and long-term prognosis of hepatitis B virus-related diseases. *Dig Liver Dis*. 2020;52(4):440-446.
21. Wang LL, Wei TT, Yin JR, et al. Red blood cell distribution width and mean platelet volume are potential prognostic indices for patients with primary biliary cirrhosis. *Clin Chem Lab Med*. 2016;55(6):e127-e129.
22. Nawalerspanya S, Tantipisit J, Assawasuwannakit S, Kaewdech A, Chamroonkul N, Sripongpun P. Non-Invasive Serum Biomarkers for the Diagnosis of Cirrhosis in Patients with Autoimmune Hepatitis (AIH) and AIH-Primary Biliary Cholangitis Overlap Syndrome (AIH-PBC): Red Cell Distribution Width to Platelet Ratio (RPR) Yielded the Most Promising Result. *Diagnostics (Basel)*.

2024;14(3):265-277.

23. Zeng T, Yu J, Tan L, et al. Noninvasive indices for monitoring disease course in Chinese patients with autoimmune hepatitis. *Clin Chim Acta*. 2018;486:135-141.
24. Wang H, Wang J, Huang R, et al. Red blood cell distribution width for predicting significant liver inflammation in patients with autoimmune hepatitis. *Eur J Gastroenterol Hepatol*. 2019;31(12):1527-1532.
25. Taefi A, Huang CC, Kolli K, Ebrahimi S, Patel M. Red cell distribution width to platelet ratio, a useful indicator of liver fibrosis in chronic hepatitis patients. *Hepatol Int*. 2015;9(3):454-460.
26. Goyal H, Hu ZD. Prognostic value of red blood cell distribution width in hepatocellular carcinoma. *Ann Transl Med*. 2017;5(13):271-276.
27. Kai Y, Zishu G, Shihe G, Yufeng G, Qiang Z. Changes in Red Blood Cell Distribution Width is Associated with Liver Function Parameters and Prognosis in Patients with Chronic HBV Liver Disease. *Clin Lab*. 2017;62(11):2197-2202.
28. Xu W, Huo J, Chen G, et al. Association between red blood cell distribution width to albumin ratio and prognosis of patients with sepsis: A retrospective cohort study. *Front Nutr*. 2022;23(9):1019502-1019513.
29. Mao W, Yuan M, He X, Zhang Q. Red cell distribution width-to-albumin ratio is a predictor of survival in hepatitis B virus-associated decompensated cirrhosis. *Lab Med*. 2023;55(2):127-131.
30. Tan M, You R, Cai D, et al. The Red Cell Distribution Width to Albumin Ratio: A Novel Prognostic Indicator in Hepatitis B Virus-Related Hepatocellular Carcinoma. *Int J Med Sci*. 2024;22(2):441-450.
31. Chen YP, Hu XM, Liang XE, Huang LW, Zhu YF, Hou JL. Stepwise application of fibrosis index based on four factors, red cell distribution width-platelet ratio, and aspartate aminotransferase-platelet ratio for compensated hepatitis B fibrosis detection. *J Gastroenterol Hepatol*. 2017;33(1):256-263.
32. Milas GP, Karageorgiou V, Cholongitas E. Red cell distribution width to platelet ratio for liver fibrosis: a systematic review and meta-analysis of diagnostic accuracy. *Expert Rev Gastroenterol Hepatol*. 2019;13(9):877-891.
33. Dallio M, Romeo M, Vaia P, et al. Red cell distribution width/platelet ratio estimates the 3-year risk of decompensation in Metabolic Dysfunction-Associated Steatotic Liver Disease-induced cirrhosis. *World J Gastroenterol*. 2024;30(7):685-704.
34. Li Y, Xu C, Qin Z, Ge L. Relationship Between the Hemoglobin-to-Red Cell Distribution Width Ratio and in-Hospital Mortality in Patients with Chronic Heart Failure. *VHRM*. 2024;Volume 20:553-565.
35. Qin Z, Liao N, Lu X, Duan X, Zhou Q, Ge L. Relationship Between the Hemoglobin-to-Red Cell Distribution Width Ratio and All-Cause Mortality in Ischemic Stroke Patients with Atrial Fibrillation: An Analysis from the MIMIC-IV Database. *Neuropsychiatr Dis Treat*. 2022;18:341-354.

36. Wang J, Chen Z, Yang H, Li H, Chen R, Yu J. Relationship between the Hemoglobin-to-Red Cell Distribution Width Ratio and All-Cause Mortality in Septic Patients with Atrial Fibrillation: Based on Propensity Score Matching Method. *JCDD*. 2022;9(11):400-416.
37. Huang X, Yuan S, Ling Y, et al. The Hemoglobin-to-Red Cell Distribution Width Ratio to Predict All-Cause Mortality in Patients with Sepsis-Associated Encephalopathy in the MIMIC-IV Database. *Int J Clin Pract*. 2022;2022:1-10.
38. Michalak A, Guz M, Kozicka J, Cybulski M, Jeleniewicz W, Lach T, et al. Red blood cell distribution width derivatives in alcohol-related liver cirrhosis and metabolic-associated fatty liver disease. *World J Gastroenterol*. 2022;28:5636–47.
39. Lan W, Liu E, Sun D, Li W, Zhu J, Zhou J, et al. Red cell distribution in critically ill patients with chronic obstructive pulmonary disease. *Pulmonology*. 2022;30:34 – 42.
40. Wang P, Zhang Y, Xu W, Zheng Y, Jia L, He J, et al. Association between perioperative change in red cell distribution width and mortality in patients with brain tumor craniotomy. *J Clin Anesth*. 2024;97:111546 – 111546.
41. Seo YJ, Yu J, Park J-Y, Lee N, Lee J, Park JH, et al. Red cell distribution width/albumin ratio and 90-day mortality after burn surgery. *Burns Trauma*. 2022;1:1-10.
42. Hong J, Hu X, Liu W, Qian X, Jiang F, Xu Z, et al. Impact of red cell distribution width and red cell distribution width/albumin ratio on all-cause mortality in patients with type 2 diabetes and foot ulcers: a retrospective cohort study. *Cardiovasc Diabetol*. 2022;21(91):1-11.
43. Huo L, Zhao W, Ji X, Chen K, Liu T. The Combination Effect of the Red Blood Cell Distribution Width and Prognostic Nutrition Index on the Prognosis in Patients Undergoing PCI. *Nutrients*. 2024;16:3176 – 3176.

**Table 1. The basic demographics and clinical characteristics in patients with cirrhosis**

| Characteristics | MIMIC cohort (n = 1871) | Low RDW group (n = 924) | High RDW group (n = 947) | P-value |
|---|---|---|---|---|
| **Demographics** | | | | |
| Male, n (%) | 1212 (64.8) | 642 (69.5) | 570 (60.2) | < 0.001 |
| Age, years | 58.8 ± 12.3 | 60.1 ± 11.9 | 57.5 ± 12.5 | < 0.001 |
| Race (white), n (%) | 1185 (75.0) | 600 (75.9) | 585 (74.1) | 0.433 |
| BMI (kg/m$^2$) | 29.6 ± 7.8 | 29.4 ± 7.8 | 29.7 ± 7.7 | 0.557 |
| **Vital signs** | | | | |
| Heart rate (beats/min) | 88.5 ± 16.3 | 88.0 ± 16.8 | 88.9 ± 15.8 | 0.277 |
| SBP (mmHg) | 113.3 ± 16.0 | 115.4 ± 16.7 | 111.3 ± 14.9 | < 0.001 |
| DBP (mmHg) | 61.4 ± 10.2 | 62.6 ± 10.6 | 60.3 ± 9.7 | < 0.001 |
| MBP (mmHg) | 76.1 ± 10.7 | 77.5 ± 11.1 | 74.7 ± 10.1 | < 0.001 |
| Respiratory rate (beats/min) | 19.0 ± 4.2 | 19.0 ± 4.2 | 19.1 ± 4.3 | 0.816 |
| Temperature (℃) | 36.8 ± 0.5 | 36.9 ± 0.5 | 36.7 ± 0.5 | < 0.001 |
| Spo2 (%) | 96.9 ± 2.2 | 96.9 ± 2.1 | 96.9 ± 2.2 | 0.887 |
| **Comorbidities, n(%)** | | | | |
| Hypertension | 877 (46.9) | 460 (49.8) | 417 (44.0) | 0.013 |
| Diabetes | 532 (28.4) | 282 (30.5) | 250 (26.4) | 0.048 |
| Myocardial infarct | 133 (7.1) | 71 (7.7) | 62 (6.5) | 0.339 |
| Congestive heart failure | 297 (15.9) | 138 (14.9) | 159 (16.8) | 0.272 |
| Cerebrovascular disease | 128 (6.8) | 73 (7.9) | 55 (5.8) | 0.073 |
| Chronic pulmonary disease | 383 (20.5) | 208 (22.5) | 175 (18.5) | 0.031 |
| Renal disease | 360 (19.2) | 169 (18.3) | 191 (20.2) | 0.303 |
| Malignant cancer | 339 (18.1) | 203 (22.0) | 136 (14.4) | < 0.001 |
| Peptic ulcer disease | 151 (8.1) | 55 (6.0) | 96 (10.1) | < 0.001 |
| Malnutrition | 491 (26.2) | 194 (21.0) | 297 (31.4) | < 0.001 |
| **Clinical assessment and treatments, n (%)** | | | | |
| APS III | 68.9 ± 29.4 | 62.6 ± 28.7 | 75.0 ± 28.8 | < 0.001 |
| Charlson Comorbidity Index | 6.9 ± 2.7 | 6.8 ± 2.8 | 6.9 ± 2.7 | 0.957 |
| Etiology, alcohol cirrhosis | 1026 (54.8) | 455 (49.2) | 571 (60.3) | < 0.001 |
| Albumin use | 1253 (67.1) | 595 (64.5) | 658 (69.6) | 0.019 |
| Mechanical ventilation | 1085 (58.0) | 533 (57.7) | 552 (58.3) | 0.791 |
| Vasopressin | 790 (42.2) | 376 (40.7) | 414 (43.7) | 0.185 |
| RRT | 326 (17.4) | 112 (12.1) | 214 (22.6) | < 0.001 |
| **Laboratory Indicators** | | | | |
| RDW (%) | 17.4 ± 3.0 | 15.1 ± 1.1 | 19.7 ± 2.4 | < 0.001 |
| RAR | 6.2 ± 2.1 | 5.3 ± 1.4 | 7.1 ± 2.3 | < 0.001 |
| RPR | 0.2 (0.1, 0.3) | 0.1 (0.1, 0.2) | 0.2 (0.1, 0.3) | < 0.001 |

| | | | | |
|---|---|---|---|---|
| HRR | 0.6 ± 0.2 | 0.7 ± 0.2 | 0.5 ± 0.1 | < 0.001 |
| White blood cells ($10^9$/L) | 11.9 ± 7.9 | 11.4 ± 7.5 | 12.4 ± 8.3 | 0.008 |
| Hemoglobin (g/dL) | 9.6 ± 2.1 | 10.3 ± 2.1 | 8.9 ± 1.9 | < 0.001 |
| Platelet count ($10^9$/L) | 100.0 (65.5, 151.5) | 108.0 (72.8, 161.0) | 90.0 (60.0, 143.5) | < 0.001 |
| Total bilirubin (mg/dL) | 3.3 (1.5, 8.5) | 2.2 (1.1, 4.4) | 5.5 (2.3, 13.6) | < 0.001 |
| Asparate aminotransferase (U/L) | 78.0 (43.0, 212.0) | 73.5 (41.0, 245.8) | 82.0 (45.0, 191.5) | 0.370 |
| Alanine aminotransferase (U/L) | 37.0 (21.0, 96.5) | 41.0 (21.0, 117.0) | 36.0 (21.0, 80.5) | 0.019 |
| Albumin (g/dL) | 3.0 ± 0.7 | 3.0 ± 0.6 | 2.9 ± 0.7 | 0.410 |
| Creatinine (mg/dL) | 1.2 (0.8, 2.2) | 1.1 (0.8, 1.9) | 1.4 (0.8, 2.6) | < 0.001 |
| Urea nitrogen (mg/dL) | 27.0 (16.0, 49.0) | 22.5 (14.0, 39.0) | 32.0 (18.0, 55.0) | < 0.001 |
| Glucose (mmol/L) | 150.9 ± 89.4 | 157.7 ± 87.8 | 144.3 ± 90.4 | 0.001 |
| Sodium (mmol/L) | 136.4 ± 6.8 | 136.6 ± 6.9 | 136.1 ± 6.7 | 0.140 |
| Potassium (mmol/L) | 4.3 ± 0.9 | 4.3 ± 0.8 | 4.3 ± 0.9 | 0.306 |
| Chloride (mmol/L) | 102.0 ± 7.8 | 102.4 ± 7.7 | 101.6 ± 8.0 | 0.046 |
| Calcium (mmol/L) | 8.4 ± 1.1 | 8.3 ± 1.1 | 8.4 ± 1.1 | 0.315 |
| Anion gap (mmol/L) | 16.5 ± 6.2 | 16.2 ± 6.3 | 16.9 ± 6.1 | 0.040 |
| Lactate (mmol/L) | 2.6 (1.7, 4.3) | 2.4 (1.6, 4.1) | 2.7 (1.8, 4.4) | 0.001 |
| **Outcome**, n (%) | | | | |
| In-hospital mortality | 499 (26.7) | 179 (19.4) | 320 (33.8) | < 0.001 |
| 90-day mortality | 736 (39.3) | 276 (29.9) | 460 (48.6) | < 0.001 |
| 365-day mortality | 898 (48.0) | 357 (38.6) | 541 (57.1) | < 0.001 |
| Variceal bleeding | 221 (11.8) | 82 (8.9) | 139 (14.7) | < 0.001 |
| Ascites | 972 (52.0) | 385 (41.7) | 587 (62.0) | < 0.001 |
| Hepatorenal syndrome | 322 (17.2) | 103 (11.1) | 219 (23.1) | < 0.001 |
| Hepatic encephalopathy | 190 (10.2) | 79 (8.5) | 111 (11.7) | 0.023 |
| Spontaneous peritonitis | 220 (11.8) | 73 (7.9) | 147 (15.5) | < 0.001 |

BMI, body mass index; SBP, systolic blood pressure; DBP, diastolic blood pressure; MBP, mean arterial pressure; $SpO_2$, pulse oximetry; APS III, Acute Physiology Score III; RRT, renal replacement therapy; RDW, red cell distribution width; RAR, red cell distribution width-to-albumin ratio; RPR, red cell distribution width-to-platelet ratio; HRR, hemoglobin-to-red cell distribution width.

**Table 2. The association of red cell distribution width and prognosis of patients with cirrhosis**

| Outcome | Unadjusted Model | | Adjusted Model[a] | |
|---|---|---|---|---|
| | HR (95% CI) | P value | HR (95% CI) | P value |
| **In-hospital mortality** | | | | |
| RDW | 1.14 (1.11~1.16) | <0.001 | 1.14 (1.10~1.19) | <0.001 |
| RAR | 1.10 (1.07~1.12) | <0.001 | 1.10 (1.03~1.17) | 0.003 |
| RPR | 2.83 (1.97~4.06) | <0.001 | 2.60 (1.37~4.94) | 0.003 |
| HRR | 0.13 (0.07~0.22) | <0.001 | 0.01 (0.00~0.04) | <0.001 |
| **90-day mortality** | | | | |
| RDW | 1.13 (1.1~1.15) | <0.001 | 1.14 (1.10~1.18) | <0.001 |
| RAR | 1.10 (1.08~1.12) | <0.001 | 1.11 (1.05~1.17) | <0.001 |
| RPR | 2.43 (1.77~3.34) | <0.001 | 2.30 (1.29~4.08) | 0.005 |
| HRR | 0.15 (0.1~0.23) | <0.001 | 0.01 (0.00~0.03) | <0.001 |
| **365-day mortality** | | | | |
| RDW | 1.11 (1.09~1.13) | <0.001 | 1.14 (1.10~1.18) | <0.001 |
| RAR | 1.10 (1.08~1.12) | <0.001 | 1.10 (1.05~1.16) | <0.001 |
| RPR | 2.24 (1.66~3.03) | <0.001 | 2.23 (1.29~3.83) | 0.004 |
| HRR | 0.18 (0.12~0.26) | <0.001 | 0.01 (0.00~0.03) | <0.001 |

RDW, red cell distribution width; RAR, red cell distribution width-to-albumin ratio; RPR, red cell distribution width-to-platelet ratio; HRR, hemoglobin-to-red cell distribution width; MIMIC-IV, Medical Information Mart for Intensive Care IV;HR: hazard ratio; CI: confidence interval.

a: Models of RDW and RPR were adjusted for sex, age, race, BMI, Charlson Comorbidity Index, vasopressin use, albumin use, mechanical ventilation, renal replacement therapy, hemoglobin, hematocrit, albumin, potassium, lactate; Model of RAR was adjusted for sex, age, race, BMI, Charlson Comorbidity Index, vasopressin use, albumin use, mechanical ventilation, renal replacement therapy, hemoglobin, hematocrit, potassium, lactate; Model of HRR was adjusted for sex, age, race, BMI, Charlson comorbidity index, vasopressin use, albumin use, mechanical ventilation, renal replacement therapy, hematocrit, albumin, potassium, lactate.

**Table 3. Information of receiver operating characteristic curves in Fig. 3.**

| Variables | AUC (%) | 95% CI (%) | Threshold | Sensitivity | Specificity |
|---|---|---|---|---|---|
| **In-hospital mortality** | | | | | |
| RDW | 63.22 | 60.37~66.08 | 16.75 | 0.661 | 0.536 |
| RAR | 59.75 | 56.82~62.68 | 6.39 | 0.491 | 0.652 |
| RPR | 55.95 | 52.89~59.02 | 0.19 | 0.523 | 0.596 |
| HRR | 62.18 | 59.27~65.09 | 0.47 | 0.473 | 0.730 |
| **90-day mortality** | | | | | |
| RDW | 63.66 | 61.11~66.21 | 17.55 | 0.538 | 0.667 |
| RAR | 59.87 | 57.23~62.51 | 6.44 | 0.465 | 0.686 |
| RPR | 54.29 | 51.56~57.01 | 0.19 | 0.493 | 0.602 |
| HRR | 62.43 | 59.84~65.01 | 0.56 | 0.644 | 0.560 |
| **365-day mortality** | | | | | |
| RDW | 62.48 | 59.96~64.99 | 16.75 | 0.617 | 0.577 |
| RAR | 59.49 | 56.92~62.05 | 5.86 | 0.576 | 0.571 |
| RPR | 54.55 | 51.93~57.17 | 0.19 | 0.490 | 0.615 |
| HRR | 61.82 | 59.28~64.35 | 0.56 | 0.624 | 0.576 |

ROC, receiver operating characteristic curve; AUC, area under the curve; CI, confidence interval; RDW, red cell distribution width; RAR, red cell distribution width-to-albumin ratio; RPR, red cell distribution width-to-platelet ratio; HRR, hemoglobin-to-red cell distribution width.

**Table 4. Comparison of Predictive Abilities of APS III, SAPS II, MELD, SOFA Scores and Their Combined Models with RDW**

| In-hospital mortality | AUC (%) | 95% CI (%) | Sensitivity | Specificity | P value |
|---|---|---|---|---|---|
| **APS III** | 80.23 | 78.05~82.41 | 0.676 | 0.784 | 0.058 |
| APS III + RDW | 80.86 | 78.71~83.00 | 0.709 | 0.764 | |
| **SAPS II** | 75.37 | 72.91~77.83 | 0.647 | 0.752 | 0.002 |
| SAPS II + RDW | 76.97 | 74.63~79.32 | 0.730 | 0.661 | |
| **MELD** | 74.60 | 72.07~77.14 | 0.734 | 0.665 | 0.037 |
| MELD + RDW | 75.21 | 72.69~77.73 | 0.663 | 0.752 | |
| **SOFA** | 74.28 | 71.71~76.85 | 0.856 | 0.489 | 0.002 |
| SOFA + RDW | 75.55 | 73.01~78.09 | 0.699 | 0.685 | |

ROC, receiver operating characteristic curve; AUC, area under the curve; CI, confidence interval; RDW, red cell distribution width; RAR, red cell distribution width-to-albumin ratio; RPR, red cell distribution width-to-platelet ratio; HRR, hemoglobin-to-red cell distribution width; APS III, Acute Physiology and Chronic Health Evaluation III; SAPS II: Simplified Acute Physiology Score II; MELD: Model for End-Stage Liver Disease; SOFA: Sequential Organ Failure Assessment.

**Fig1. Flow of the study**

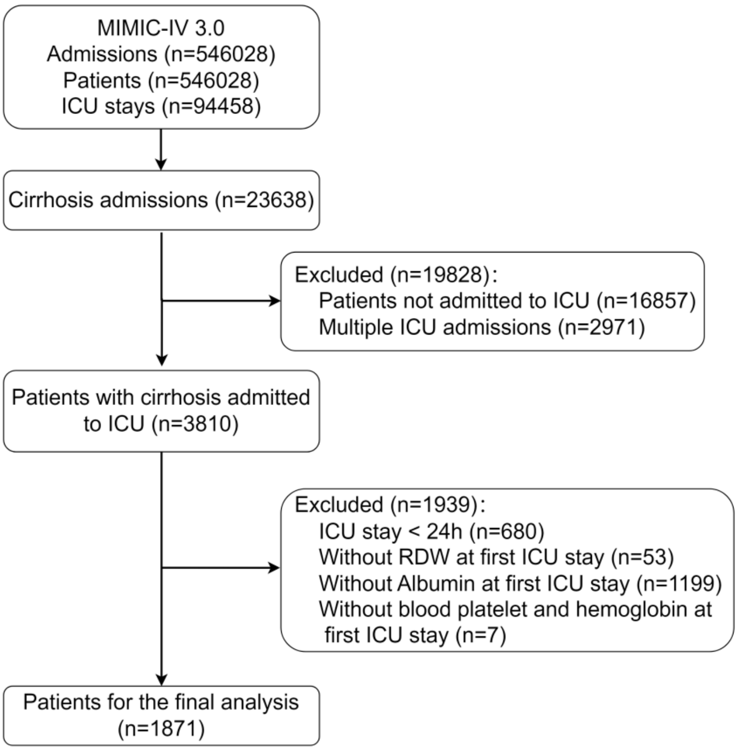


RDW, red cell distribution width; MIMIC-IV, Medical Information Mart for Intensive Care IV; ICU, Intensive Care Unit

**Fig2. Analysis of the association between RAW-derived indices and in-hospital mortality.**

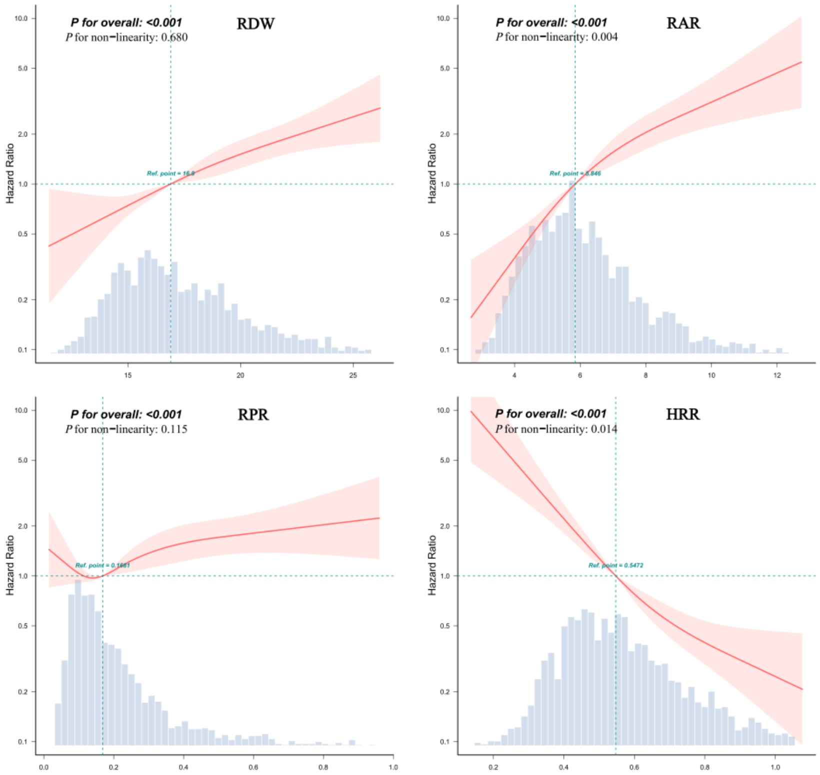


Data were fitted using Cox proportional hazards regression models based on restricted cubic splines, with each RDW-derived index entered as a continuous variable. Models were adjusted for the same covariates as in the fully adjusted models (Models for RDW and RPR were adjusted for sex, age, race, BMI, Charlson Comorbidity Index, vasopressin use, albumin use, mechanical ventilation, renal replacement therapy, hemoglobin, hematocrit, albumin, potassium, and lactate; the RAR model was adjusted for sex, age, race, BMI, Charlson Comorbidity Index, vasopressin use, albumin use, mechanical ventilation, renal replacement therapy, hemoglobin, hematocrit, potassium, and lactate; the HRR model was adjusted for sex, age, race, BMI, Charlson Comorbidity Index, vasopressin use, albumin use, mechanical ventilation, renal replacement therapy, hematocrit, albumin, potassium, and lactate). The gray shaded areas represent 95% confidence intervals.

RDW, red cell distribution width; RAR, red cell distribution width-to-albumin ratio; RPR, red cell distribution width-to-platelet ratio; HRR, hemoglobin-to-red cell distribution width.

**Fig3. ROC analyses of predictors of RDW, RAR, RPR, and HRR for in-hospital mortality, 90-day mortality and 365-day mortality in patients with cirrhosis**

(A) RDW for in-hospital mortality in cirrhosis patients, compared with RAR, RPR, HRR, APS III, SAPS II, MELD, and SOFA scores; (B) RDW for 90-day mortality in cirrhosis patients, compared with RAR, RPR, HRR, APS III, SAPS II, MELD, and SOFA scores; (C) RDW for 365-day mortality in cirrhosis patients, compared with RAR, RPR, HRR, APS III, SAPS II, MELD, and SOFA scores;

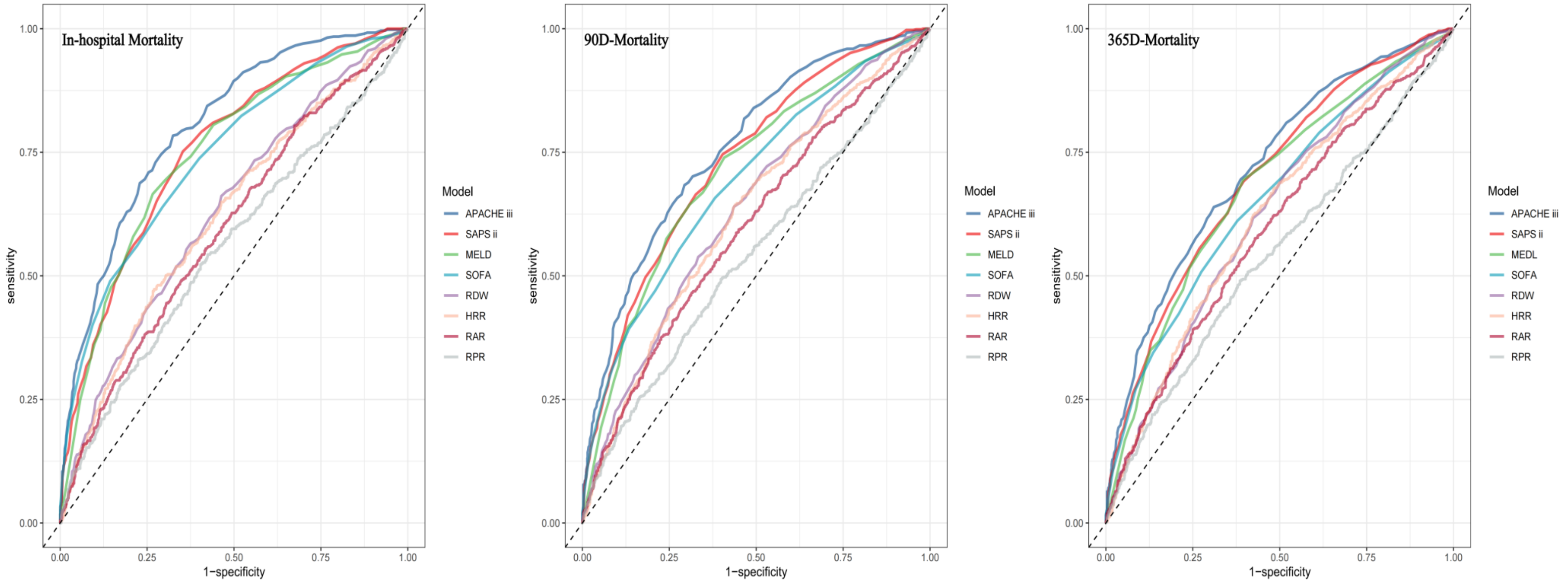


RDW, red cell distribution width; RAR, red cell distribution width-to-albumin ratio; RPR, red cell distribution width-to-platelet ratio; HRR, hemoglobin-to-red cell distribution width; MIMIC-IV, Medical Information Mart for Intensive Care IV. APS III, Acute Physiology and Chronic Health Evaluation III; SAPS II: Simplified Acute Physiology Score II; MELD: Model for End-Stage Liver Disease; SOFA: Sequential Organ Failure Assessment.

Click here to access/download
**Supporting Information**
Supplementary materials.docx
