# Supplementary material for: Association between Red Cell Distribution Width (RDW)-related inflammatory biomarkers and prognosis in ICU Cirrhosis patients: Evidence from the MIMIC-IV
Source: PLoS One. 2026 Jun 26;21(6):e0352023. doi: 10.1371/journal.pone.0352023 (PMC13308776; doi:10.1371/journal.pone.0352023)
Supplement: Attachment — Submitted filename: Reviewer Report PONE_D_25_67953.docx [file pone.0352023.s002.docx]

Reviewer Report – PLOS ONE

Manuscript ID: PONE-D-25-67953

Title: Association between Red Cell Distribution Width (RDW)-Related Inflammatory Biomarkers and Prognosis in ICU Cirrhosis Patients: Evidence from MIMIC-IV

General Assessment

This manuscript presents a retrospective cohort study evaluating the prognostic value of the red cell distribution width (RDW) and related biomarkers (RDW-to-albumin ratio (RAR), RDW-to-platelet ratio (RPR), and

haemoglobin-to-RDW ratio (HRR)) in ICU patients with cirrhosis using the MIMIC-IV database. The topic is clinically relevant, as prognostic stratification in critically ill cirrhosis patients remains a major challenge. The use of a large, well-established database and multiple mortality endpoints (in-hospital, 90-day, 365-day) strengthens the study.

The authors demonstrate statistically significant associations between RDW-related indices and mortality outcomes, with RDW and HRR showing the strongest predictive value. However, while the study is methodologically comprehensive, there are several concerns regarding interpretation, clinical relevance, and presentation that should be addressed before the manuscript can be considered for publication.

Major Comments

1. Clinical significance vs. Statistical significance

Although associations are statistically significant, the predictive performance is modest (AUC ~0.55–0.64).

The incremental improvement when combined with established scores (SAPS II, MELD, SOFA) is minimal (0.6–1.6% AUC increase), raising concerns about real-world clinical utility. The manuscript should more clearly justify how these biomarkers would change clinical decision-making.

Recommendation: Expand discussion on clinical applicability, including thresholds, decision pathways, or specific ICU use-cases.

2. Additional confounders

Important ICU variables may still be missing (e.g., infection status, vasopressor dose, liver-specific complications severity).

Recommendation: Provide a full list of adjusted covariates in the main manuscript (not only supplementary).

Discuss potential residual confounding more explicitly.

3. Lack of external validation

The study is based on a single-centre database (MIMIC-IV), which limits generalisability.

Recommendation: Emphasize this limitation more strongly.

4. Biomarker timing and dynamics

Only baseline (first 24h ICU) values were used. Dynamic changes in RDW and related indices may be more prognostically informative.

Recommendation: Discuss the limitation more critically. Suggest longitudinal analyses as a key next step.

5. Overstatement in conclusions

The conclusion suggests these biomarkers may “optimise patient management and resource allocation,” which is not directly demonstrated.

Recommendation: Tone down conclusions to reflect associative findings, not clinical impact.

Minor Comments

1. Language and grammar

Several grammatical issues and awkward phrasing are present throughout the manuscript.

Examples:

“patients with cirrhotic in the ICU” → should be “patients with cirrhosis in the ICU”

Inconsistent tense usage

Recommendation: Professional English editing is required.

2. Redundancy

The manuscript contains repetition, especially in:

Introduction (background on RDW and cirrhosis)

Discussion (reiteration of known literature)

Recommendation: Condense for clarity and readability.

3. ROC Interpretation

The ROC results are presented as “predictive,” but discrimination is only modest.

Recommendation: Use more cautious wording (e.g., “limited discrimination”).

4. Figures and Tables

Ensure:

Clear labeling of ROC curves

Confidence intervals included where appropriate

Better readability of supplementary materials

5. Ethical Statement Section

In the submission system, ethics is marked “N/A,” but later described appropriately in the manuscript.

Recommendation: Ensure consistency across submission fields and manuscript text.

Strengths

Large sample size (n = 1,871 ICU cirrhosis patients)

Use of a high-quality, widely recognised database (MIMIC-IV)

Inclusion of multiple clinically relevant mortality endpoints

Comprehensive statistical approach

Exploration of multiple RDW-derived biomarkers rather than RDW alone

Recommendation: Major Revision

The study addresses an important clinical question and has a solid methodological foundation. However, concerns regarding clinical relevance, interpretation of effect sizes, and overstatement of conclusions must be addressed before the manuscript is suitable for publication.
