# Supplementary material for: Association between Red Cell Distribution Width (RDW)-related inflammatory biomarkers and prognosis in ICU Cirrhosis patients: Evidence from the MIMIC-IV
Source: PLoS One. 2026 Jun 26;21(6):e0352023. doi: 10.1371/journal.pone.0352023 (PMC13308776; doi:10.1371/journal.pone.0352023)
Supplement: Attachment — Submitted filename: Response to Reviewers.docx [file pone.0352023.s004.docx]

PONE-D-25-67953

Association between Red Cell Distribution Width (RDW)-Related Inflammatory Biomarkers and Prognosis in ICU Cirrhosis Patients: Evidence from MIMIC-IV

PLOS One

Dear editor Elizabeth S. Mayne and reviewers,

We sincerely thank the academic editor and the two reviewers for their thorough, constructive, and insightful suggestions. We have carefully considered all the recommendations and have undertaken substantial revisions to the manuscript. The two most important methodological refinements are as follows: (1) the primary short-term endpoint was changed from in-hospital mortality to 30-day mortality, thereby eliminating the issues arising from competing risk and informative censoring; and (2) Z-score standardization was adopted, with both unit hazard ratios and per-standard-deviation hazard ratios reported, thereby addressing the inherent scaling and interpretability challenges of ratio-derived biomarkers. All results have been reanalyzed using the updated endpoints and incorporating the standardized indices.

Below, we provide a point-by-point response to each comment raised by the editor, the journal requirements, and the two reviewers. All line and page numbers refer to the revised manuscript. Modifications made in response to specific comments have been highlighted in yellow in the accompanying "Revised Manuscript with Tracked Changes" file.

**EDITOR COMMENTS**

**Editor Comment 1: Frequency of testing (only baseline data)**

**Comment:** *"Both reviewers found this interesting but there were concerns about: The frequency of testing (only baseline data)."*

**Response:** We fully acknowledge this important limitation. Our study exclusively used biomarker values measured within the first 24 hours of ICU admission, which precludes assessment of whether dynamic changes in RDW-related indices during the ICU stay provide additional prognostic information beyond baseline values. This limitation has now been substantially expanded in the Discussion.In the Discussion, we have added: "Fourth, we examined only baseline biomarker values measured within the first 24 hours of ICU admission. Dynamic changes during the ICU stay — particularly within the first 30 days — may capture evolving inflammatory status and treatment response beyond what static measurements can offer. Future studies should evaluate longitudinal RDW trajectories and delta-RDW (change from baseline) to determine whether time-varying biomarker values enhance predictive performance." We have also added this caveat to the Abstract. (Page 23-24, Line 486-493)

**Editor Comment 2: Missing laboratory parameters**

**Comment:** *"Both reviewers found this interesting but there were concerns about: Missing laboratory parameters known to be important in outcomes in ICU mortality."*

**Response:** We agree that important ICU variables were not fully captured in our models, potentially resulting in residual confounding. We have addressed this in three ways: (1) explicitly discussing residual confounding in the Discussion; (2) reporting E-values (both per-unit and per-SD) to assess robustness to unmeasured confounding (Supplementary Table 2); and (3) providing a comprehensive list of all adjusted covariates in the Methods. In the Discussion, we have added: “Fifth, important ICU variables such as documented infection status, specific vasopressor dosing regimens, and granular severity assessments of liver-specific complications were not fully captured in our models, potentially resulting in residual confounding” (Page 24, Line494-497). The E-values reported in Supplementary Table 5 provide quantitative assessment of how strong an unmeasured confounder would need to be to nullify our findings (Page 16, Line 326-330). In the Methods, we now explicitly list all covariates included in the fully adjusted model (Page 10, Line 192-199).

**Editor Comment 3: Overstatement of clinical extrapolation**

**Comment:** *"Both reviewers found this interesting but there were concerns about: Overstatement of clinical extrapolation — the AUC was showed mild predictive value and both reviewers felt that this would not translate directly to clinical significance."*

**Response:** We completely agree with this critique. The original manuscript overstated the clinical utility of RDW-related biomarkers given their modest standalone discrimination. We have comprehensively revised the tone throughout the manuscript, removing all unsupported claims and replacing them with appropriately cautious, evidence-based statements. (1) Abstract: The Conclusion now states: "RDW-related biomarkers demonstrate statistically significant but modest associations with mortality in ICU cirrhosis patients. RDW and HRR showed the strongest predictive capabilities among the four indices, though standalone discrimination was limited. These accessible, cost-effective biomarkers may serve as adjunctive prognostic markers when combined with established scoring systems. However, further prospective validation is required before clinical implementation, and dynamic changes in these biomarkers warrant investigation in future studies." (Page 3 Line44-52). (2) Discussion: We have added: " While the standalone AUCs for RDW-derived biomarkers were modest (≤63.7%), their incremental value when combined with established scores (AUC improvement 0.68–1.6%), combined with their cost-effectiveness and universal availability in routine laboratories, supports their potential role as adjunctive tools — not replacements — for existing prognostic systems. ...... The clinical impact of these AUC improvements remains to be established through prospective validation. " (Page 22, Line 449-460). (3) Conclusion: All claims about "optimising patient management and resource allocation" have been removed (Page 24-25, Line505-514).

**JOURNAL REQUIREMENTS**

**Requirement 1: PLOS ONE style requirements**

**Comment:** *"Please ensure that your manuscript meets PLOS ONE's style requirements, including those for file naming."*

**Response:** We have carefully reviewed the PLOS ONE formatting guidelines and templates, and have ensured that the revised manuscript adheres to all style requirements, including font, spacing, heading formats, and figure/table numbering conventions.

**Requirement 2: Remove funding-related text from manuscript**

**Comment:** *"Please remove any funding-related text from the manuscript. Funding information should not appear in any section or other areas of your manuscript."*

**Response:** We have removed all funding-related text from the manuscript body. The Funding section in the Declarations has been replaced with "This research received no specific grant from any funding agency." All funding details now appear only in the Funding Statement section of the online submission form, as required.

**Requirement 3: Data Availability Statement (missing DOI/accession number)**

**Comment:** *"Please note that your Data Availability Statement is currently missing the repository name and/or the DOI/accession number of each dataset."*

**Response:** We thank the Editor for this important correction. The data underlying this study are from the Medical Information Mart for Intensive Care IV (MIMIC-IV) database, version 3.0, available through PhysioNet ([https://physionet.org/content/mimiciv/3.0/](https://physionet.org/content/mimiciv/3.0/)).

**Requirement 4: Ethics statement location**

**Comment:** *"Your ethics statement should only appear in the Methods section of your manuscript. If your ethics statement is written in any section besides the Methods, please move it to the Methods section and delete it from any other section."*

**Response:** We have removed the Ethics Statement from the Declarations section. The ethics information now appears exclusively in the Methods section (under "Data"), as required (Page 6, Line 104-111).

**Requirement 5: Supporting Information captions**

**Comment:** *"Please include captions for your Supporting Information files at the end of your manuscript, and update any in-text citations to match accordingly."*

**Response:** We have added complete captions for all Supporting Information files at the end of the revised manuscript and have updated all in-text citations to ensure consistency (Page 25).

**REVIEWER #1 (Dr. Jody Rusch)**

We sincerely thank Dr. Rusch for the detailed and methodologically rigorous review. The comments on scaling, collinearity, and endpoint selection have been particularly influential in shaping our revisions. Below, we address each comment in detail.

**Major Comment 1: Interpretation and Scaling of Ratio Variables (Especially HRR)**

**Comment:** *"The adjusted hazard ratios reported for HRR (~0.01 across endpoints) imply an implausibly large protective effect per unit increase. Given the narrow expected distribution of HRR, this likely reflects scaling or collinearity issues rather than a true biological magnitude of effect. Hazard ratios should be reported per standard deviation or per interquartile range to improve interpretability. Additionally, variance inflation factor (VIF) analysis or other collinearity diagnostics should be provided. Without clarification, the HRR results are difficult to interpret and potentially misleading."*

**Response:** We fully agree with this critical methodological concern. The per-unit HR for HRR (HR 0.01) is indeed misleading because HRR has a very narrow distribution (mean 0.6, SD 0.2), where a "one-unit increase" represents a change from a typical value to one 3-5 SDs above the mean -- not a clinically meaningful increment. To resolve this, we have implemented a comprehensive z-score standardisation approach with dual HR reporting: (1) Z-score standardisation: Each biomarker was converted to a z-score (z = [value - mean] / SD) using the cohort mean and standard deviation. Parallel Cox models were fitted using both original-scale variables (yielding per-unit HRs) and z-scored variables (yielding per-SD HRs) (Page 7-8, Line 133-139). (2) Dual reporting in Table 2: The revised Table 2 now presents both per-unit HRs (for comparability with prior literature) and per-SD HRs (for clinically interpretable cross-biomarker comparisons). The per-SD HRs for 30-day mortality are: RDW (HR 1.41, 95% CI 1.30-1.52), RAR (HR 1.17, 95% CI 1.11-1.24), RPR (HR 1.19, 95% CI 1.11-1.28), and HRR (HR 0.52, 95% CI 0.44-0.61) (Page 13, Line 264-269). The per-SD HRR of 0.52 (48% risk reduction per SD) is clinically interpretable and consistent with inflammatory biomarker literature. In addition, we provide the VIF analysis results in the table below. All covariates in the fully adjusted model show no collinearity with the RDW-related indicators. As shown in the table, except for hemoglobin and hematocrit, the VIF values of all other covariates are less than 5. Hemoglobin and hematocrit have a VIF greater than 5 due to collinearity between them, but neither exhibits collinearity with RDW, RAR, or RPR. In the adjustment of the HRR model, the hemoglobin variable has been excluded, and the VIF of hematocrit is less than 5, indicating no collinearity with HRR.

**VIF analysis results:**

**Major Comment 2: Potential Overadjustment and Collinearity**

**Comment:** *"The fully adjusted models include laboratory variables that are components of the derived indices (e.g., hemoglobin in models evaluating HRR; albumin and platelets in models evaluating RAR and RPR). This raises concerns regarding collinearity, overadjustment, and instability of regression coefficients. Sensitivity analyses excluding component variables from adjustment models would strengthen confidence in the reported associations."*

**Response:** Thank you for your suggestion. In the Methods section, we have listed all covariates included in the fully adjusted models. For each model, we have excluded the laboratory variables that constitute the derived indices (e.g., hemoglobin was excluded in the model assessing HRR; albumin was excluded in the model assessing RAR). Therefore, issues such as collinearity, over-adjustment, or instability of regression coefficients resulting from this problem do not exist. (Page 10, Line 192-199)

**Major Comment 3: Modeling of In-Hospital Mortality**

**Comment:** *"In-hospital mortality is analyzed using Cox regression without detailed description of time origin, censoring assumptions, or consideration of discharge alive as a competing event. Because discharge alive precludes subsequent in-hospital death, this is technically a competing risks scenario. At minimum, the authors should clarify the time-to-event structure and justify the use of standard Cox regression."*

**Response:** We thank Dr. Rusch for raising this critical methodological concern. Upon careful reflection, we agree that in-hospital mortality presents fundamental statistical challenges that are difficult to resolve satisfactorily: (i) hospital length of stay varies widely, creating differential observation periods; (ii) discharge alive constitutes a competing event that precludes subsequent in-hospital death; and (iii) discharge is non-random with respect to prognosis, violating the non-informative censoring assumption. Rather than applying competing risks regression (Fine-Gray) to an endpoint where the competing event (discharge) is partially determined by the biomarkers themselves, we have taken the more rigorous approach of changing the primary short-term endpoint from in-hospital mortality to 30-day mortality. (1) Endpoint change: The primary short-term endpoint has been changed from in-hospital mortality to 30-day mortality. (2) All results re-calculated: Tables 2-4 and Figures 1-3 have been completely re-analysed using the 30-day endpoint. The revised Results now report 30-day mortality as the primary endpoint, with 90-day and 365-day mortality as secondary endpoints.

**Major Comment 4: Modest Predictive Performance and Overstatement of Clinical Utility**

**Comment:** *"The reported AUC values for RDW and derived indices range from approximately 0.54 to 0.64, indicating weak to moderate discrimination. Although incremental AUC improvements when adding RDW to SAPS II, MELD, or SOFA are statistically significant, they are small (<0.02). The clinical relevance of such marginal improvements is uncertain (likely negligible). Accordingly, statements suggesting that these biomarkers "enhance clinical decision-making" or "optimize resource allocation" should be tempered."*

**Response:** We completely agree with this assessment. The original manuscript overstated the clinical utility of these biomarkers. We have comprehensively revised the Discussion and Conclusion to adopt appropriately cautious language that accurately reflects the modest predictive performance and uncertain clinical relevance of marginal AUC improvements. (1) Discussion (revised): "While the standalone AUCs for RDW-derived biomarkers were modest (≤63.7%), their incremental value when combined with established scores (AUC improvement 0.68–1.6%), combined with their cost-effectiveness and universal availability in routine laboratories, supports their potential role as adjunctive tools — not replacements — for existing prognostic systems." (Page 22, Line 449-454) (2) Conclusion (revised): "In conclusion, our data suggest that RDW-related biomarkers are independently associated with 30-day mortality in ICU patients with cirrhosis. RDW and HRR demonstrated the strongest per-SD associations among the four indices, though standalone discrimination was modest. The integration of these indices into clinical practice could provide modest additional prognostic information when combined with established scoring systems. Further prospective validation, external validation, and investigation of dynamic biomarker changes are required before these findings can inform clinical decision-making." (Page 24-25, Line 505-514) (3) All unsupported claims removed: Phrases such as "enhance clinical decision-making", "optimise patient management", and "optimise resource allocation" have been completely removed from the Abstract, Discussion, and Conclusion.

**Major Comment 5: Cohort Derivation and Missing Data**

**Comment:** *"Patients without RDW or derived index measurements within 24 hours were excluded. The manuscript does not report: The number excluded for missing laboratory values; A flow diagram of cohort derivation; Comparison of included vs excluded patients. Given that laboratory testing intensity in ICU populations correlates with illness severity, exclusion based on missing laboratory data may introduce selection bias. Greater transparency in cohort derivation is required."*

**Response:** We completely agree that transparency in cohort derivation is essential. We have addressed this in three ways: (1) reporting the number of patients excluded at each step; and (2) adding Supplementary Table 3 comparing baseline characteristics between included and excluded patients to assess potential selection bias.

**Minor Comments (Reviewer #1)**

**Response:** All minor comments have been addressed as follows:

(1) Restricted cubic splines: The Methods now explicitly describes the use of restricted cubic spline functions to assess non-linear relationships, supplementing the median-dichotomised Kaplan-Meier analyses. RCS results are presented in Fig. 2, Fig. S4, Fig. S5.
(2) Limitations expanded: The Limitations section now explicitly acknowledges: (a) modest discrimination; (b) lack of external validation. Potential collinearity and scaling concerns has been addressed via our per-SD reporting and VIF diagnostics.
(3) Laboratory units clarified: All laboratory units have been explicitly specified in Table 1 (e.g., albumin in g/dL, creatinine in mg/dL, glucose in mmol/L).
(4) Subgroup analyses: We used the likelihood ratio test to assess interactions. Although the p-values for interactions between BMI and multiple RDW-related indices were below 0.05, considering the impact of multiple testing, a p-value of less than 0.05 for BMI interactions may not be statistically significant.

**REVIEWER #2 (Anonymous)**

We sincerely thank Reviewer #2 for the thorough and constructive feedback on clinical significance, confounders, external validation, biomarker dynamics, and conclusion wording. These comments have significantly strengthened the manuscript.

**Major Comment 1: Clinical Significance vs. Statistical Significance**

**Comment:** *"Although associations are statistically significant, the predictive performance is modest (AUC ~0.55-0.64). The incremental improvement when combined with established scores (SAPS II, MELD, SOFA) is minimal (0.6-1.6% AUC increase), raising concerns about real-world clinical utility. The manuscript should more clearly justify how these biomarkers would change clinical decision-making. Recommendation: Expand discussion on clinical applicability, including thresholds, decision pathways, or specific ICU use-cases."*

**Response:** We completely agree. The original manuscript conflated statistical significance with clinical significance. We have comprehensively revised the Discussion to clearly distinguish between these concepts and provide a more nuanced assessment of clinical applicability. (1) Discussion (revised): "While the standalone AUCs for RDW-derived biomarkers were modest (≤63.7%), their incremental value when combined with established scores (AUC improvement 0.68–1.6%), combined with their cost-effectiveness and universal availability in routine laboratories, supports their potential role as adjunctive tools — not replacements — for existing prognostic systems. ......The clinical impact of these AUC improvements remains to be established through prospective validation"（Page 22 Line 449-460）.
(2) Clinical applicability expanded: We have added discussion of context-dependent use: "The sensitivity–specificity trade-offs in Table 3 reveal **score-specific** rather than uniform clinical utility. When combined with **SAPS II**, RDW improved sensitivity from 76.3% to **81.7%** (+5.4 percentage points), reducing false-negative classifications in early screening. When combined with **SOFA**, RDW increased specificity from 60.8% to **80.3%** (+19.5 percentage points), substantially reducing false-positive rates for resource allocation decisions. These divergent profiles suggest that RDW shifts the operating point of the host score: toward higher sensitivity for triage when added to SAPS II, and toward higher specificity for confirmation when added to SOFA. **The modest AUC increments (0.68–1.61%) therefore understate the clinically meaningful optimisation at specific decision thresholds. The clinical utility of this score-specific optimisation requires prospective validation.**" (Page 21-22, Line Line 437-449)
(3) Per-SD framework: We have added the per-SD reporting framework, which provides clinically interpretable effect sizes. (Page 13-14)
(4) Threshold analysis: Table 3 now presents threshold effects with clinically actionable cut-points.

**Major Comment 2: Additional Confounders**

**Comment:** *"Important ICU variables may still be missing (e.g., infection status, vasopressor dose, liver-specific complications severity). Recommendation: Provide a full list of adjusted covariates in the main manuscript (not only supplementary). Discuss potential residual confounding more explicitly."*

**Response:** We agree that residual confounding from unmeasured or incompletely measured variables is an important concern. (1) The Methods now explicitly lists all covariates included in the fully adjusted model: "**The covariates adjusted for in the final fully adjusted model included sex, age, race, BMI, Charlson Comorbidity Index, vasopressin use, albumin use, mechanical ventilation, renal replacement therapy, hemoglobin, hematocrit, albumin, potassium, and lactate levels. For ratio-based biomarkers, their constituent laboratory variables were excluded from the respective adjustment models to avoid overadjustment and collinearity (hemoglobin was excluded from the HRR model; albumin was excluded from the RAR model).**" (Page 10, Line 192-195). (2) Residual confounding discussion: The Discussion now states: "Fifth, the absence of granular treatment-regimen data (e.g., specific vasopressor dosing, antibiotic protocols, transfusion thresholds) and cause-specific mortality information in our dataset precludes deeper mechanistic and outcome analyses." (Page 24, Line 496-499) (3) E-values: Supplementary Table 5 reports both per-unit and per-SD E-values, quantifying the minimum strength of association an unmeasured confounder would need to nullify our findings. The per-SD E-values (RDW 1.85, RAR 1.47, RPR 1.51, HRR 2.52) indicate moderate robustness to unmeasured confounding.

**Major Comment 3: Lack of External Validation**

**Comment:** *"The study is based on a single-centre database (MIMIC-IV), which limits generalisability. Recommendation: Emphasise this limitation more strongly."*

**Response:** We fully agree. The single-center nature of MIMIC-IV is an important limitation that we have now emphasised more strongly in the Limitations section.

**Action taken:** The Limitations section now reads: "Second, as a single-center study, our findings may not be generalizable to other healthcare systems or patient populations; multicenter external validation is essential before clinical implementation. Third, our cohort inherently represents the sickest cirrhosis patients requiring critical care, excluding those managed on general wards or in palliative settings. While this limits applicability to non-critical populations, it accurately reflects the intended target population for biomarker prognostication in the ICU." (Page 23 Line 481-488)

**Major Comment 4: Biomarker Timing and Dynamics**

**Comment:** *"Only baseline (first 24h ICU) values were used. Dynamic changes in RDW and related indices may be more prognostically informative. Recommendation: Discuss the limitation more critically. Suggest longitudinal analyses as a key next step."*

**Response:** We completely agree. The exclusive use of baseline values is a significant limitation that we have now discussed more critically, linking it to our choice of the 30-day endpoint. The Discussion now states: "Fourth, we examined only baseline biomarker values measured within the first 24 hours of ICU admission. Dynamic changes during the ICU stay — particularly within the first 30 days — may capture evolving inflammatory status and treatment response beyond what static measurements can offer. Future studies should evaluate longitudinal RDW trajectories and delta-RDW (change from baseline) to determine whether time-varying biomarker values enhance predictive performance. " (Page 23-24 Line 488-495)

**Major Comment 5: Overstatement in Conclusions**

**Comment:** *"The conclusion suggests these biomarkers may "optimise patient management and resource allocation," which is not directly demonstrated. Recommendation: Tone down conclusions to reflect associative findings, not clinical impact."*

**Response:** We fully agree and have comprehensively revised the Conclusion to reflect only associative findings, removing all claims that are not directly supported by the data. The revised Conclusion states: "In conclusion, our data suggest that RDW-related biomarkers are independently associated with 30-day mortality in ICU patients with cirrhosis. RDW and HRR demonstrated the strongest per-SD associations among the four indices, though standalone discrimination was modest. The integration of these indices into clinical practice could provide modest additional prognostic information when combined with established scoring systems. Further prospective validation, external validation, and investigation of dynamic biomarker changes are required before these findings can inform clinical decision-making." All previous claims about "optimising patient management and resource allocation" have been removed from the Abstract, Discussion, and Conclusion.

**Minor Comments (Reviewer #2)**

**Response:** All minor comments have been thoroughly addressed:

(1)Language and grammar: The entire manuscript has undergone professional English editing. Specific corrections include: "patients with cirrhotic" corrected to "patients with cirrhosis" throughout; inconsistent tense usage resolved; and all grammatical errors corrected.
(2) Redundancy: The Introduction has been condensed by removing repetitive background on RDW and cirrhosis. The Discussion has been streamlined by reducing reiteration of known literature and focusing on novel findings and their interpretation.
(3) ROC interpretation: All wording regarding ROC analysis has been made more cautious.

(4) Figures and tables: All ROC curves now include clear labels for each biomarker Supplementary materials have been reformatted for improved readability.
(5) Ethics statement: The ethics statement now appears exclusively in the Methods section (under "Data"). The online submission form has been updated to ensure consistency with the manuscript text.

We sincerely thank the Academic Editor and both Reviewers for their thorough and constructive feedback. These revisions have substantially strengthened the manuscript. We believe the revised manuscript now meets the standards for publication in PLOS ONE and respectfully request reconsideration.

Sincerely,

The Authors

Xiaodong Zhu, Chanchan Lin, Xiaoqiang Liu, Zicheng Huang, Yingyi Li

Department of Gastroenterology, The First Hospital of Quanzhou

Affiliated to Fujian Medical University, Quanzhou, China
