# Supplementary material for: Association between Red Cell Distribution Width (RDW)-related inflammatory biomarkers and prognosis in ICU Cirrhosis patients: Evidence from the MIMIC-IV
Source: PLoS One. 2026 Jun 26;21(6):e0352023. doi: 10.1371/journal.pone.0352023 (PMC13308776; doi:10.1371/journal.pone.0352023)
Supplement: Peer review history

# Peer review: Decision Letter 0

**DOI:** 10.1371/journal.pone.0352023.r001

**Authors:** Elizabeth Mayne

31 Mar 2026

PONE-D-25-67953

Association between Red Cell Distribution Width (RDW)-Related Inflammatory Biomarkers and Prognosis in ICU Cirrhosis Patients: Evidence from MIMIC-IV

PLOS One

Dear Dr. Li,

Thank you for submitting your manuscript to PLOS ONE. After careful consideration, we feel that it has merit but does not fully meet PLOS ONE’s publication criteria as it currently stands. Therefore, we invite you to submit a revised version of the manuscript that addresses the points raised during the review process.

Both reviewers found this interesting but there were concerns about:

1. The frequency of testing (only baseline data)

2. Missing laboratory parameters known to be important in outcomes in ICU mortality

3. Overstatement of clinical extrapolation - the AUC was showed mild predictive value and both reviewers felt that this would not translate directly to clinical significance.

Please submit your revised manuscript by May 15 2026 11:59PM. If you will need more time than this to complete your revisions, please reply to this message or contact the journal office at plosone@plos.org. When you're ready to submit your revision, log on to https://www.editorialmanager.com/pone/ and select the 'Submissions Needing Revision' folder to locate your manuscript file.

Please include the following items when submitting your revised manuscript:

- A letter that responds to each point raised by the academic editor and reviewer(s). You should upload this letter as a separate file labeled 'Response to Reviewers'.
- A marked-up copy of your manuscript that highlights changes made to the original version. You should upload this as a separate file labeled 'Revised Manuscript with Track Changes'.
- An unmarked version of your revised paper without tracked changes. You should upload this as a separate file labeled 'Manuscript'.

If you would like to make changes to your financial disclosure, please include your updated statement in your cover letter. Guidelines for resubmitting your figure files are available below the reviewer comments at the end of this letter.

If applicable, we recommend that you deposit your laboratory protocols in protocols.io to enhance the reproducibility of your results. Protocols.io assigns your protocol its own identifier (DOI) so that it can be cited independently in the future. For instructions see: https://journals.plos.org/plosone/s/submission-guidelines#loc-laboratory-protocols. Additionally, PLOS ONE offers an option for publishing peer-reviewed Lab Protocol articles, which describe protocols hosted on protocols.io. Read more information on sharing protocols at https://plos.org/protocols?utm_medium=editorial-email&utm_source=authorletters&utm_campaign=protocols.

As the corresponding author, your ORCID iD is verified in the submission system and will appear in the published article. PLOS supports the use of ORCID, and we encourage all coauthors to register for an ORCID iD and use it as well. Please encourage your coauthors to verify their ORCID iD within the submission system before final acceptance, as unverified ORCID iDs will not appear in the published article. *Only*   the individual author can complete the verification step; PLOS staff *cannot*   verify ORCID iDs on behalf of authors.

We look forward to receiving your revised manuscript.

Kind regards,

Elizabeth S. Mayne, M.D.

Academic Editor

PLOS One

Journal Requirements:

When submitting your revision, we need you to address these additional requirements.

1. Please ensure that your manuscript meets PLOS ONE's style requirements, including those for file naming. The PLOS ONE style templates can be found at

https://journals.plos.org/plosone/s/file?id=wjVg/PLOSOne_formatting_sample_main_body.pdf and https://journals.plos.org/plosone/s/file?id=ba62/PLOSOne_formatting_sample_title_authors_affiliations.pdf

2. Please remove any funding-related text from the manuscript. Funding information should not appear in any section or other areas of your manuscript. We will only publish funding information present in the Funding Statement section of the online submission form.

3. Please note that your Data Availability Statement is currently missing the repository name and/or the DOI/accession number of each dataset. If your manuscript is accepted for publication, you will be asked to provide these details on a very short timeline. We therefore suggest that you provide this information now, though we will not hold up the peer review process if you are unable.

4. Your ethics statement should only appear in the Methods section of your manuscript. If your ethics statement is written in any section besides the Methods, please move it to the Methods section and delete it from any other section. Please ensure that your ethics statement is included in your manuscript, as the ethics statement entered into the online submission form will not be published alongside your manuscript.

5. Please include captions for your Supporting Information files at the end of your manuscript, and update any in-text citations to match accordingly. Please see our Supporting Information guidelines for more information: http://journals.plos.org/plosone/s/supporting-information.

If the reviewer comments include a recommendation to cite specific previously published works, please review and evaluate these publications to determine whether they are relevant and should be cited. There is no requirement to cite these works unless the editor has indicated otherwise.

[Note: HTML markup is below. Please do not edit.]

Reviewers' comments:

Reviewer's Responses to Questions

**Comments to the Author**

1. Is the manuscript technically sound, and do the data support the conclusions?

The manuscript must describe a technically sound piece of scientific research with data that supports the conclusions. Experiments must have been conducted rigorously, with appropriate controls, replication, and sample sizes. The conclusions must be drawn appropriately based on the data presented.

Reviewer #1: Partly

Reviewer #2: Partly

**********

2. Has the statistical analysis been performed appropriately and rigorously?

Reviewer #1: No

Reviewer #2: Yes

**********

3. Have the authors made all data underlying the findings in their manuscript fully available?

The PLOS Data policy requires authors to make all data underlying the findings described in their manuscript fully available without restriction, with rare exception (please refer to the Data Availability Statement in the manuscript PDF file). The data should be provided as part of the manuscript or its supporting information, or deposited to a public repository. For example, in addition to summary statistics, the data points behind means, medians and variance measures should be available. If there are restrictions on publicly sharing data—e.g. participant privacy or use of data from a third party—those must be specified.

Reviewer #1: Yes

Reviewer #2: Yes

**********

4. Is the manuscript presented in an intelligible fashion and written in standard English?

PLOS ONE does not copyedit accepted manuscripts, so the language in submitted articles must be clear, correct, and unambiguous. Any typographical or grammatical errors should be corrected at revision, so please note any specific errors here.

Reviewer #1: Yes

Reviewer #2: Yes

**********

5. Review Comments to the Author

Please use the space provided to explain your answers to the questions above. You may also include additional comments for the author, including concerns about dual publication, research ethics, or publication ethics. (Please upload your review as an attachment if it exceeds 20,000 characters)

Reviewer #1: Summary

This retrospective cohort study using the MIMIC-IV database evaluates the association between RDW and RDW-derived indices (RAR, RPR, HRR) measured within 24 hours of ICU admission and short- and long-term mortality in critically ill patients with cirrhosis. The authors report statistically significant associations across multiple mortality endpoints and modest discrimination (AUC ~0.54–0.64), with small incremental improvements when adding RDW to established severity scores.

The topic is clinically relevant, and the dataset is appropriate. However, several important methodological and interpretative issues limit the strength of the conclusions in its current form. These concerns primarily relate to scaling and interpretability of effect sizes, potential overadjustment and collinearity, modeling of in-hospital mortality, and overstatement of clinical utility given modest predictive performance.

Substantial revision is recommended.

Major Comments

1. Interpretation and Scaling of Ratio Variables (Especially HRR)

The adjusted hazard ratios reported for HRR (~0.01 across endpoints) imply an implausibly large protective effect per unit increase. Given the narrow expected distribution of HRR, this likely reflects scaling or collinearity issues rather than a true biological magnitude of effect.

Although units are presented in Table 1, the manuscript does not clarify:

- The exact units used in constructing ratio variables

- The distribution and range of each ratio

- What a “1-unit increase” represents clinically

- Whether any transformations were applied

Hazard ratios should be reported per standard deviation or per interquartile range to improve interpretability. Additionally, variance inflation factor (VIF) analysis or other collinearity diagnostics should be provided, particularly because hemoglobin is adjusted for in models evaluating HRR.

Without clarification, the HRR results are difficult to interpret and potentially misleading.

2. Potential Overadjustment and Collinearity

The fully adjusted models include laboratory variables that are components of the derived indices (e.g., hemoglobin in models evaluating HRR; albumin and platelets in models evaluating RAR and RPR).

This raises concerns regarding:

- Collinearity

- Overadjustment

- Instability of regression coefficients

Sensitivity analyses excluding component variables from adjustment models would strengthen confidence in the reported associations.

3. Modeling of In-Hospital Mortality

In-hospital mortality is analyzed using Cox regression without detailed description of time origin, censoring assumptions, or consideration of discharge alive as a competing event.

Because discharge alive precludes subsequent in-hospital death, this is technically a competing risks scenario. At minimum, the authors should clarify the time-to-event structure and justify the use of standard Cox regression.

4. Modest Predictive Performance and Overstatement of Clinical Utility

The reported AUC values for RDW and derived indices range from approximately 0.54 to 0.64, indicating weak to moderate discrimination.

Although incremental AUC improvements when adding RDW to SAPS II, MELD, or SOFA are statistically significant, they are small (<0.02). The clinical relevance of such marginal improvements is uncertain (likely negligible).

Accordingly, statements suggesting that these biomarkers “enhance clinical decision-making” or “optimize resource allocation” should be tempered. The data support statistical association and modest incremental prognostic information, but not strong standalone predictive utility.

5. Cohort Derivation and Missing Data

Patients without RDW or derived index measurements within 24 hours were excluded. The manuscript does not report:

The number excluded for missing laboratory values

A flow diagram of cohort derivation

Comparison of included vs excluded patients

Given that laboratory testing intensity in ICU populations correlates with illness severity, exclusion based on missing laboratory data may introduce selection bias. Greater transparency in cohort derivation is required.

Minor Comments

Consider modeling biomarkers using restricted cubic splines to assess non-linearity rather than dichotomizing at the median in Kaplan–Meier analyses.

The limitations section should explicitly acknowledge:

- Modest discrimination

- Lack of external validation

- Potential collinearity and scaling concerns

Clarify laboratory units and ensure consistency (e.g., albumin in g/dL vs g/L).

Subgroup analyses appear exploratory; clarify whether interaction terms were formally tested and whether correction for multiple comparisons was considered.

Several grammatical and formatting issues should be corrected for clarity.

Overall Recommendation

The manuscript addresses a clinically relevant question using a well-established database. The associations observed are generally statistically robust. However, concerns regarding scaling and interpretation of ratio variables, potential overadjustment, modest predictive performance, and overstated clinical implications require substantial revision before the work can be considered for publication.

With careful methodological clarification and more restrained interpretation, this study could provide a useful contribution to the literature on readily available prognostic markers in ICU cirrhosis.

Reviewer #2: This manuscript presents a retrospective cohort study evaluating the prognostic value of the red cell distribution width (RDW) and related biomarkers (RDW-to-albumin ratio (RAR), RDW-to-platelet ratio (RPR), and

haemoglobin-to-RDW ratio (HRR)) in ICU patients with cirrhosis using the MIMIC-IV database. The topic is clinically relevant, as prognostic stratification in critically ill cirrhosis patients remains a major challenge. The use of a large, well-established database and multiple mortality endpoints (in-hospital, 90-day, 365-day) strengthens the study.

The authors demonstrate statistically significant associations between RDW-related indices and mortality outcomes, with RDW and HRR showing the strongest predictive value. However, while the study is methodologically comprehensive, there are several concerns regarding interpretation, clinical relevance, and presentation that should be addressed before the manuscript can be considered for publication.

**********

6. PLOS authors have the option to publish the peer review history of their article (what does this mean?). If published, this will include your full peer review and any attached files.

If you choose “no”, your identity will remain anonymous but your review may still be made public.

**Do you want your identity to be public for this peer review?**   For information about this choice, including consent withdrawal, please see our Privacy Policy.

Reviewer #1: **Yes:**   Jody Rusch

Reviewer #2: No

**********

[NOTE: If reviewer comments were submitted as an attachment file, they will be attached to this email and accessible via the submission site. Please log into your account, locate the manuscript record, and check for the action link "View Attachments". If this link does not appear, there are no attachment files.]

To ensure your figures meet our technical requirements, please review our figure guidelines: https://journals.plos.org/plosone/s/figures

You may also use PLOS’s free figure tool, NAAS, to help you prepare publication quality figures: https://journals.plos.org/plosone/s/figures#loc-tools-for-figure-preparation.

NAAS will assess whether your figures meet our technical requirements by comparing each figure against our figure specifications.

Attachment Submitted filename: Reviewer Report PONE_D_25_67953.docx

Attachment Submitted filename: PONE-D-25-67953_reviewer RDW Reviewer comments.pdf

# Author comment: Author response to Decision Letter 1

**DOI:** 10.1371/journal.pone.0352023.r002

14 May 2026

PONE-D-25-67953

Association between Red Cell Distribution Width (RDW)-Related Inflammatory Biomarkers and Prognosis in ICU Cirrhosis Patients: Evidence from MIMIC-IV

PLOS One

Dear editor Elizabeth S. Mayne and reviewers,

We sincerely thank the academic editor and the two reviewers for their thorough, constructive, and insightful suggestions. We have carefully considered all the recommendations and have undertaken substantial revisions to the manuscript. The two most important methodological refinements are as follows: (1) the primary short-term endpoint was changed from in-hospital mortality to 30-day mortality, thereby eliminating the issues arising from competing risk and informative censoring; and (2) Z-score standardization was adopted, with both unit hazard ratios and per-standard-deviation hazard ratios reported, thereby addressing the inherent scaling and interpretability challenges of ratio-derived biomarkers. All results have been reanalyzed using the updated endpoints and incorporating the standardized indices.

Below, we provide a point-by-point response to each comment raised by the editor, the journal requirements, and the two reviewers. All line and page numbers refer to the revised manuscript. Modifications made in response to specific comments have been highlighted in yellow in the accompanying "Revised Manuscript with Tracked Changes" file.

EDITOR COMMENTS

Editor Comment 1: Frequency of testing (only baseline data)

Comment: "Both reviewers found this interesting but there were concerns about: The frequency of testing (only baseline data)."

Response: We fully acknowledge this important limitation. Our study exclusively used biomarker values measured within the first 24 hours of ICU admission, which precludes assessment of whether dynamic changes in RDW-related indices during the ICU stay provide additional prognostic information beyond baseline values. This limitation has now been substantially expanded in the Discussion.In the Discussion, we have added: "Fourth, we examined only baseline biomarker values measured within the first 24 hours of ICU admission. Dynamic changes during the ICU stay — particularly within the first 30 days — may capture evolving inflammatory status and treatment response beyond what static measurements can offer. Future studies should evaluate longitudinal RDW trajectories and delta-RDW (change from baseline) to determine whether time-varying biomarker values enhance predictive performance." We have also added this caveat to the Abstract. (Page 23-24, Line 486-493)

Editor Comment 2: Missing laboratory parameters

Comment: "Both reviewers found this interesting but there were concerns about: Missing laboratory parameters known to be important in outcomes in ICU mortality."

Response: We agree that important ICU variables were not fully captured in our models, potentially resulting in residual confounding. We have addressed this in three ways: (1) explicitly discussing residual confounding in the Discussion; (2) reporting E-values (both per-unit and per-SD) to assess robustness to unmeasured confounding (Supplementary Table 2); and (3) providing a comprehensive list of all adjusted covariates in the Methods. In the Discussion, we have added: “Fifth, important ICU variables such as documented infection status, specific vasopressor dosing regimens, and granular severity assessments of liver-specific complications were not fully captured in our models, potentially resulting in residual confounding” (Page 24, Line494-497). The E-values reported in Supplementary Table 5 provide quantitative assessment of how strong an unmeasured confounder would need to be to nullify our findings (Page 16, Line 326-330). In the Methods, we now explicitly list all covariates included in the fully adjusted model (Page 10, Line 192-199).

Editor Comment 3: Overstatement of clinical extrapolation

Comment: "Both reviewers found this interesting but there were concerns about: Overstatement of clinical extrapolation — the AUC was showed mild predictive value and both reviewers felt that this would not translate directly to clinical significance."

Response: We completely agree with this critique. The original manuscript overstated the clinical utility of RDW-related biomarkers given their modest standalone discrimination. We have comprehensively revised the tone throughout the manuscript, removing all unsupported claims and replacing them with appropriately cautious, evidence-based statements. (1) Abstract: The Conclusion now states: "RDW-related biomarkers demonstrate statistically significant but modest associations with mortality in ICU cirrhosis patients. RDW and HRR showed the strongest predictive capabilities among the four indices, though standalone discrimination was limited. These accessible, cost-effective biomarkers may serve as adjunctive prognostic markers when combined with established scoring systems. However, further prospective validation is required before clinical implementation, and dynamic changes in these biomarkers warrant investigation in future studies." (Page 3 Line44-52). (2) Discussion: We have added: " While the standalone AUCs for RDW-derived biomarkers were modest (≤63.7%), their incremental value when combined with established scores (AUC improvement 0.68–1.6%), combined with their cost-effectiveness and universal availability in routine laboratories, supports their potential role as adjunctive tools — not replacements — for existing prognostic systems. ...... The clinical impact of these AUC improvements remains to be established through prospective validation. " (Page 22, Line 449-460). (3) Conclusion: All claims about "optimising patient management and resource allocation" have been removed (Page 24-25, Line505-514).

JOURNAL REQUIREMENTS

Requirement 1: PLOS ONE style requirements

Comment: "Please ensure that your manuscript meets PLOS ONE's style requirements, including those for file naming."

Response: We have carefully reviewed the PLOS ONE formatting guidelines and templates, and have ensured that the revised manuscript adheres to all style requirements, including font, spacing, heading formats, and figure/table numbering conventions.

Requirement 2: Remove funding-related text from manuscript

Comment: "Please remove any funding-related text from the manuscript. Funding information should not appear in any section or other areas of your manuscript."

Response: We have removed all funding-related text from the manuscript body. The Funding section in the Declarations has been replaced with "This research received no specific grant from any funding agency." All funding details now appear only in the Funding Statement section of the online submission form, as required.

Requirement 3: Data Availability Statement (missing DOI/accession number)

Comment: "Please note that your Data Availability Statement is currently missing the repository name and/or the DOI/accession number of each dataset."

Response: We thank the Editor for this important correction. The data underlying this study are from the Medical Information Mart for Intensive Care IV (MIMIC-IV) database, version 3.0, available through PhysioNet (https://physionet.org/content/mimiciv/3.0/).

Requirement 4: Ethics statement location

Comment: "Your ethics statement should only appear in the Methods section of your manuscript. If your ethics statement is written in any section besides the Methods, please move it to the Methods section and delete it from any other section."

Response: We have removed the Ethics Statement from the Declarations section. The ethics information now appears exclusively in the Methods section (under "Data"), as required (Page 6, Line 104-111).

Requirement 5: Supporting Information captions

Comment: "Please include captions for your Supporting Information files at the end of your manuscript, and update any in-text citations to match accordingly."

Response: We have added complete captions for all Supporting Information files at the end of the revised manuscript and have updated all in-text citations to ensure consistency (Page 25).

REVIEWER #1 (Dr. Jody Rusch)

We sincerely thank Dr. Rusch for the detailed and methodologically rigorous review. The comments on scaling, collinearity, and endpoint selection have been particularly influential in shaping our revisions. Below, we address each comment in detail.

Major Comment 1: Interpretation and Scaling of Ratio Variables (Especially HRR)

Comment: "The adjusted hazard ratios reported for HRR (~0.01 across endpoints) imply an implausibly large protective effect per unit increase. Given the narrow expected distribution of HRR, this likely reflects scaling or collinearity issues rather than a true biological magnitude of effect. Hazard ratios should be reported per standard deviation or per interquartile range to improve interpretability. Additionally, variance inflation factor (VIF) analysis or other collinearity diagnostics should be provided. Without clarification, the HRR results are difficult to interpret and potentially misleading."

Response: We fully agree with this critical methodological concern. The per-unit HR for HRR (HR 0.01) is indeed misleading because HRR has a very narrow distribution (mean 0.6, SD 0.2), where a "one-unit increase" represents a change from a typical value to one 3-5 SDs above the mean -- not a clinically meaningful increment. To resolve this, we have implemented a comprehensive z-score standardisation approach with dual HR reporting: (1) Z-score standardisation: Each biomarker was converted to a z-score (z = [value - mean] / SD) using the cohort mean and standard deviation. Parallel Cox models were fitted using both original-scale variables (yielding per-unit HRs) and z-scored variables (yielding per-SD HRs) (Page 7-8, Line 133-139). (2) Dual reporting in Table 2: The revised Table 2 now presents both per-unit HRs (for comparability with prior literature) and per-SD HRs (for clinically interpretable cross-biomarker comparisons). The per-SD HRs for 30-day mortality are: RDW (HR 1.41, 95% CI 1.30-1.52), RAR (HR 1.17, 95% CI 1.11-1.24), RPR (HR 1.19, 95% CI 1.11-1.28), and HRR (HR 0.52, 95% CI 0.44-0.61) (Page 13, Line 264-269). The per-SD HRR of 0.52 (48% risk reduction per SD) is clinically interpretable and consistent with inflammatory biomarker literature. In addition, we provide the VIF analysis results in the table below. All covariates in the fully adjusted model show no collinearity with the RDW-related indicators. As shown in the table, except for hemoglobin and hematocrit, the VIF values of all other covariates are less than 5. Hemoglobin and hematocrit have a VIF greater than 5 due to collinearity between them, but neither exhibits collinearity with RDW, RAR, or RPR. In the adjustment of the HRR model, the hemoglobin variable has been excluded, and the VIF of hematocrit is less than 5, indicating no collinearity with HRR.

VIF analysis results:

Major Comment 2: Potential Overadjustment and Collinearity

Comment: "The fully adjusted models include laboratory variables that are components of the derived indices (e.g., hemoglobin in models evaluating HRR; albumin and platelets in models evaluating RAR and RPR). This raises concerns regarding collinearity, overadjustment, and instability of regression coefficients. Sensitivity analyses excluding component variables from adjustment models would strengthen confidence in the reported associations."

Response: Thank you for your suggestion. In the Methods section, we have listed all covariates included in the fully adjusted models. For each model, we have excluded the laboratory variables that constitute the derived indices (e.g., hemoglobin was excluded in the model assessing HRR; albumin was excluded in the model assessing RAR). Therefore, issues such as collinearity, over-adjustment, or instability of regression coefficients resulting from this problem do not exist. (Page 10, Line 192-199)

Major Comment 3: Modeling of In-Hospital Mortality

Comment: "In-hospital mortality is analyzed using Cox regression without detailed description of time origin, censoring assumptions, or consideration of discharge alive as a competing event. Because discharge alive precludes subsequent in-hospital death, this is technically a competing risks scenario. At minimum, the authors should clarify the time-to-event structure and justify the use of standard Cox regression."

Response: We thank Dr. Rusch for raising this critical methodological concern. Upon careful reflection, we agree that in-hospital mortality presents fundamental statistical challenges that are difficult to resolve satisfactorily: (i) hospital length of stay varies widely, creating differential observation periods; (ii) discharge alive constitutes a competing event that precludes subsequent in-hospital death; and (iii) discharge is non-random with respect to prognosis, violating the non-informative censoring assumption. Rather than applying competing risks regression (Fine-Gray) to an endpoint where the competing event (discharge) is partially determined by the biomarkers themselves, we have taken the more rigorous approach of changing the primary short-term endpoint from in-hospital mortality to 30-day mortality. (1) Endpoint change: The primary short-term endpoint has been changed from in-hospital mortality to 30-day mortality. (2) All results re-calculated: Tables 2-4 and Figures 1-3 have been completely re-analysed using the 30-day endpoint. The revised Results now report 30-day mortality as the primary endpoint, with 90-day and 365-day mortality as secondary endpoints.

Major Comment 4: Modest Predictive Performance and Overstatement of Clinical Utility

Comment: "The reported AUC values for RDW and derived indices range from approximately 0.54 to 0.64, indicating weak to moderate discrimination. Although incremental AUC improvements when adding RDW to SAPS II, MELD, or SOFA are statistically significant, they are small (<0.02). The clinical relevance of such marginal improvements is uncertain (likely negligible). Accordingly, statements suggesting that these biomarkers "enhance clinical decision-making" or "optimize resource allocation" should be tempered."

Response: We completely agree with this assessment. The original manuscript overstated the clinical utility of these biomarkers. We have comprehensively revised the Discussion and Conclusion to adopt appropriately cautious language that accurately reflects the modest predictive performance and uncertain clinical relevance of marginal AUC improvements. (1) Discussion (revised): "While the standalone AUCs for RDW-derived biomarkers were modest (≤63.7%), their incremental value when combined with established scores (AUC improvement 0.68–1.6%), combined with their cost-effectiveness and universal availability in routine laboratories, supports their potential role as adjunctive tools — not replacements — for existing prognostic systems." (Page 22, Line 449-454) (2) Conclusion (revised): "In conclusion, our data suggest that RDW-related biomarkers are independently associated with 30-day mortality in ICU patients with cirrhosis. RDW and HRR demonstrated the strongest per-SD associations among the four indices, though standalone discrimination was modest. The integration of these indices into clinical practice could provide modest additional prognostic information when combined with established scoring systems. Further prospective validation, external validation, and investigation of dynamic biomarker changes are required before these findings can inform clinical decision-making." (Page 24-25, Line 505-514) (3) All unsupported claims removed: Phrases such as "enhance clinical decision-making", "optimise patient management", and "optimise resource allocation" have been completely removed from the Abstract, Discussion, and Conclusion.

Major Comment 5: Cohort Derivation and Missing Data

Comment: "Patients without RDW or derived index measurements within 24 hours were excluded. The manuscript does not report: The number excluded for missing laboratory values; A flow diagram of cohort derivation; Comparison of included vs excluded patients. Given that laboratory testing intensity in ICU populations correlates with illness severity, exclusion based on missin

Attachment Submitted filename: Response to Reviewers.docx

# Peer review: Decision Letter 1

**DOI:** 10.1371/journal.pone.0352023.r003

**Authors:** Elizabeth Mayne, Elizabeth Mayne

4 Jun 2026

Association between Red Cell Distribution Width (RDW)-Related Inflammatory Biomarkers and Prognosis in ICU Cirrhosis Patients: Evidence from MIMIC-IV

PONE-D-25-67953R1

Dear Dr. Li,

We’re pleased to inform you that your manuscript has been judged scientifically suitable for publication and will be formally accepted for publication once it meets all outstanding technical requirements.

Within one week, you’ll receive an e-mail detailing the required amendments. When these have been addressed, you’ll receive a formal acceptance letter and your manuscript will be scheduled for publication.

An invoice will be generated when your article is formally accepted. Please note, if your institution has a publishing partnership with PLOS and your article meets the relevant criteria, all or part of your publication costs will be covered. Please make sure your user information is up-to-date by logging into Editorial Manager at Editorial Manager® and clicking the ‘Update My Information' link at the top of the page. For questions related to billing, please contact billing support.

If your institution or institutions have a press office, please notify them about your upcoming paper to help maximize its impact. If they’ll be preparing press materials, please inform our press team as soon as possible -- no later than 48 hours after receiving the formal acceptance. Your manuscript will remain under strict press embargo until 2 pm Eastern Time on the date of publication. For more information, please contact onepress@plos.org.

Kind regards,

Elizabeth S. Mayne, M.D.

Academic Editor

PLOS One

Additional Editor Comments (optional):

Reviewers' comments:

Reviewer's Responses to Questions

**Comments to the Author**

1. If the authors have adequately addressed your comments raised in a previous round of review and you feel that this manuscript is now acceptable for publication, you may indicate that here to bypass the “Comments to the Author” section, enter your conflict of interest statement in the “Confidential to Editor” section, and submit your "Accept" recommendation.

Reviewer #1: All comments have been addressed

Reviewer #2: All comments have been addressed

**********

2. Is the manuscript technically sound, and do the data support the conclusions?

The manuscript must describe a technically sound piece of scientific research with data that supports the conclusions. Experiments must have been conducted rigorously, with appropriate controls, replication, and sample sizes. The conclusions must be drawn appropriately based on the data presented.

Reviewer #1: (No Response)

Reviewer #2: Yes

**********

3. Has the statistical analysis been performed appropriately and rigorously?

Reviewer #1: (No Response)

Reviewer #2: Yes

**********

4. Have the authors made all data underlying the findings in their manuscript fully available?

The PLOS Data policy requires authors to make all data underlying the findings described in their manuscript fully available without restriction, with rare exception (please refer to the Data Availability Statement in the manuscript PDF file). The data should be provided as part of the manuscript or its supporting information, or deposited to a public repository. For example, in addition to summary statistics, the data points behind means, medians and variance measures should be available. If there are restrictions on publicly sharing data—e.g. participant privacy or use of data from a third party—those must be specified.

Reviewer #1: (No Response)

Reviewer #2: Yes

**********

5. Is the manuscript presented in an intelligible fashion and written in standard English?

PLOS ONE does not copyedit accepted manuscripts, so the language in submitted articles must be clear, correct, and unambiguous. Any typographical or grammatical errors should be corrected at revision, so please note any specific errors here.

Reviewer #1: (No Response)

Reviewer #2: Yes

**********

6. Review Comments to the Author

Please use the space provided to explain your answers to the questions above. You may also include additional comments for the author, including concerns about dual publication, research ethics, or publication ethics. (Please upload your review as an attachment if it exceeds 20,000 characters)

Reviewer #1: (No Response)

Reviewer #2: Reviewer Report – PLOS ONE

Manuscript ID: PONE-D-25-67953

Title: Association between Red Cell Distribution Width (RDW)-Related Inflammatory Biomarkers and Prognosis in ICU Cirrhosis Patients: Evidence from MIMIC-IV

Final Review Report

The authors have addressed the concerns raised in the previous round of review, and the revised manuscript has improved substantially in methodological rigor, clarity of reporting, and balance of interpretation.

In particular, the revisions appropriately addressed:

•Addition of sensitivity analyses and expanded discussion of residual confounding and external validity.

•More cautious and appropriate interpretation of ROC/AUC findings and clinical applicability.

•Expanded discussion regarding the limitations of retrospective single-centre analyses and the need for prospective validation.

The discussion and conclusion sections are now more appropriately tempered and aligned with the observed predictive performance. The manuscript no longer overstates the clinical applicability of the findings, and the limitations are adequately acknowledged.

Overall, I believe the authors have satisfactorily addressed my comments and that the manuscript is suitable for publication in its revised form.

**********

7. PLOS authors have the option to publish the peer review history of their article (what does this mean?). If published, this will include your full peer review and any attached files.

If you choose “no”, your identity will remain anonymous but your review may still be made public.

**Do you want your identity to be public for this peer review?**   For information about this choice, including consent withdrawal, please see our Privacy Policy.

Reviewer #1: **Yes:**   Jody Rusch

Reviewer #2: **Yes:**   Dr Susan Louw (PhD), University of the Witwatersrand, Johannesburg, South Africa

**********

# Editor report: Acceptance letter

**DOI:** 10.1371/journal.pone.0352023.r004

**Authors:** Elizabeth Mayne, Elizabeth Mayne

PONE-D-25-67953R1

PLOS One

Dear Dr. Li,

I'm pleased to inform you that your manuscript has been deemed suitable for publication in PLOS One. Congratulations! Your manuscript is now being handed over to our production team.

At this stage, our production department will prepare your paper for publication. This includes ensuring the following:

* All references, tables, and figures are properly cited

* All relevant supporting information is included in the manuscript submission,

* There are no issues that prevent the paper from being properly typeset

You will receive further instructions from the production team, including instructions on how to review your proof when it is ready. Please keep in mind that we are working through a large volume of accepted articles, so please give us a few days to review your paper and let you know the next and final steps.

Lastly, if your institution or institutions have a press office, please let them know about your upcoming paper now to help maximize its impact. If they'll be preparing press materials, please inform our press team within the next 48 hours. Your manuscript will remain under strict press embargo until 2 pm Eastern Time on the date of publication. For more information, please contact onepress@plos.org.

You will receive an invoice from PLOS for your publication fee after your manuscript has reached the completed accept phase. If you receive an email requesting payment before acceptance or for any other service, this may be a phishing scheme. Learn how to identify phishing emails and protect your accounts at https://explore.plos.org/phishing.

If we can help with anything else, please email us at customercare@plos.org.

Thank you for submitting your work to PLOS ONE and supporting open access.

Kind regards,

PLOS ONE Editorial Office Staff

on behalf of

Dr. Elizabeth S. Mayne

Academic Editor

PLOS One
